# Optimizing dietary rumen-degradable starch to rumen-degradable protein ratio improves lactation performance and nitrogen utilization efficiency in mid-lactating Holstein dairy cows

**DOI:** 10.3389/fvets.2024.1330876

**Published:** 2024-02-29

**Authors:** Panliang Chen, Yan Li, Meimei Wang, Yizhao Shen, Mingchao Liu, Hongjian Xu, Ning Ma, Yufeng Cao, Qiufeng Li, Mahmoud M. Abdelsattar, Zhiyuan Wang, Zihan Huo, Shuai Ren, Linqi Hu, Jie Liu, Yanxia Gao, Jianguo Li

**Affiliations:** ^1^College of Animal Science and Technology, Hebei Agricultural University, Baoding, China; ^2^Key Laboratory of Healthy Breeding in Dairy Cattle (Co-construction by Ministry and Province), Ministry of Agriculture and Rural Affairs, Baoding, China; ^3^College of Veterinary Medicine, Hebei Agricultural University, Baoding, China; ^4^Cangzhou Normal University, College of Life Science, Cangzhou, China; ^5^Department of Animal and Poultry Production, Faculty of Agriculture, South Valley University, Qena, Egypt; ^6^Hebei Technology Innovation Center of Cattle and Sheep Embryo, Baoding, China; ^7^Hebei Research Institute of Dairy Industry Technology, Shijiazhuang, China

**Keywords:** rumen-degradable starch, rumen-degradable protein, lactation performance, nitrogen utilization efficiency, Holstein dairy cows

## Abstract

The dietary rumen-degradable starch (RDS) to rumen-degradable protein (RDP) ratio, denoted as the RDS-to-RDP ratio (SPR), has been proven to enhance *in vitro* rumen fermentation. However, the effects of dietary SPR *in vivo* remain largely unexplored. This study was conducted to investigate the effect of dietary SPR on lactation performance, nutrient digestibility, rumen fermentation patterns, blood indicators, and nitrogen (N) partitioning in mid-lactating Holstein cows. Seventy-two Holstein dairy cows were randomly assigned to three groups (24 head/group), balanced for (mean ± standard deviation) days in milk (116 ± 21.5), parity (2.1 ± 0.8), milk production (42 ± 2.1 kg/d), and body weight (705 ± 52.5 kg). The cows were fed diets with low (2.1, control), medium (2.3), or high (2.5) SPR, formulated to be isoenergetic, isonitrogenous, and iso-starch. The study consisted of a one-week adaptation phase followed by an eight-week experimental period. The results indicated that the high SPR group had a lower dry matter intake compared to the other groups (*p* < 0.05). A quadratic increase in milk yield and feed efficiency was observed with increasing dietary SPR (*p* < 0.05), peaking in the medium SPR group. The medium SPR group exhibited a lower milk somatic cell count and a higher blood total antioxidant capacity compared to other groups (*p* < 0.05). With increasing dietary SPR, there was a quadratic improvement (*p* < 0.05) in the total tract apparent digestibility of crude protein, ether extract, starch, neutral detergent fiber, and acid detergent fiber. Although no treatment effect was observed in rumen pH, the rumen total volatile fatty acids concentration and microbial crude protein synthesis increased quadratically (*p* < 0.05) as dietary SPR increased. The molar proportion of propionate linearly increased (*p* = 0.01), while branched-chain volatile fatty acids linearly decreased (*p* = 0.01) with increasing dietary SPR. The low SPR group (control) exhibited higher concentration of milk urea N, rumen ammonia N, and blood urea N than other groups (*p* < 0.05). Despite a linear decrease (*p* < 0.05) in the proportion of urinary N to N intake, increasing dietary SPR led to a quadratic increase (*p* = 0.01) in N utilization efficiency and a quadratic decrease (*p* < 0.05) in the proportion of fecal N to N intake. In conclusion, optimizing dietary SPR has the potential to enhance lactation performance and N utilization efficiency. Based on our findings, a medium dietary SPR (with SPR = 2.3) is recommended for mid-lactating Holstein dairy cows. Nevertheless, further research on rumen microbial composition and metabolites is warranted to elucidate the underlying mechanisms of the observed effects.

## 1 Introduction

Ruminants rely critically on rumen microbes to digest plant feed (1). The rumen microbial activity is closely linked to feed efficiency (2), and their microbial crude protein (MCP) supplies over half of the metabolizable protein reaching the small intestine for ruminants (3). Synchronizing the supply of energy and nitrogen (N) in the rumen was suggested as one effective strategy to maximize the capture of rumen degradable protein (RDP) and enhance rumen microbial activity and growth (4). The effects of synchronous diets have been widely explored *in vitro* and *in vivo* studies (5). However, the results were reported to be inconsistent. One potential reason for this discrepancy is the failure to accurately match the form of energy carrier substances. While current research has considered the degradation characteristics of dietary proteins in the rumen, the energy evaluation system for ruminant feed predominantly relies on chemical analysis, which does not accurately reflect the energy supply within the rumen (6).

The availability of carbohydrates serves as the primary factor controlling the energy supply for rumen microbes (7). These carbohydrates were categorized into four fractions (8): neutral detergent fiber (NDF), starch, neutral detergent soluble fiber (NDSF), and water-soluble carbohydrates (WSC). Compared to NDF, starch exhibits a faster fermentation rate in the rumen. This made the supply of energy at a rate closer to the ammonia released, thereby promoting rumen microbial activity and growth (5). The rumen-degradable starch (RDS), representing the extent and rate of starch degradation in the rumen, had been reported to be more effective than rumen-degradable NDF in promoting MCP synthesis (9). Additionally, in comparison to NDSF (e.g., pectin) and WSC (e.g., sucrose), starch could provide more carbon skeletons for rumen microbes (10). The starch-based diet led to more MCP synthesis *in vitro* culture (10) and milk protein yield in dairy cows (11) compared to a sucrose- (or pectin-) based diet. Therefore, the starch, especially RDS, might be more effective in regulating the rumen microbial growth and fermentation than other carbohydrates. Furthermore, the RDP function as the primary N source for rumen microbes, significantly influencing the composition of rumen microbes (12) and MCP synthesis (13). Davies et al. (14) reported that a judicious combination of RDS and RDP in low crude protein (CP) diets had the potential to improve MCP synthesis efficiency and animal productivity. Martins et al. (15) suggested that dietary recommendations for RDP should consider RDS for a more precise level to enhance MCP synthesis and minimize N excretion. Considering these study results, the dietary RDS to RDP ratio (SPR) might be an effective indicator of optimizing rumen microbial growth and rumen fermentation.

Our previous *in vitro* study showed a quadratic response in MCP synthesis and total volatile fatty acids (TVFA) concentration with increasing dietary SPR (16). However, the effect of dietary SPR *in vivo* has yet to be entirely determined. Therefore, we hypothesize that dietary SPR has the potential to optimize rumen microbial growth and fermentation, subsequently modulating lactation performance and nitrogen use efficiency (NUE) in dairy cows. Our objective was to evaluate the effects of dietary SPR on lactation performance, nutrient digestibility, rumen fermentation patterns, and N partitioning in mid-lactating Holstein cows.

## 2 Materials and methods

### 2.1 Ethics statement

This study was conducted between March 2021 and June 2021 at Hongda Commercial Dairy Farm in Baoding, China. The experiment was approved by the Institutional of Animal Care and Use Committee at Hebei Agricultural University, Baoding, China (with protocol JGL 2103; approval date: March 1, 2021). The Hongda Commercial Dairy Farm provided the necessary approvals and cooperation for the research.

### 2.2 Animals and experimental design

Seventy-two Holstein dairy cows (24 head/group) were used in a complete randomized design. Cows balanced for (mean ± standard deviation) days in milk (116 ± 21.5), parity (2.1 ± 0.8), milk production (42 ± 2.1 kg/d), and body weight (705 ± 52.5 kg) were assigned to one of three treatments. Three distinct diets were formulated with different SPR levels, which were low SPR (L-SPR, RDS/RDP = 2.1), medium SPR (M-SPR, RDS/RDP = 2.3), or high SPR (H-SPR, RDS/RDP = 2.5), respectively. The L-SPR treatment was designed as the control group based on the observed dietary SPR value in farms exhibiting low NUE. The M-SPR treatment was derived from the optimal rumen fermentation performance observed in previous *in vitro* experiment (16). The H-SPR treatment was derived from earlier studies that integrated the maximum levels of RDS without observing adverse effects on rumen function (17). Cows were individually housed in tie stalls equipped with automatic drinking bowls. Cows had free access to water and were fed a total mixed ration (TMR; Table 1) twice daily at 0800 and 1,600 h. Cows were fed *ad libitum* throughout the study, ensuring at least 5% feed refusals. Cows were milked thrice daily at 0730, 1530, and 2,300 h. The study was conducted with 1-week to adapt to the experimental conditions, followed by an 8-week for sampling and data collection.

**Table 1:** Ingredients of experimental diet fed as a TMR (% of DM).

Ingredients, % of DM	Treatments (SPR)^1^		
	L-SPR (Control)	M-SPR	H-SPR
Corn silage	31.4	31.5	31.3
Alfalfa hay	4.93	4.94	4.92
Oat hay	12.4	12.4	12.4
Ground corn	15.7	7.9	0.9
Ground wheat	4.0	11.9	19.6
Solvent-extract soybean meal	11.6	8.3	5.7
Heat-treated soybean meal^2^	1.37	3.34	5.09
Wheat bran	2.16	2.95	2.55
Beet pellets	11.1	11.5	12.1
Fat powder	2.36	2.36	2.43
Premix^3^	3.0	3.0	3.0

^1^SPR, dietary rumen-degradable starch to rumen-degradable protein ratio; L-SPR, low SPR; M-SPR, medium SPR; H-SPR, high SPR.^2^Xingpu Feed Co. LTD, Harbin, China, the intestinal digestibility of rumen-undegradable protein was 75.2%.^3^Premix contained (per kg of DM): 2600 mg of NaHCO_3_, 2,300 mg of CaHPO_4_, 1,300 mg of CaCO_3_, 700 mg of NaCl, 650 mg of Zn, 500 mg of Mn, 350 mg of MgO, 350 mg of Fe, 180 mg of Cu, 6 mg of Co, 8 mg of Se, 10 mg of I, 240,000 IU of vitamin A, 53,000 IU of vitamin D_3_, 2,000 mg of vitamin E, 13 mg of biotin, and 90 mg of β-carotene.

### 2.3 Diets and feed ingredients

The experimental diets were formulated to meet the recommendation of NRC (2001) (18) using the AMTS software platform (Agricultural Modeling & Training Systems, Groton, NY, United States) for a 700 kg cow producing 40 kg/d of milk containing 4.0% fat and 3.0% true protein. The diets contained 49% forage (forage sources and proportions were consistent across all diets) and 51% concentrate on a dry matter (DM) basis. All forages and concentrates were weighed and mixed thoroughly each morning using a mixer wagon (9SJW-500, Goke Agriculture Machinary Co. Ltd., Beijing, China). Diets maintained consistent levels of net energy for milk, CP, starch, neutral detergent fiber (NDF), and acid detergent fiber (ADF; Table 2). Differences in levels of RDS and RDP arose from varying concentrate mixtures. Specifically, RDS levels were adjusted by varying the proportions of ground corn and ground wheat in the diet, and the proportions of solvent-extract soybean meal (SSBM) and heat-treated soybean meal (HSBM) were regulated to maintain a consistent RDP level. A single source of wheat and corn was processed using a roller mill (model SSLG-15, Shuanghe Machinery Manufacturing Co. LTD, Shandong, China) to obtain ground wheat and ground corn. The particle size analysis, performed by the ASAE (2003, method S319.3) (19), revealed that the geometric mean particle sizes were 845 μm for ground wheat and 829 μm for ground corn, respectively. The HSBM used in this study were commercially manufactured using the same production process and batch (Xingpu Feed Co. LTD, Harbin, China). The *in situ* residues of SSBM and HSBM after 16 h of ruminal incubation were employed to determine the small intestinal digestibility of rumen-undegraded protein (20), with respective digestibility of 82.7 and 75.2%.

**Table 2:** Chemical composition of experimental diet fed as a TMR (% of DM, unless noted).

Composition	Treatments (SPR)^1^		
	L-SPR (Control)	M-SPR	H-SPR
NE_L,_ Mcal / kg^2^	1.7	1.7	1.7
CP	15.6	15.6	15.7
RDP^3^	9.3	9.3	9.3
Starch	26.4	26.5	26.7
RDS^3^	19.4	21.1	22.9
SPR	2.1	2.3	2.5
Ether extract	4.5	4.6	4.8
NDF	31.6	31.7	31.7
ADF	17.5	17.6	17.7
Forage NDF	20.3	20.4	20.3
peNDF_8.0_^4^	15.2	15.2	15.3
ERDST^5^	73.5	79.6	85.9
ERDCP^5^	59.6	59.6	59.2

^1^SPR, dietary rumen-degradable starch to rumen-degradable protein ratio; L-SPR, low SPR; M-SPR, medium SPR; H-SPR, high SPR.^2^NE_L_, net energy for lactation, estimated by NRC (2001).^3^RDP (rumen degradable protein, %) = CP (%) × ERDCP (%); RDS (rumen degradable starch, %) = Starch (%) × ERDST (%).^4^peNDF_8.0_, physically effective NDF inclusive of particles > 8 mm, measured as the NDF content of diet multiplied by pef_8.0_.^5^ERDS, effective ruminal degradability for starch; ERDCP, effective ruminal degradability for protein.

The dietary SPR was calculated as the ratio of dietary RDS to RDP. The dietary RDS or RDP content was calculated based on the actual measured values of the ingredients applied in the formulation, as determined by Equations (1,2) (21):


(1) $$RDS=\overset{n}{\underset{i=1}{\sum }}{PST}_{i}\times {\mathrm{ERDST}}_{i}$$

(2) $$RDP=\overset{n}{\underset{i=1}{\sum }}{PCP}_{i}\times {\mathrm{ERDCP}}_{i}$$

Where n is the number of ingredients containing starch or CP in the diet; PST_i_ represents the dietary starch proportion of feed i in the diet; PCP_i_ represents the dietary CP proportion of feed i in the diet, ERDST_i_ represents the effective rumen starch degradability of feed i; ERDCP_i_ represents the effective rumen CP degradability of feed i.

The ERDST and ERDCP in the feed ingredients employed in this study were assessed through the *in situ* nylon bags technique. The nylon bags with a pore size of 50 μm were used, and the rumen outflow rate was set at 0.06/h according to Offner et al. (6). A detailed method of *in situ* ruminal degradation was referred to Li et al. (22). Degradation parameters and effective ruminal degradability (ERD) were determined using Equations (3,4) from Ørskov and McDonald (23):


(3) $$Y_{t}=a+b\times \left(1-e^{-kt}\right)$$

(4) $$ERD=a+b\times k/\left(k+k_{p}\right)$$

where Y_t_ = disappearance proportion at time t; a = rapidly degradable fraction; b = slowly degradable fraction; k = constant rate of degradation of fraction b; t = time of incubation (h); kp = passage rate, the rumen outflow rate was set at 0.06/h. The nutrient content, ERDCP, and ERDST of the primary ingredients used in this study are listed in Supplementary Table S1.

### 2.4 Sampling and data collection

#### 2.4.1 Collection of feed samples

Feed offered and refused were recorded daily. The TMR, feed ingredients, and feed refusals were collected weekly. The collected samples were immediately oven-dried at 55°C for 48 h to measure DM content. Subsequently, the samples were ground using a 1-mm screen (stand model 4 Wiley Mill, Arthur H. Thomas, Philadelphia, PA, United States) for chemical analyses. Daily DMI was calculated by subtracting the DM refusals from the DM offered. Weekly averages of DMI data were used for statistical analysis (24).

#### 2.4.2 Collection of milk samples

Milk yield was recorded daily using the DeLaval milking system at milking time, and those records were used to calculate weekly averages for each cow for statistical analysis. Milk samples were obtained on d 1 weekly by mixing proportional aliquots from each milking and immediately transported to the laboratory for analysis. According to International Dairy Federation (25), the fat- and protein-corrected milk (FPCM) yield was computed as milk yield (kg/d) × (0.1226 × fat % + 0.0776 × protein % + 0.2534). Feed efficiency was determined as FPCM yield divided by DMI. The NUE was calculated as milk N divided by N intake.

#### 2.4.3 Collection of feces and urine samples

Due to animal welfare, eight cows from each treatment (balanced for parity, milk production, and days in milk) were selected for feces, urine, rumen fluid, and blood sampling. Spot feces and urine samples were collected from each cow during weeks 0, 4, and 8. The samples cover 3 days (0200, 1100, and 2000 h on d 2; 0500, 1400, and 2300 h on d 3; 0800 and 1700 h on d 4 of each sampling week), representing 3, 6, 9, 12, 15, 18, 21, and 24 h after morning feeding. Approximately 100 g of fresh feces were collected from the rectum, pooled by each cow, dried at 55°C for 48 h, and ground through a 1-mm screen (stand model 4 Wiley Mill; Arthur H. Thomas, Philadelphia, PA, United States). Urine was collected by stimulating the vulva. Subsequently, the urine samples were acidified using a 4:1 volumetric ratio of 0.072 mol/L H_2_SO_4_ to urine. The acidified samples were then frozen at −20°C for further analysis.

#### 2.4.4 Collection of rumen fluid samples

Rumen fluid samples were collected using an oral stomach tube during week 0, 4, and 8 (26). Five sampling times over 3 d were selected (1100 and 2000 h on d 5; 0800 and 1700 h on d 6; 1400 h on d 7 of each sampling week), representing 0, 3, 6, 9, and 12 h after morning feeding. To secure representative rumen samples, the oral stomach tube was inserted approximately 200 cm deep, reaching the central rumen. Additionally, to mitigate the influence of extraneous factors, rumen fluid sampling was consistently carried out by a single individual. The initial 100 mL of rumen fluid was discarded to prevent contamination from saliva or mucus. Subsequently, another 100 mL of rumen fluid was collected and filtered through four layers of cheesecloth. The rumen liquid pH was immediately measured using a portable pH meter (DENVER UB-7, Denver Instrument, Denver, United States). Two subsamples (5 mL) of rumen fluid were, respectively, preserved with 1 mL of 25% (wt/vol) HPO_3_ and 1 mL of 1% (wt/vol) H_2_SO4 and stored at −20°C until the determination of VFAs and ammonia N (NH_3_-N).

#### 2.4.5 Collection of blood samples

Blood samples were collected from the coccygeal vein before morning feeding on d 7 of week 0, 4, and 8. Approximately 20 mL of blood samples were collected into two 10 mL vacuum tubes (Vacutainer, Becton Dickinson, Franklin Lakes, NJ, United States) with no additives. Then, according to Shen et al. (27), serum samples were prepared and stored at −20°C for later analyses.

#### 2.4.6 Measurement of body weight

Cows were weighed before morning feeding on the last day of week 0, 4, 8. Body weights were adjusted by subtracting the morning milking weights to accurately represent the cows’ actual weight.

### 2.5 Samples analysis

#### 2.5.1 Analysis of feed and fecal samples

The DM (method 930.15), ash (method 942.05), CP (method 968.06), ether extract (EE, method 920.39), and starch (method 996.11) contents in TMR, feed refusals, and feces were determined according to AOAC (28). The organic matter (OM) content was calculated as the difference between 100% and ash content. The content of NDF and ADF in TMR, feed refusals, and feces were analyzed using heat-stable α-amylase and sodium sulfite (29) and were expressed inclusive of residual ash (30). Acid detergent insoluble ash (ADIA) content in TMR, feed refusals, and feces was determined as outlined in Keulen and Young (31). The ADIA was used for calculating the apparent total-tract digestibility of nutrients (32), and the equation was:100 − (100 × (%ADIA in DM consumed/%ADIA in feces) × (%nutrient in feces/%nutrient in consumed DM)).

#### 2.5.2 Analysis of urine samples

The weight of cows and the concentration of creatinine in urine were used to estimate total urine production. The estimation formula is as follows: Urine volume (L/d) = (Body weight (kg) × Creatinine excretion rate (mg/kg)) / Urine creatinine concentration (mg/L). The creatinine excretion rate referred to the amount of creatinine excreted per kilogram of body weight per day by cows, with a value of 29 mg/kg (33). The urinary allantoin and uric acid excretions were calculated by multiplying the concentrations by the respective daily urine volume. Daily urinary purine derivatives (PD) excretion was the sum of allantoin and uric acid excretion. The PD was used as an indicator of MCP synthesis based on the relationship derived by Chen and Gomes (34). Urinary urea-N, creatinine, allantoin, and uric acid concentrations were determined using commercial kits from Nanjing Jiancheng Bioengineering Institute, Nanjing, China. The inter-assay coefficients of variation were less than 10%, and the intra-assay coefficients of variation were less than 12%. Allantoin in urine was determined by the colorimetric method (34). Urinary N was determined according to AOAC (method 968.06) (28).

#### 2.5.3 Analysis of milk samples

The percentage of milk fat, milk protein, lactose, milk urea nitrogen concentration (MUN), and somatic cell count (SCC) were determined using FOSS Milko Scan and Fossomatic FC (FOSS Food Technology Corp., Hillerød, Denmark). Yield of milk components was calculated by multiplying the daily milk yield by the percentage of a given milk component.

#### 2.5.4 Analysis of rumen fluid samples

The concentration of rumen VFA was determined using gas chromatography (Agilent, 7890A, a fused silica column, 30 m × 0.32 mm × 0.25 mm; column temperature, 150°C; injector temperature, 200°C; and detector temperature, 250°C) as described by Shen et al. (35). The rumen fluid concentration of NH_3_-N was measured using a phenol-hypochlorite assay (36).

#### 2.5.5 Analysis of blood samples

Commercial kits (Jiancheng Bioengineering Inc., Nanjing, China) were used to determine the concentration of blood urea nitrogen (BUN) and glucose, as well as the activities of aspartate aminotransferase (AST) and alanine aminotransferase (ALT). Commercial ELISA kits (Huayuechang Biotechnology Co., Ltd., Beijing, China) were used to determine the total antioxidant capacity (T-AOC, kit no: DRE98021, detection range: 0–8 U/mL), insulin capacity (kit no: DRE98105, detection range: 0.8–30 mU/L), the insulin-like growth factor-1 concentration (IGF-1, kit no: DRE98016, detection range: 10–300 μg/L) and the nonesterified fatty acid concentration (NEFA, kit no: DRE98327, detection range: 20–560 μmol/L). The inter-assay coefficients of variation were lower than 10%, and the intra-assay coefficients of variation were lower than 12%.

### 2.6 Calculations and statistical analysis

Before analyses, all data were tested for normality using the Shapiro–Wilk test, and any parameter that was not normally distributed was normalized by Box-Cox transformation. The data were analyzed using the PROC MIXED procedure of SAS 9.4 (SAS Inst. Inc., Cary, NC). The statistical model was as follows:


$$Y_{ijk}=\mu +T_{i}+D_{j\left(i\right)}+{\mathrm{cov}}_{j}+E_{ijk}$$

where Y_ijk_ is the dependent variable; *μ* is the overall mean; *i* is the SPR treatment, *j* is the cow; *T_i_* is the effect of *i*th SPR treatment; *D_j (i)_* is the random effect of the *j*th cow within SPR treatment; *Cov_j_* is the covariate effect, the data from week 0 were used for the covariate analysis; E_ijk_ is the residual error.

The model included diet as fixed effects, cow within SPR treatment, and residual error as random effects. The repeated measures analysis of results was subjected to five models (AR, UN, CS, SP, and VC). The weeks of data collection served as repeated measurements for DMI, milk production, milk composition, and feed efficiency. The days of sample collection function as repeated measurements for apparent nutrient digestibility, rumen fermentation parameters, nitrogen partitioning indicators, and blood indicators. The covariance structure with the smallest Schwarz-Bayesian criterion was used owing to the most desirable and reliable analysis (37). The PDIFF option of SAS was used to calculate and separate the least squares means. Orthogonal contrasts were conducted to determine linear and quadratic dose of dietary SPR responses. Treatment effects were declared significant at *p* < 0.05.

## 3 Results

### 3.1 Dry matter intake and milk performance

**Figure 1:**
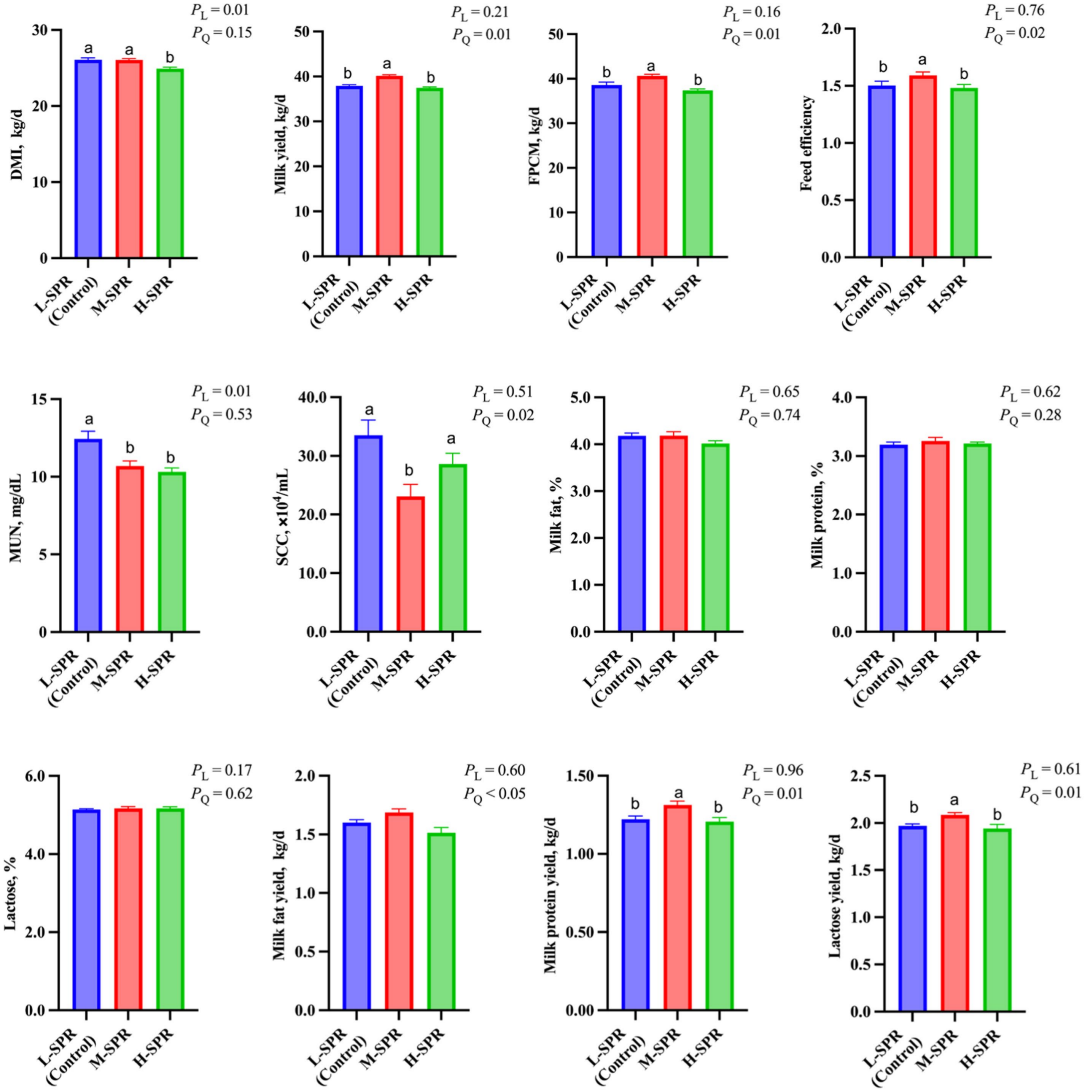
Effect of dietary rumen-degradable starch to rumen-degradable protein ratio (SPR) on dry matter intake and lactation performance in mid-lactating Holstein cows. Error bars indicate measure of variation within the dietary SPRs. Different letters (a–b) indicate statistically significant difference (*p* < 0.05). L is linear, and Q is quadratic effects for diet SPR; DMI, dry matter intake; MUN, milk urea nitrogen; FPCM, Fat- and protein-corrected milk; Feed efficiency, FPCM/DMI.

With increasing dietary SPR, there was a linear decrease in DMI (*p* = 0.01; Figure 1). The H-SPR group was reduced by 0.8 kg/d compared to the L-SPR group (control) and by 0.7 kg/d compared to the M-SPR group. Milk yield (*p* = 0.01), FPCM yield (*p* = 0.01), and feed efficiency (*p* = 0.02) exhibited a quadratic increase, with the M-SPR group demonstrating a 4.19% increase in milk yield, 4.63% in FPCM production, and 4.67% in feed efficiency compared to the L-SPR group (control). The SCC demonstrated a quadratic decrease as the dietary SPR increased (*p* = 0.02), with the M-SPR group showing a decrease of 31.04% compared to the L-SPR group (control). Different dietary SPRs did not influence the concentrations of milk fat, protein, and lactose, but the production of these milk components exhibited a quadratic increase (*p* < 0.05). Furthermore, in comparison to the L-SPR group (control), the concentrations of MUN in the M-SPR and H-SPR groups showed a linear decrease (*p* < 0.05), decreasing by 24.4 and 26.9%, respectively.

### 3.2 Total-tract apparent nutrient digestibility

**Figure 2:**
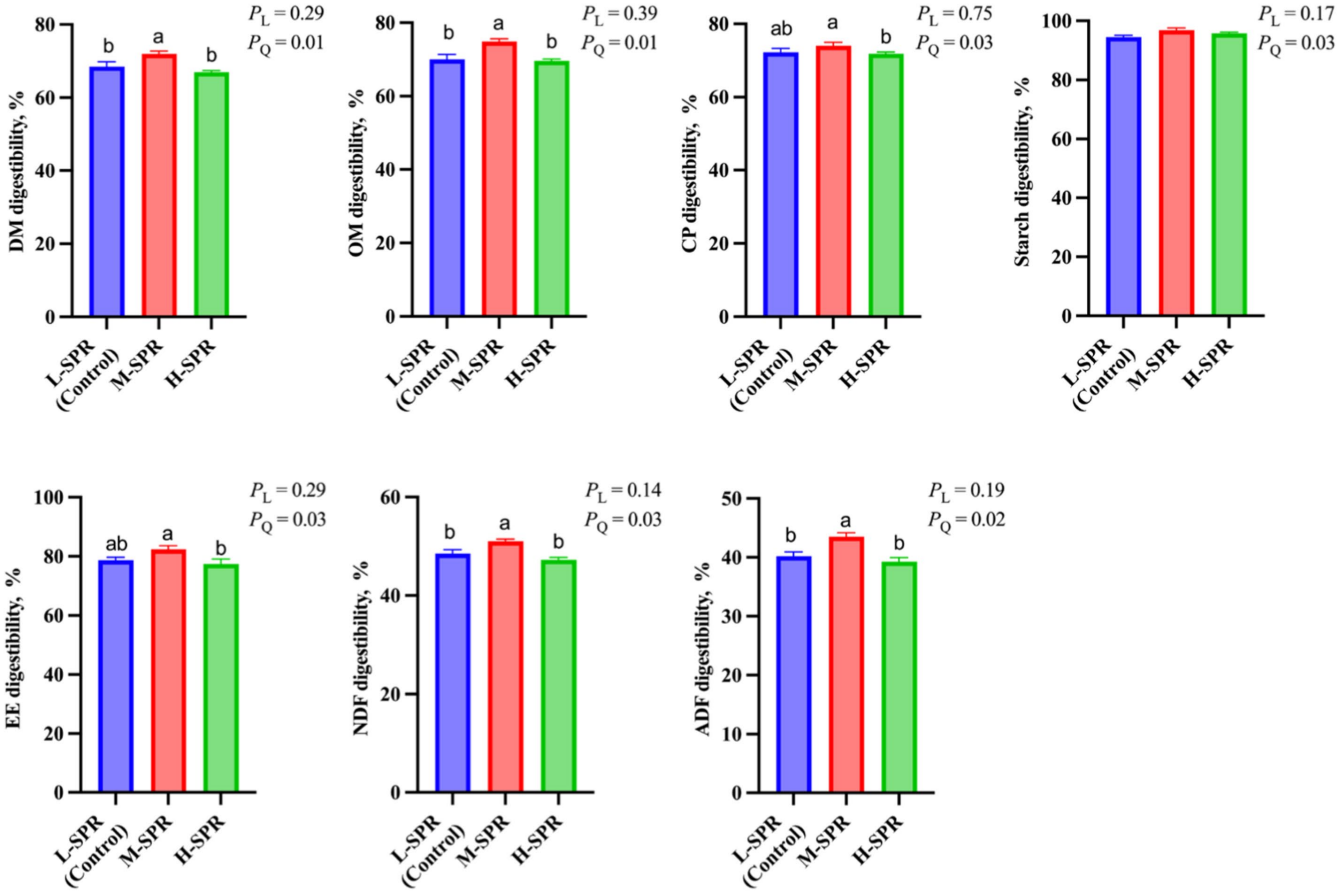
Effect of dietary rumen-degradable starch to rumen-degradable protein ratio (SPR) on nutrient apparent digestibility in mid-lactating Holstein cows. Error bars indicate measure of variation within the dietary SPRs. Different letters (a–b) indicate statistically significant difference (*p* < 0.05). L is linear, and Q is quadratic effects for diet SPR; DM, dry matter; OM, organic matter; CP, crude protein; EE, ether extract; NDF, neutral detergent fiber; ADF, acid detergent fiber.

As shown in Figure 2, the digestibility of DM (*p* = 0.01), OM (*p* = 0.01), CP (*p* = 0.03), EE (*p* = 0.03), starch (*p* = 0.03), NDF (*p* = 0.03), and ADF (*p* = 0.02) exhibited a quadratic increase with increasing dietary SPR, peaking in the M-SPR group. Compared to the L-SPR group (control), the M-SPR group showed significant improvements in DM (4.81%), OM (5.64%), NDF (4.29%), and ADF (6.14%) digestibility.

### 3.3 Rumen fermentation patterns

**Figure 3:**
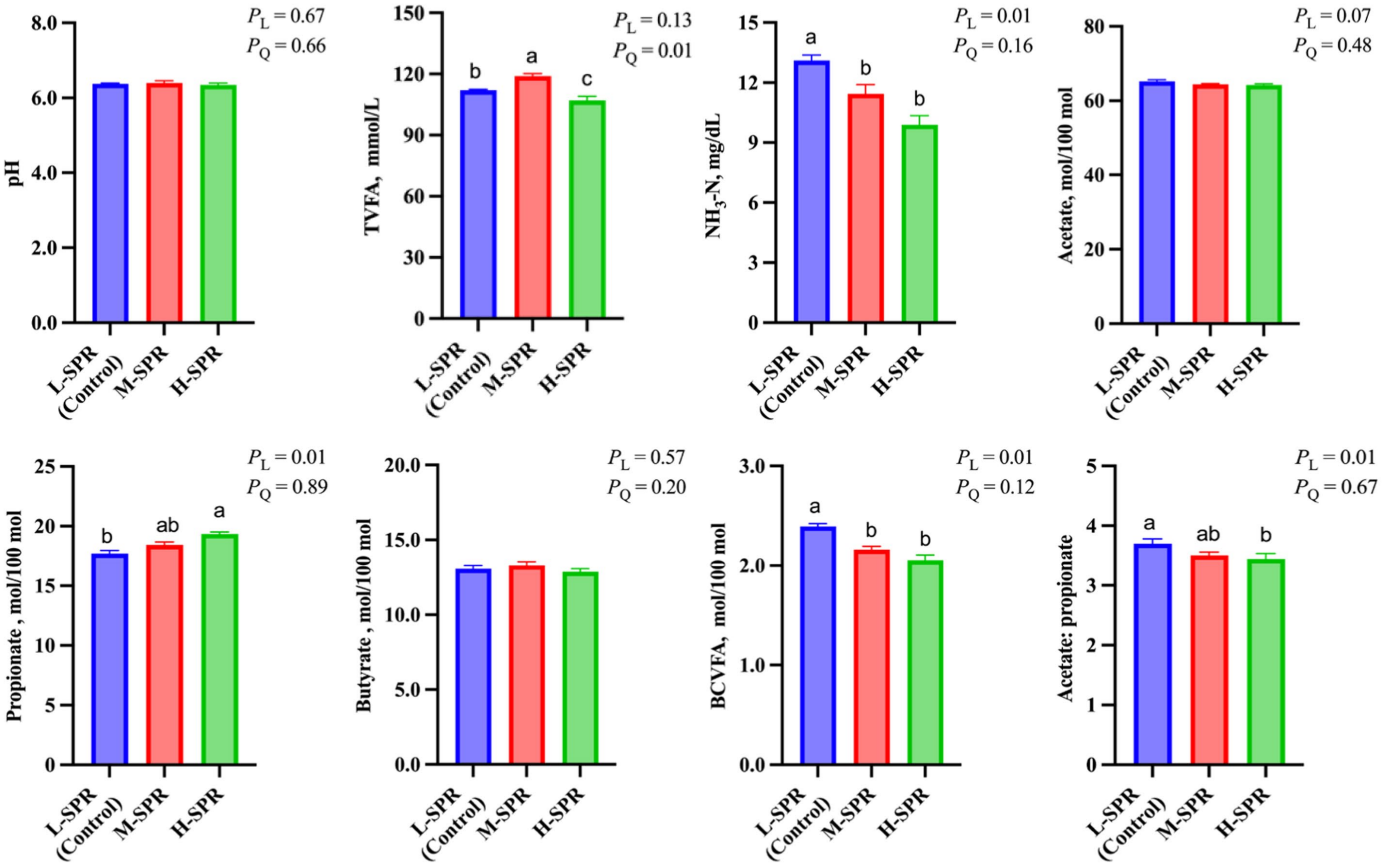
Effect of dietary SPR on rumen fermentation patterns in mid-lactating Holstein cows. Error bars indicate measure of variation within the dietary SPRs. Different letters (a–c) indicate statistically significant difference (*p* < 0.05). L is linear, and Q is quadratic effects for diet SPR. TVFA, total volatile fatty acid; Branched-chain volatile fatty acid = isobutyrate + isovalerate; NH_3_-N, ammonia nitrogen.

According to Figure 3, the dietary SPR did not affect rumen pH. However, as the dietary SPR increased, there was a quadratic increase in TVFA concentration (*p* = 0.01), with the M-SPR group showing a 5.36% rise compared to the L-SPR group (control). Although the molar proportion of acetate was not affected by dietary SPR, the molar proportion of propionate linearly increased (*p* = 0.01), and the ratio of acetate to propionate linearly decreased (*p* = 0.01) with increasing dietary SPR. Additionally, in comparison to the L-SPR group (control), the concentrations of NH_3_-N in the M-SPR and H-SPR groups showed a linear decrease (*p* = 0.01), decreasing by 17.5 and 27.7%, respectively.

### 3.4 Microbial crude protein synthesis

**Figure 4:**
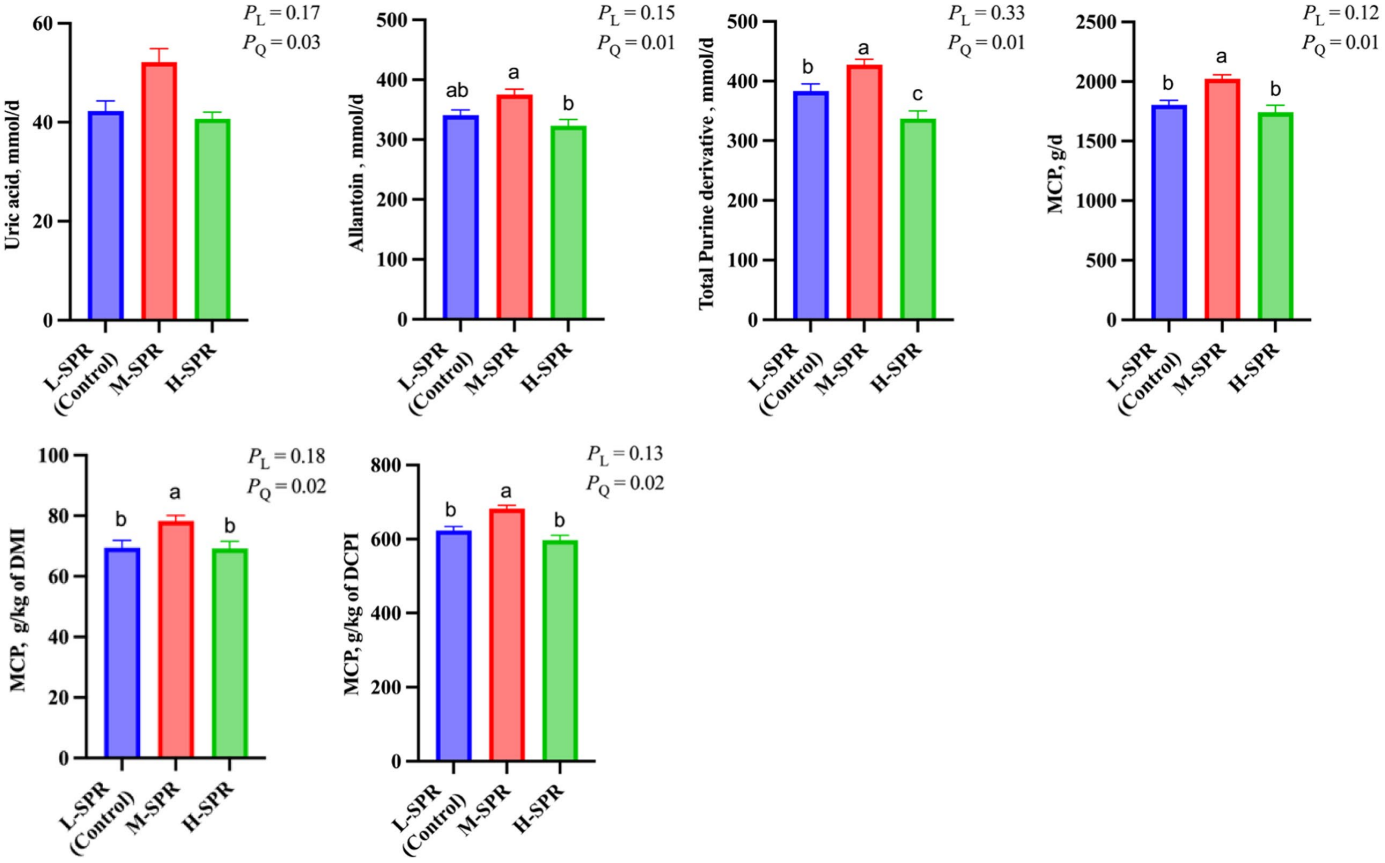
Effect of dietary rumen-degradable starch to rumen-degradable protein ratio (SPR) on urinary purine derivatives excretion in mid-lactating Holstein cows. Error bars indicate measure of variation within the dietary SPRs. Different letters (a–c) indicate statistically significant difference (*p* < 0.05). L is linear, and Q is quadratic effects for diet SPR. MCP, microbial crude protein; DMI, dry matter intake; DCPI, digestible CP intake.

In Figure 4, there was a quadratic response in the uric acid (*p* = 0.03), allantoin (*p* = 0.01), and total PD (*p* = 0.01) excretion, as dietary SPR increased, with the greatest amount reached at the M-SPR group. Accordingly, a quadratic increase (*p* < 0.05) was observed in MCP synthesis (g/d; g/kg of DMI; g/kg of digestible CP intake) with increasing dietary SPR. Compared to the L-SPR group (control), the M-SPR group exhibited a 9.16% increase in MCP (g/d), a 10.56% increase in MCP (g/kg of DMI), and a 9.02% increase in MCP (g/kg of digestible CP intake).

### 3.5 Blood indicators

**Figure 5:**
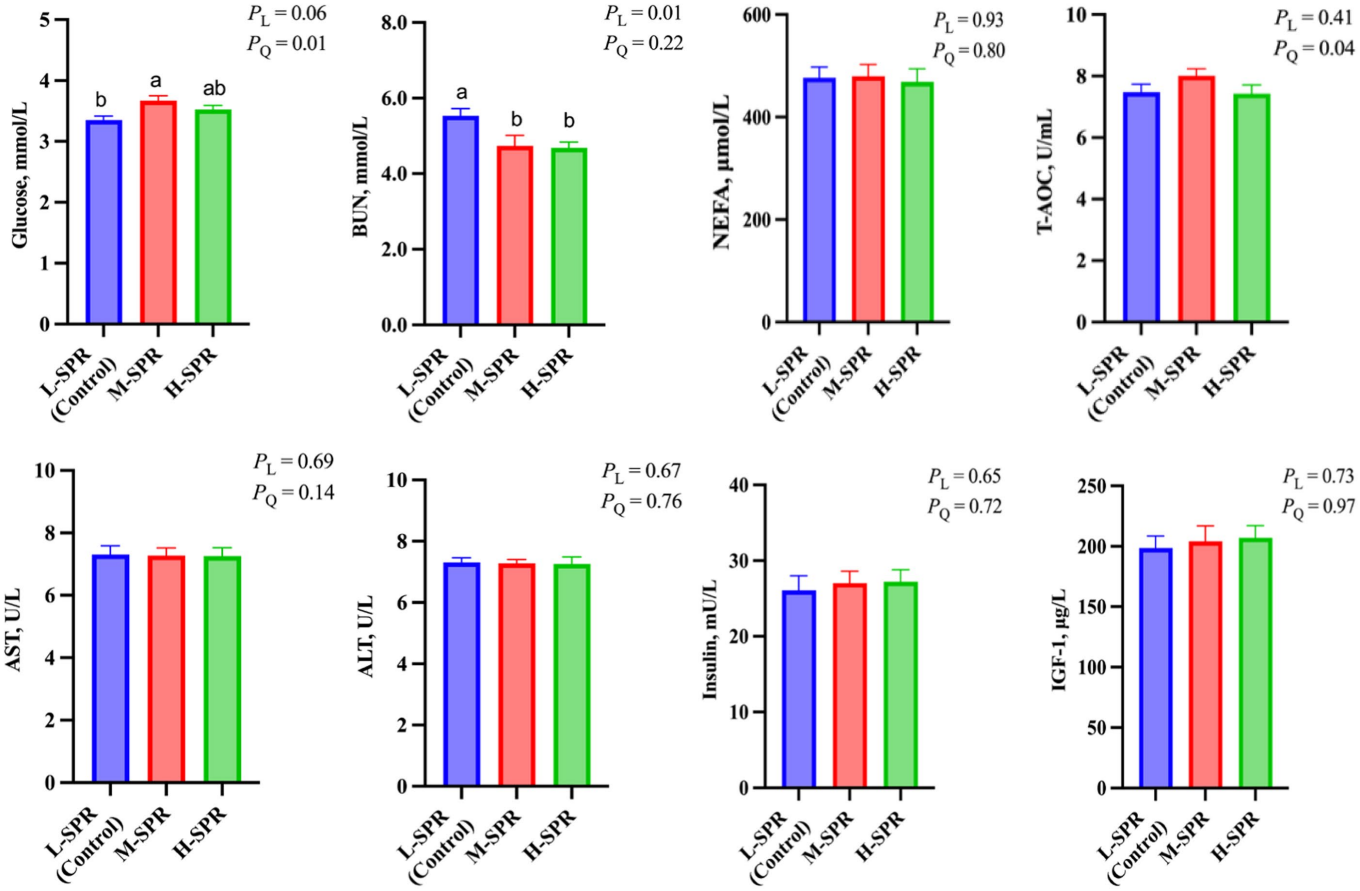
Effect of dietary rumen-degradable starch to rumen-degradable protein ratio (SPR) on blood indicators in mid-lactating Holstein cows. Error bars indicate measure of variation within the dietary SPRs. Different letters (a–b) indicate statistically significant difference (*p* < 0.05). L is linear, and Q is quadratic effects for diet SPR; BUN, blood urea nitrogen; NEFA, nonesterified fatty acid; T-AOC, total antioxidant capacity; AST, aspartate aminotransferase; ALT, alanine aminotransferase; IGF-1, insulin-like growth factor-1.

As depicted in Figure 5, increasing the dietary SPR led to a quadratic rise in GLU concentration (*p* = 0.01), with the M-SPR group showing a significant 9.55% increase compared to the L-SPR group (control). The concentration of BUN displayed a linear decrease (*p* < 0.05), with reductions of 13.56 and 15.37% in the M-SPR and H-SPR groups, respectively, as compared to the L-SPR group (control). The T-AOC activity showed a quadratic rise with the increase of dietary SPR (*p* = 0.04), and the M-SPR group showed a notable 6.79% improvement compared to the L-SPR group (control). Furthermore, there were no significant differences among the three groups in the activity of AST, ALT, and insulin, as well as the concentration of NEFA and IGF-1.

### 3.6 Nitrogen partitioning

**Figure 6:**
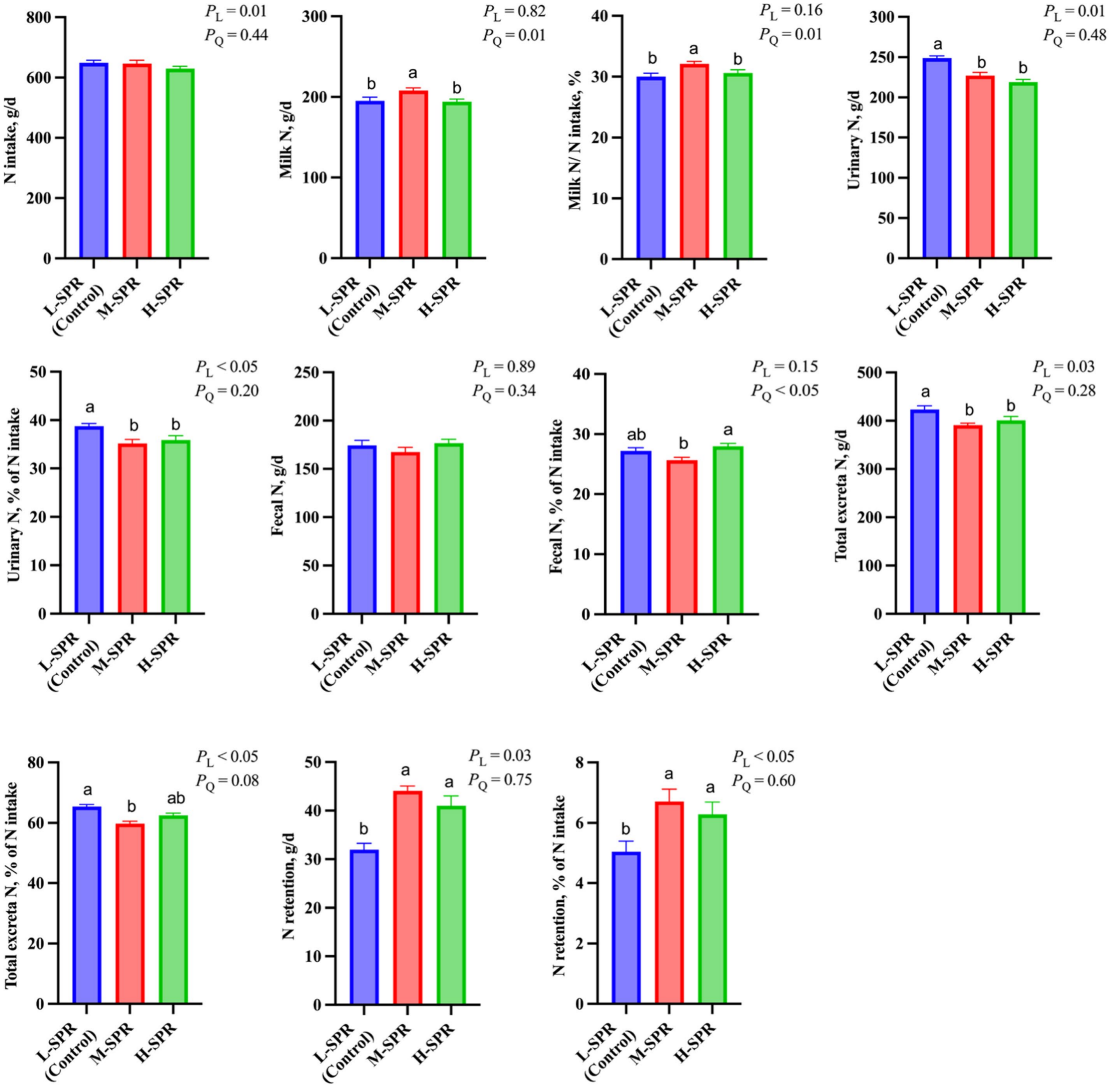
Effect of dietary rumen-degradable starch to rumen-degradable protein ratio (SPR) on nitrogen partitioning in mid-lactating Holstein cows. Error bars indicate measure of variation within the dietary SPRs. Different letters (a–b) indicate statistically significant difference (*p* < 0.05). L is linear, and Q is quadratic effects for diet SPR; Milk N, milk crude protein ÷ 6.25; Total N excretion, fecal N + urinary N; N retention, N intake – milk N – urinary N – fecal N.

Although the N intake linearly decreased (*p* = 0.01; Figure 6), the milk N secretion (*p* = 0.01) and the proportion of milk N to N intake (*p* = 0.01) quadratically increased with increasing dietary SPR. Compared to the L-SPR group (control), the M-SPR group demonstrated a 6.47% increase in milk N secretion and a 7.00% increase in the proportion of milk N to N intake. With increasing dietary SPR, the proportion of fecal N to N intake exhibited a quadratic decrease (*p* < 0.05). Additionally, urinary N excretion (*p* = 0.01) and the proportion of urinary N to N intake (*p* < 0.05) showed a linear decrease. The N retention (*p* = 0.03) and the proportion of N retention to N intake (*p* < 0.05) displayed a linear increase. Total excretion N exhibited a quadratic decrease with the increase in dietary SPR (*p* < 0.05), resulting in a 6.15% reduction in the M-SPR group compared to the L-SPR group (control).

## 4 Discussion

In the previous *in vitro* experiment, dietary SPR had been established as an effective indicator for regulating rumen fermentation and MCP synthesis (16). Analyses of diets and milk from large-scale farms revealed a notable increase in MUN content when the dietary SPR fell below 2.1, which corresponded to the SPR value designated for the control group in this study. This increase in MUN was likely due to the surplus NH_3_-N in the rumen, stemming from deficiencies in fermentable carbohydrates or excessive protein degradation (38). Therefore, optimizing dietary SPR by adjusting RDS or RDP levels could reduce rumen NH_3_-N wastage, ultimately enhancing lactation performance. While current feeding standards recommend dietary RDP levels, less attention has been given to dietary RDS levels, which influence rumen energy and carbon skeleton supply (9, 10). This study maintained a consistent dietary RDP level while adjusting dietary SPR by altering RDS levels.

Although the diets were designed to have similar forage NDF and energy content, our study observed a linear decrease in DMI with increasing dietary SPR. Allen et al. (39) point out that the regulation of DMI is primarily achieved through metabolic signals rather than rumen fill effects. Miyaji et al. (40) and Savari et al. (41) reported that increasing RDS level could increase propionate production, leading to a lower DMI, which was consistent with our study. In the present study, the dietary RDS level elevated with increasing dietary SPR, which support more starch to be fermented in the rumen for more propionate release. In the liver, propionate is utilized for gluconeogenesis while also stimulating the oxidation of acetyl-CoA (42). In this process, acetyl-CoA is used for energy supply to increase ATP production, enhancing satiety, and stopping feed intake. Thus, the linear decrease in DMI might be explained by the linear increase in propionate proportion.

Cows in the M-SPR group produced 1.6 kg more milk compared to those in the L-SPR group (control), despite similar numerical values for DMI, which aligns with findings by Santos et al. (43). The improved digestibility of OM in the M-SPR group might elevate glucose concentration, potentially resulting in higher lactose yield. Lactose plays a pivotal role in regulating milk osmotic pressure and production (44). Therefore, the enhancement of lactose yield might positively influence milk production. Moreover, the M-SPR group exhibited enhanced serum T-AOC capacity and decreased milk SCC, indicating improved antioxidant capacity and reduced milk losses (45). Hence, the enhancement in antioxidant status is another factor contributing to the increase in milk production.

Milk fat is a critical indicator for assessing the production performance of dairy cows. Zheng et al. (17) discovered that elevating dietary RDS level in goat led to a reduction in *de novo* fatty acid synthesis and milk fat production. In our study, the M-SPR group exhibited a significant improvement in the digestibility of NDF and ADF, potentially providing more acetate as precursors for milk fat synthesis. Additionally, the M-SPR group had a higher apparent digestibility of EE, allowing them to acquire more exogenous fatty acids, which is another reason for the increased milk fat yield. A meta-analysis by Ferraretto et al. (46) observed that a one-unit increase in RDS resulted in a corresponding 0.02-unit increase in milk protein content. Zhong et al. (47) reported a quadratic relationship in milk protein content and yields with the increasing proportion of rumen-fermentable carbohydrates. The MCP, comprising over half of the metabolizable protein in dairy cows, has a similar essential amino acid composition to milk (3). The elevated milk protein yield and milk N secretion within the M-SPR group might be partly attributed to its increased MCP synthesis. Furthermore, the heightened glucose concentration in the M-SPR group might diminish the necessity for certain amino acids in gluconeogenesis. Consequently, this elevated glucose concentration might also account for the increased milk protein yield in the M-SPR group.

In our study, the higher MUN and BUN concentration in the L-SPR group (control) indicated a lower degree of synchronization in energy and N supply within their rumen (48). The increased proportion of BCVFA in the L-SPR group (control) suggested that more amino acids were degraded to provide energy, leading to a subsequent rise in NH_3_-N concentration. Since the diets were designed to be similar RDP level in our study, the increased proportion of urinary nitrogen to N intake in L-SPR group (control) was likely due to inadequate ruminal energy supply stemming from a lower dietary RDS level. Therefore, the lower NUE in L-SPR group (control) was attributed to the increased proportion of urinary nitrogen to N intake. Similar results were also reported by Kand and Dickhoefer (49). Furthermore, despite having the highest RDS levels, the H-SPR group did not exhibit a high NUE as the M-SPR group. This might be due to the higher proportion of fecal N to N intake in this group. The H-SPR group had a diet with a higher level of HSBM, which had lower intestinal digestibility (50), consequently increasing fecal N excretion.

Although an increase in TVFA concentration was observed in our study, the dietary SPR did not impact rumen pH. This might be attributed to the adequate dietary forage NDF and peNDF among treatments (8, 51). Within the normal range of rumen pH, a moderate dietary RDS level was observed to enhance the proliferation of cellulolytic and amylolytic bacteria compared to diets with low or high RDS levels (52). Moreover, Zhang et al. (53) reported a positive association between the MCP synthesis and the relative abundance of cellulolytic and amylolytic bacteria. Consequently, the increased synthesis of MCP in the M-SPR group might enhance the abundance of cellulolytic and amylolytic bacteria in the rumen. This, in turn, could lead to improved digestibility of starch and fiber for dairy cows, accompanied by a corresponding increase in ruminal TVFA concentration.

## 5 Conclusion

The dietary SPR emerges as a novel indicator, offering insights into the availability of energy and nitrogen within the rumen. A lower dietary SPR correlated with an increase in the proportion of urinary nitrogen excretion, while a higher dietary SPR was associated with a reduction in dry matter intake. Balancing dietary SPR could boost lactation performance and nitrogen utilization efficiency by improving MCP synthesis and nutrient digestibility. Considering the economic benefits and environmental protection, this study recommends a medium dietary SPR (with SPR = 2.3) for mid-lactating Holstein dairy cows. Nevertheless, further investigations on rumen microbial composition and metabolites are required to elucidate the underlying mechanisms responsible for the observed effects.

## Data availability statement

The original contributions presented in the study are included in the article/supplementary material, further inquiries can be directed to the corresponding authors.

## Ethics statement

The animal study was approved by the Institutional of Animal Care and Use Committee at Hebei Agricultural University. The study was conducted in accordance with the local legislation and institutional requirements.

## Author contributions

PC: Data curation, Methodology, Software, Writing – original draft. YL: Methodology, Writing – review & editing. MW: Data curation, Writing – review & editing. YS: Formal analysis, Funding acquisition, Writing – review & editing. ML: Writing – review & editing. HX: Writing – review & editing. NM: Writing – review & editing. YC: Writing – review & editing. QL: Writing – review & editing. MA: Writing – review & editing. ZW: Validation, Writing – review & editing. ZH: Visualization, Writing – review & editing. SR: Visualization, Writing – review & editing. LH: Methodology, Writing – review & editing. JL: Software, Writing – review & editing. YG: Funding acquisition, Methodology, Software, Supervision, Writing – review & editing. JgL: Funding acquisition, Investigation, Project administration, Resources, Supervision, Writing – review & editing.
