# Supplementary material for: Optimizing dietary rumen-degradable starch to rumen-degradable protein ratio improves lactation performance and nitrogen utilization efficiency in mid-lactating Holstein dairy cows
Source: Front Vet Sci. 2024 Feb 29;11:1330876. doi: 10.3389/fvets.2024.1330876 (PMC10938912; doi:10.3389/fvets.2024.1330876)
Supplement: Supplementary file 1 [file Data_Sheet_1.docx]

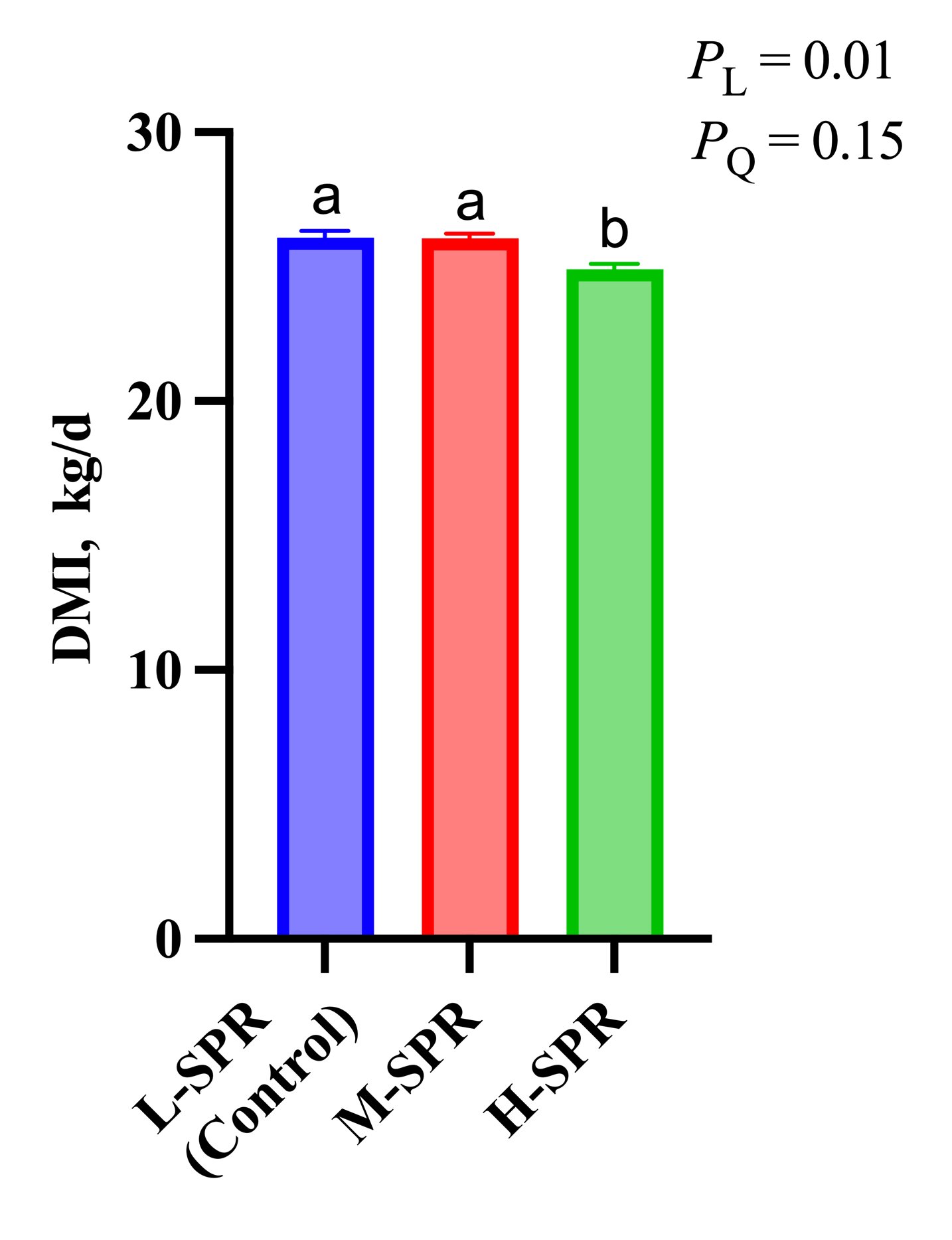

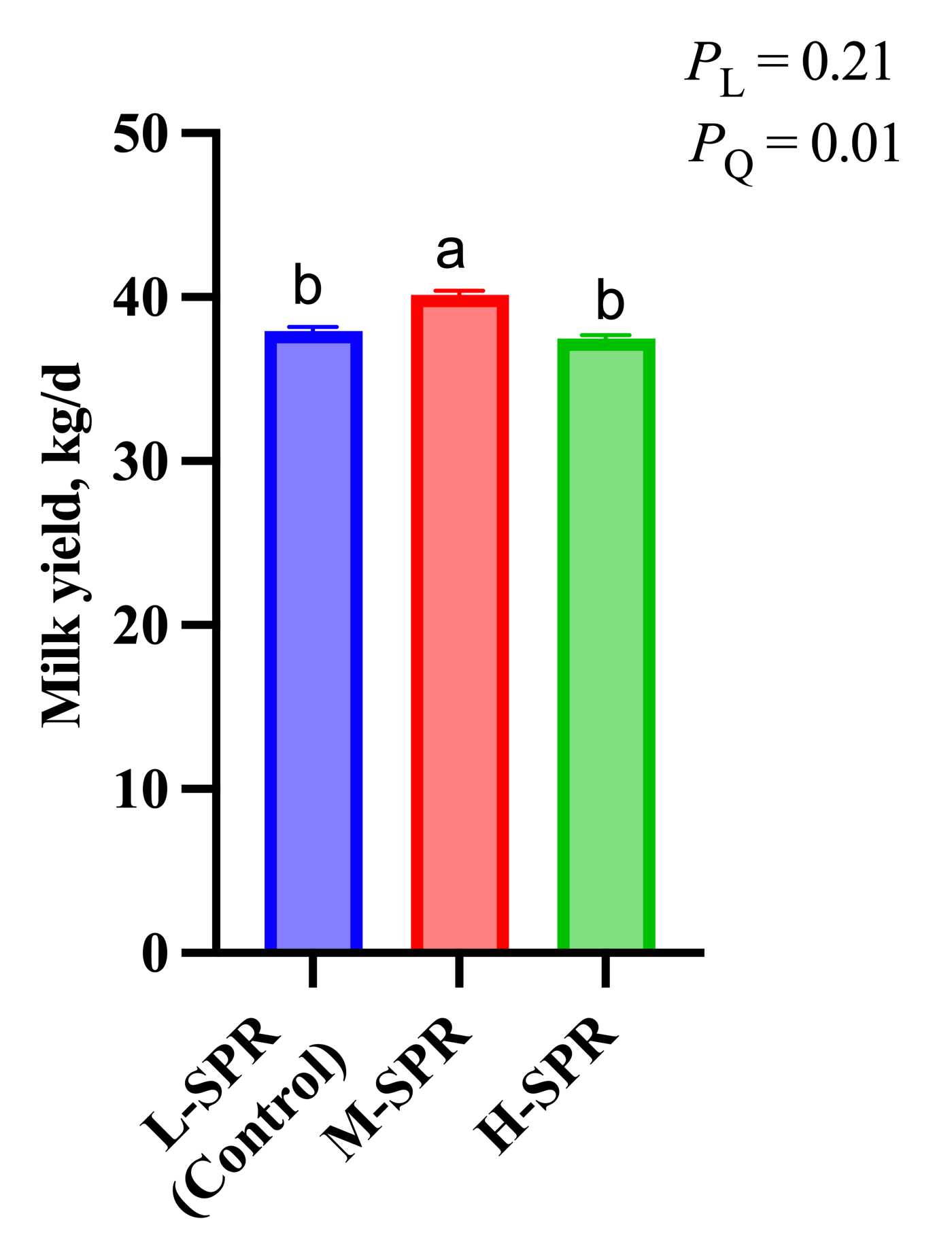

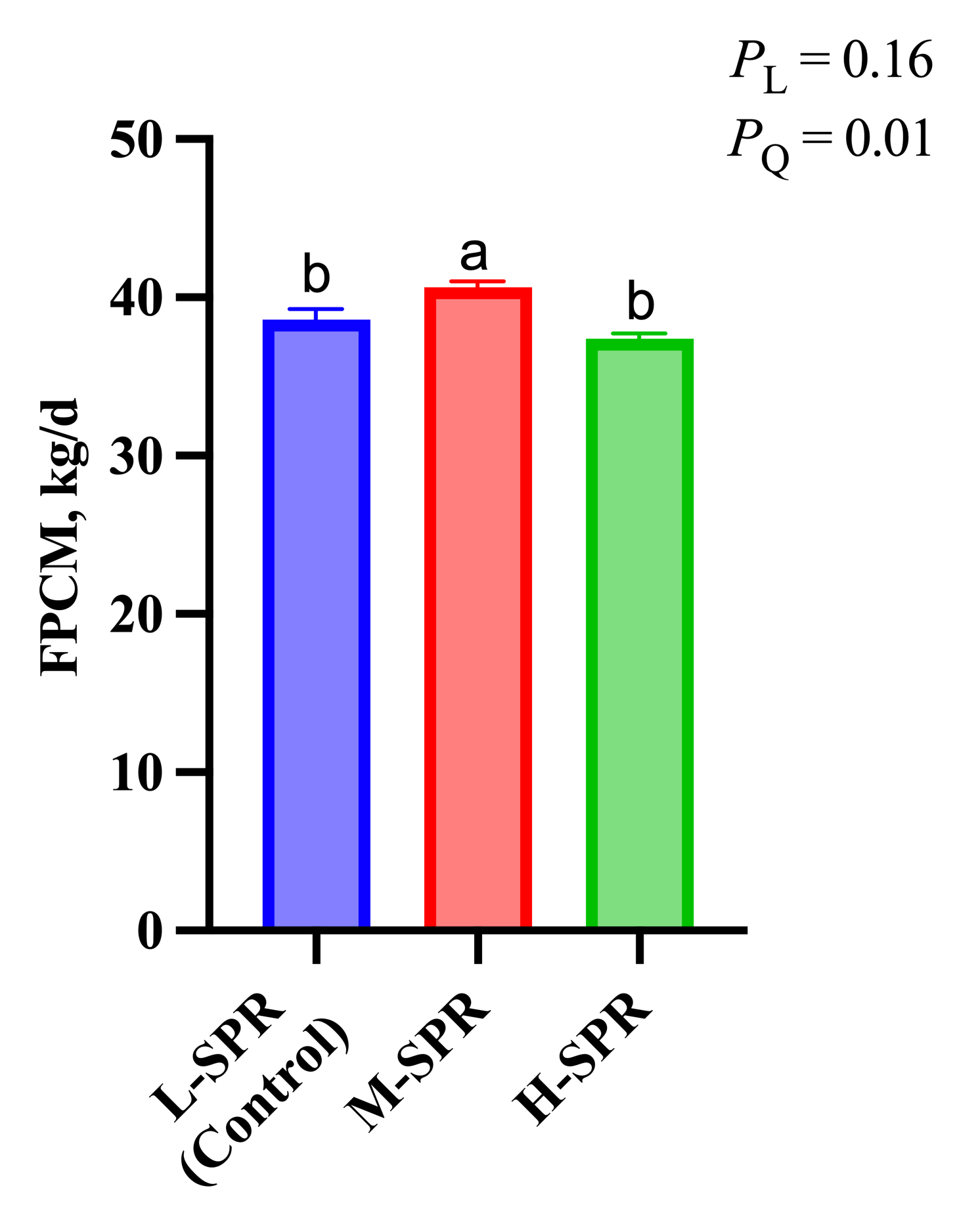

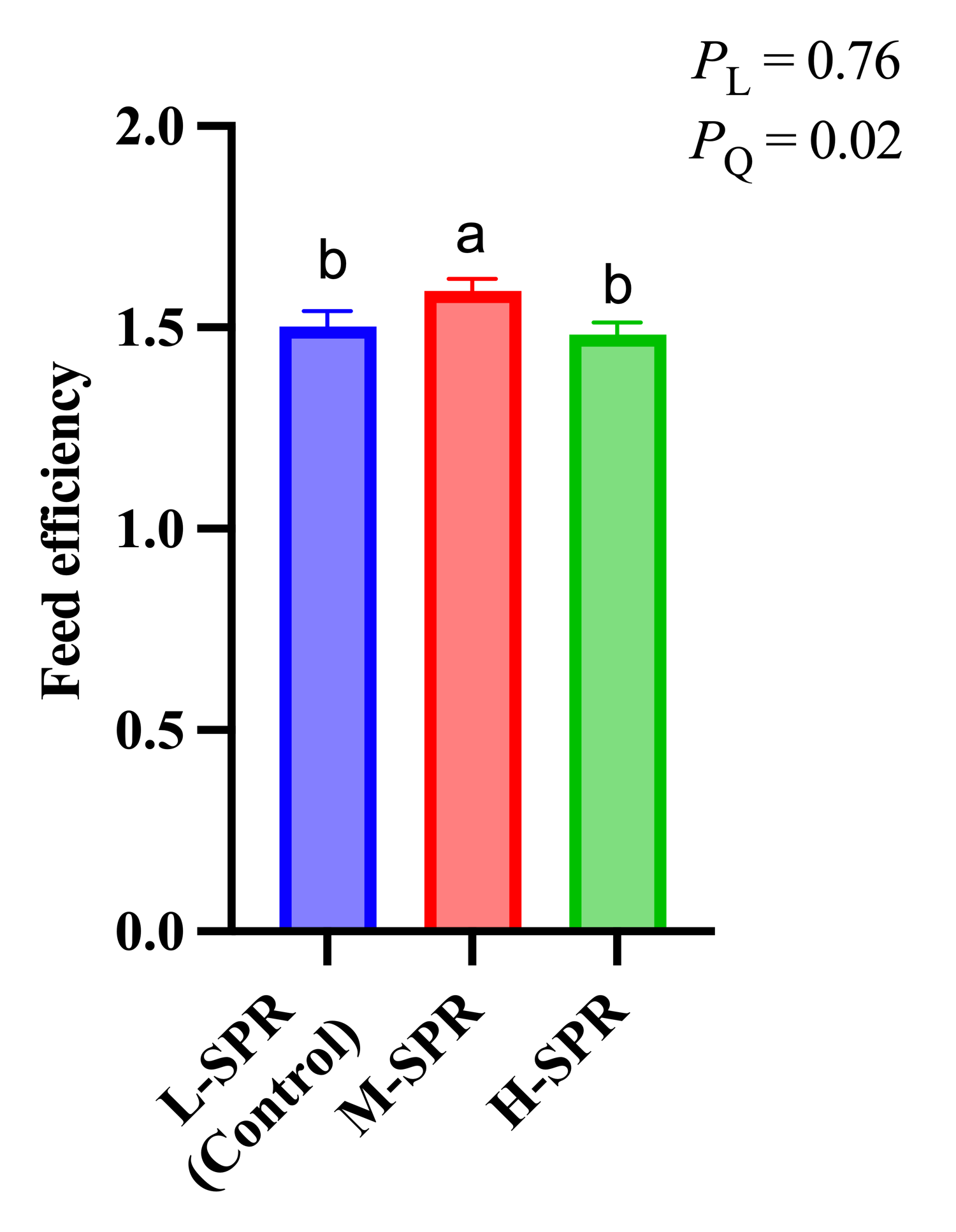


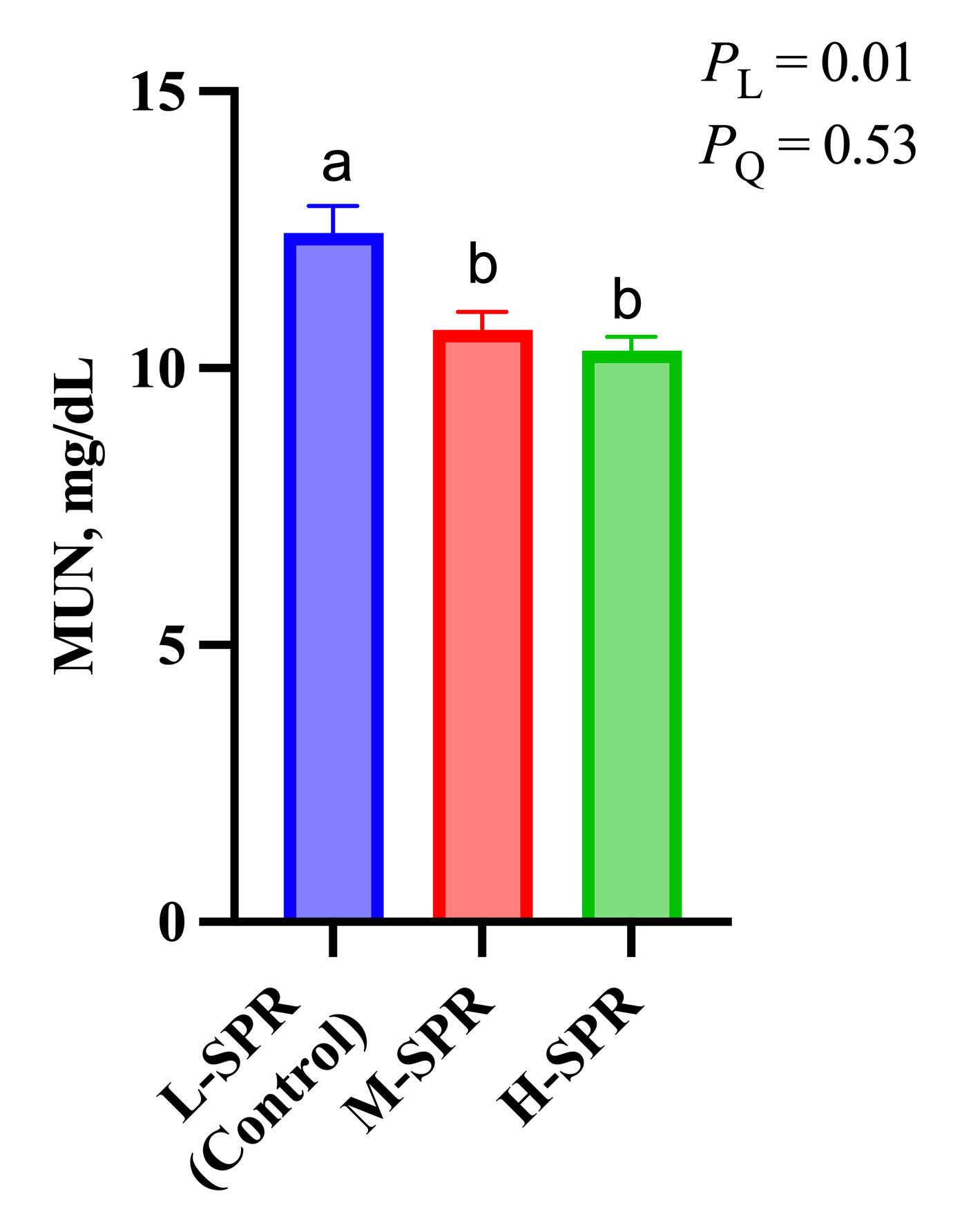

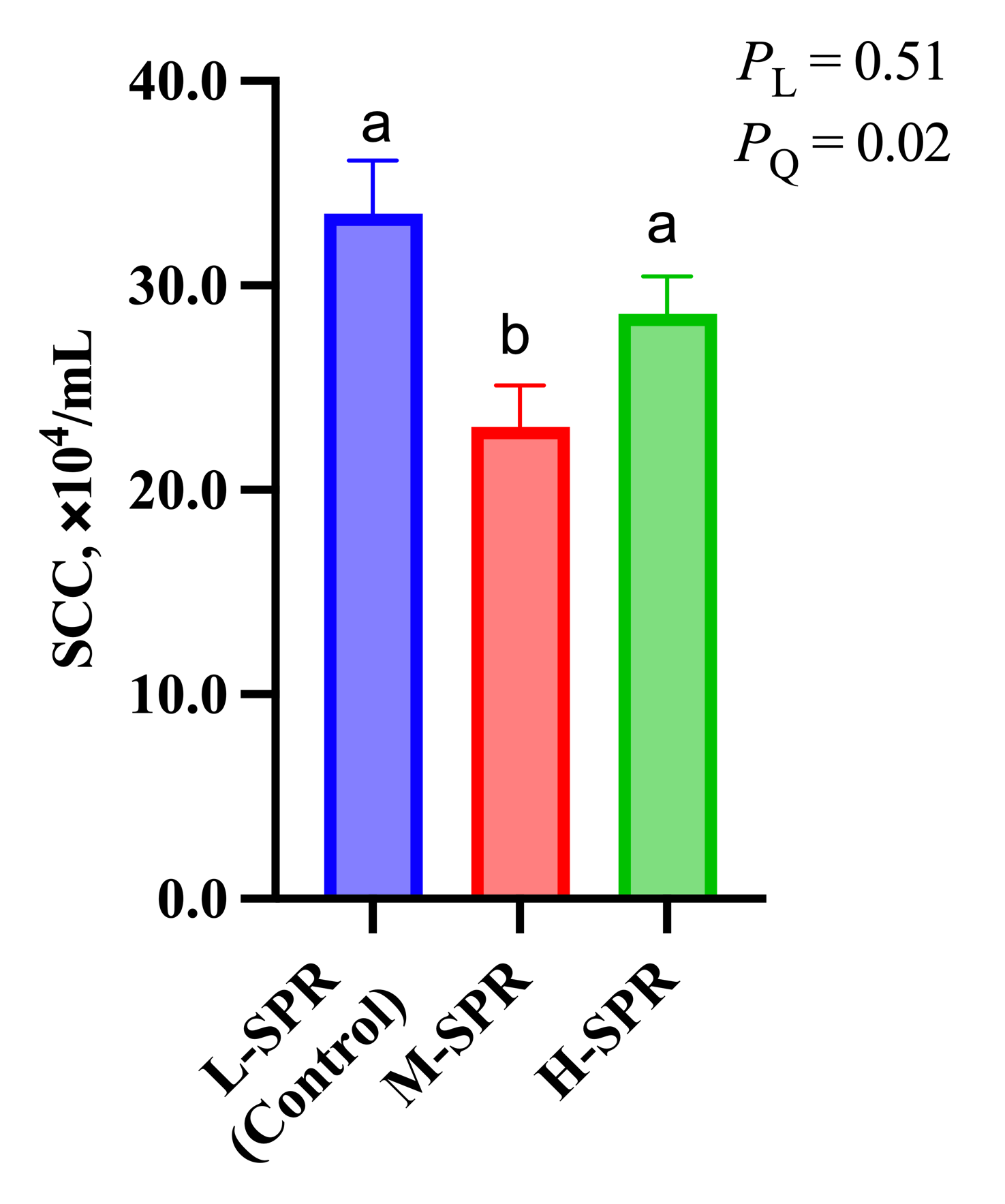

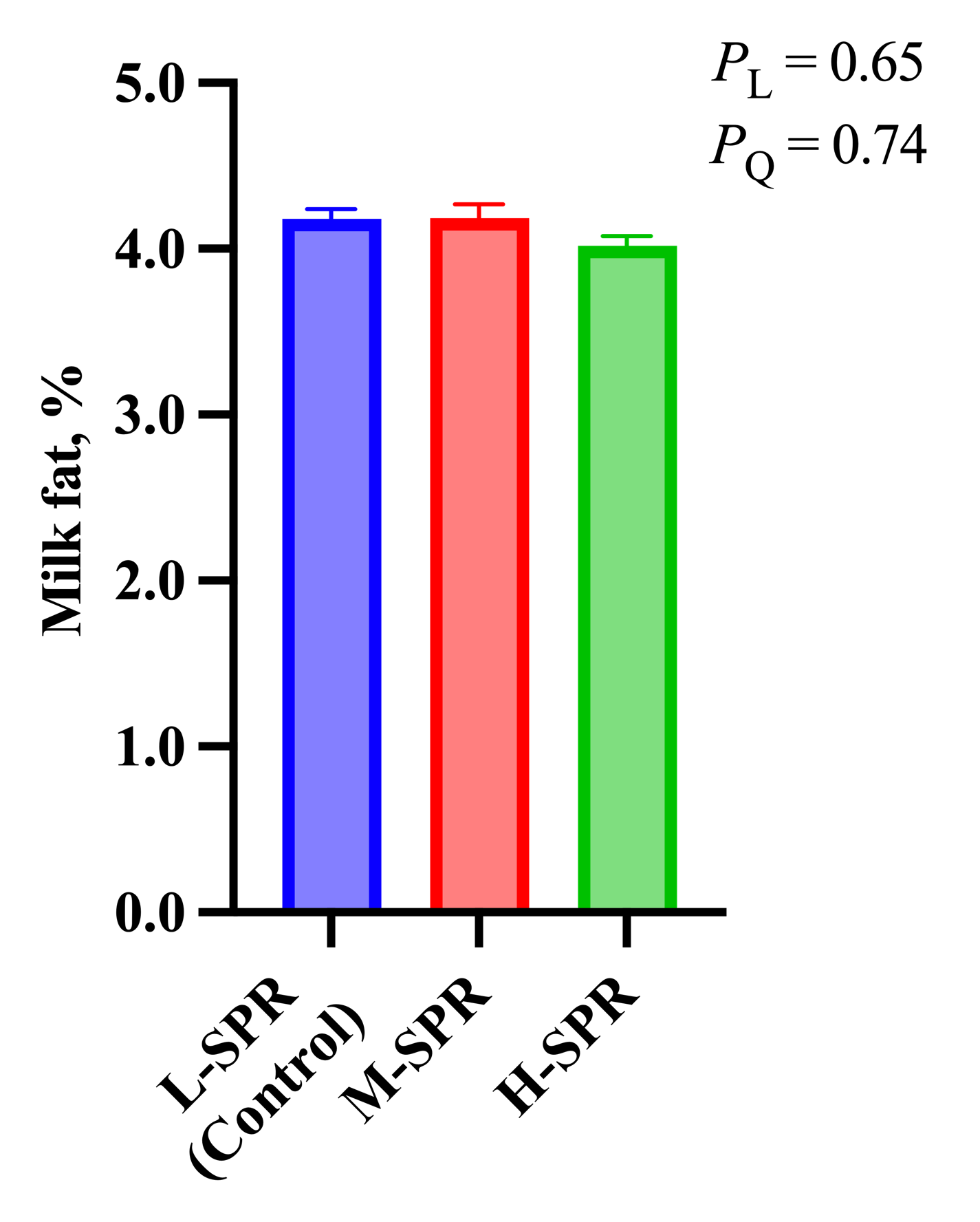

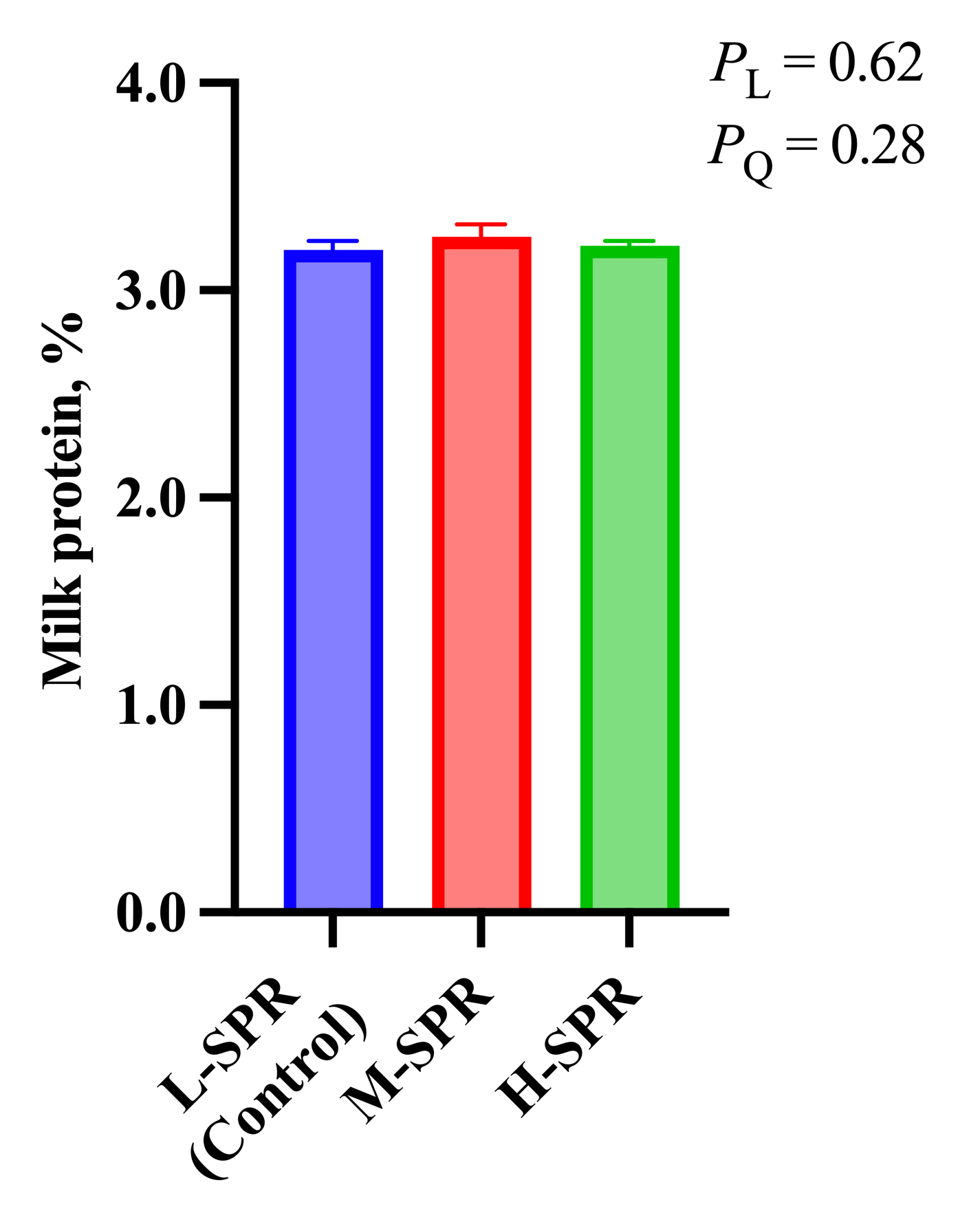


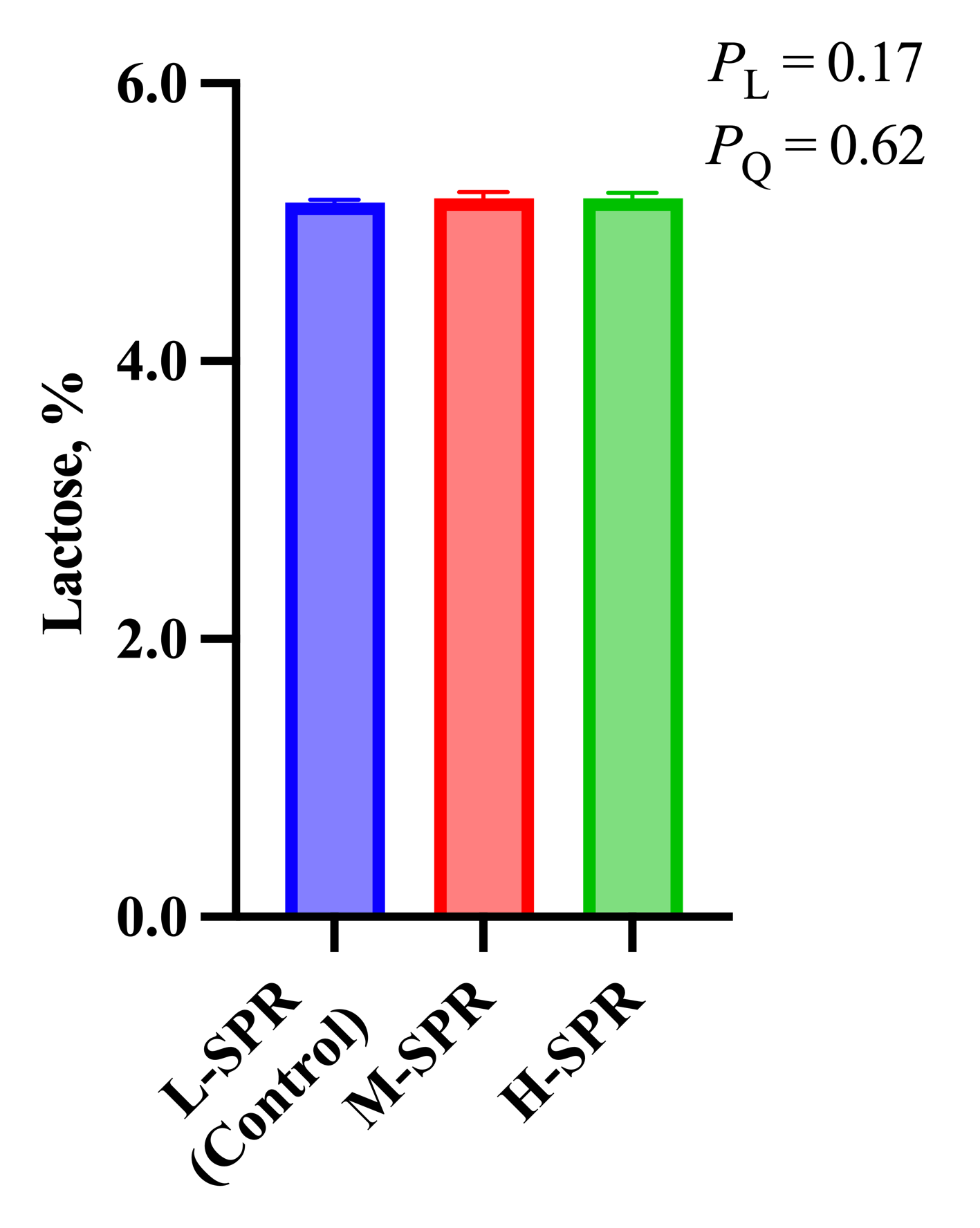

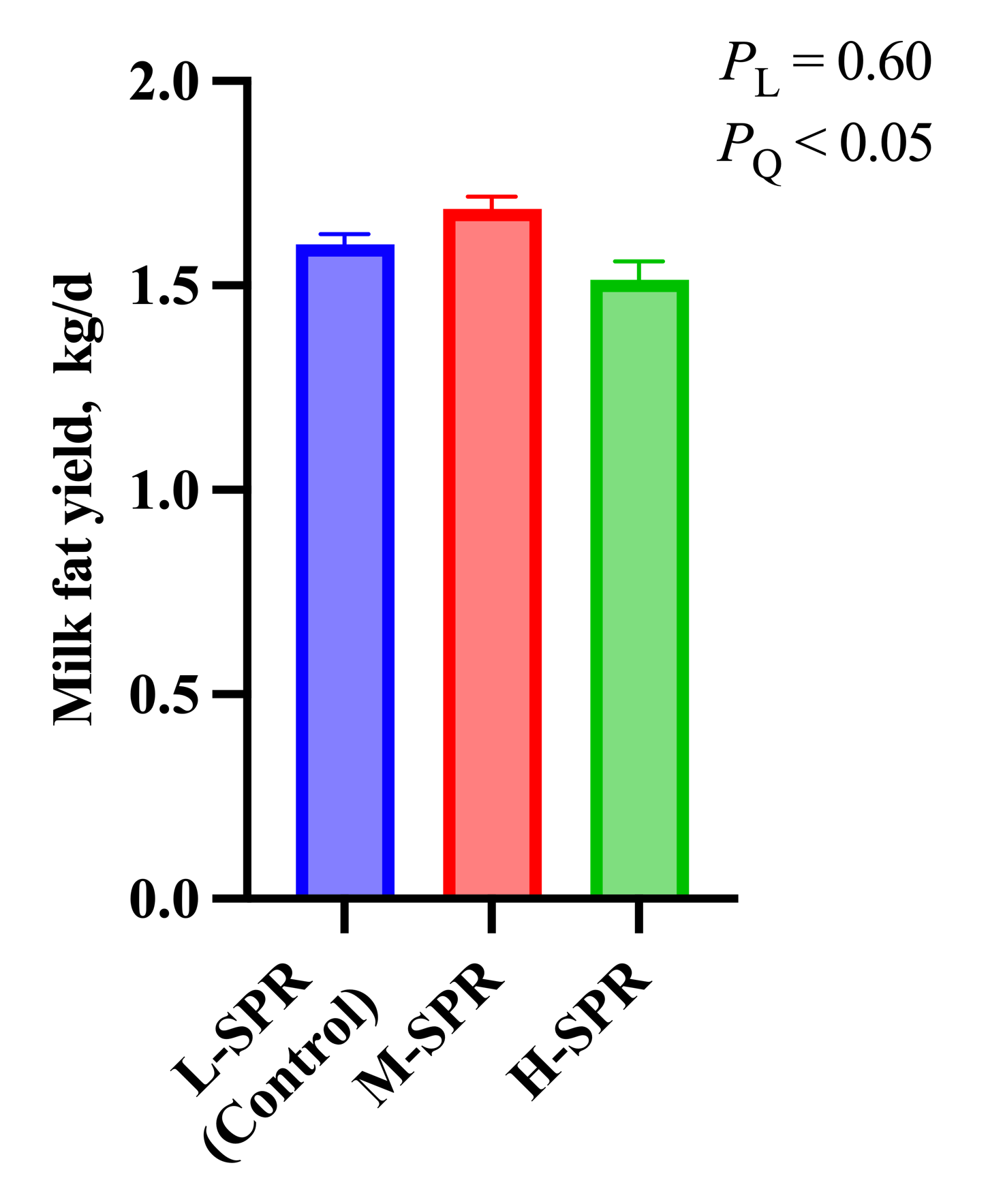

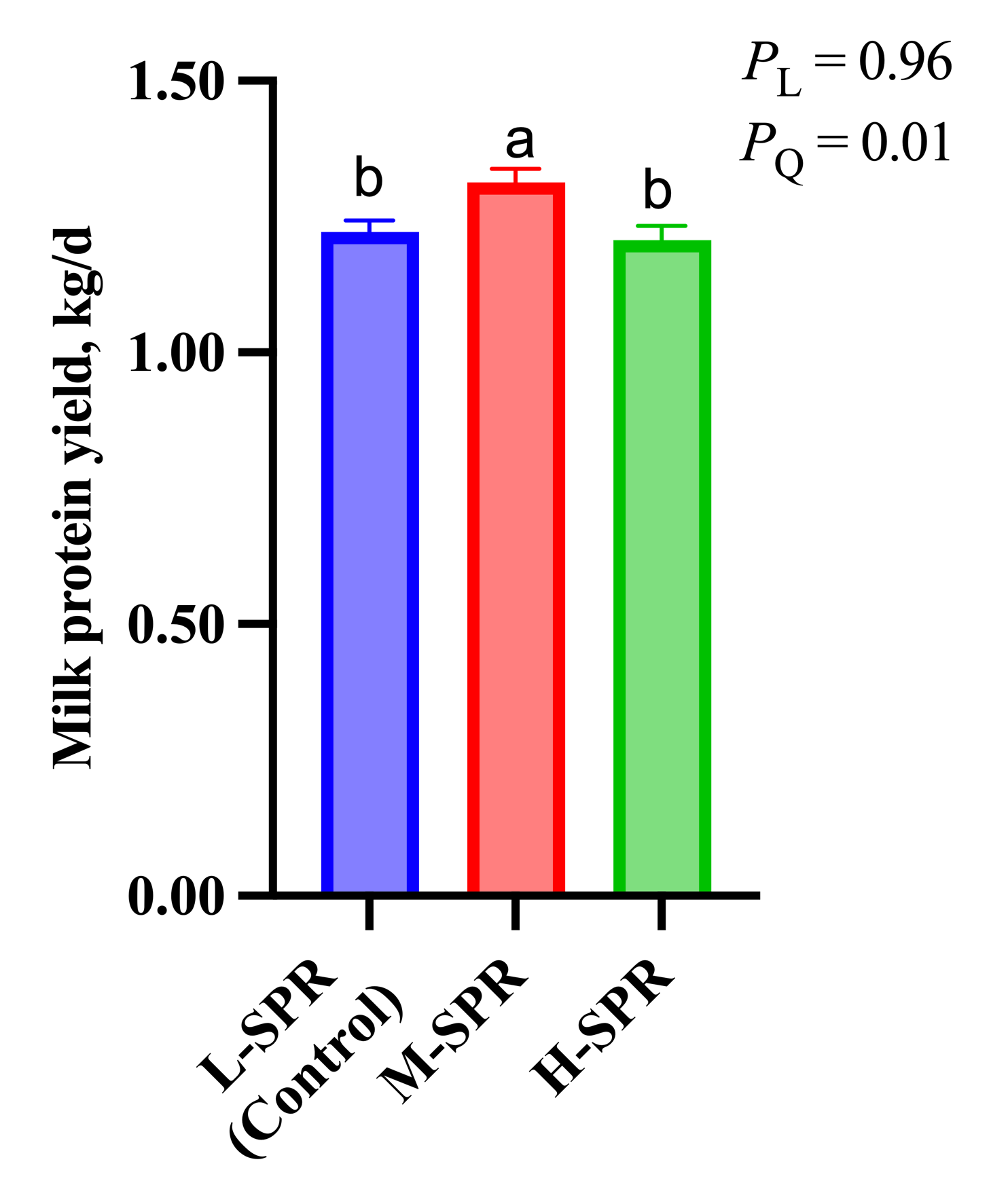

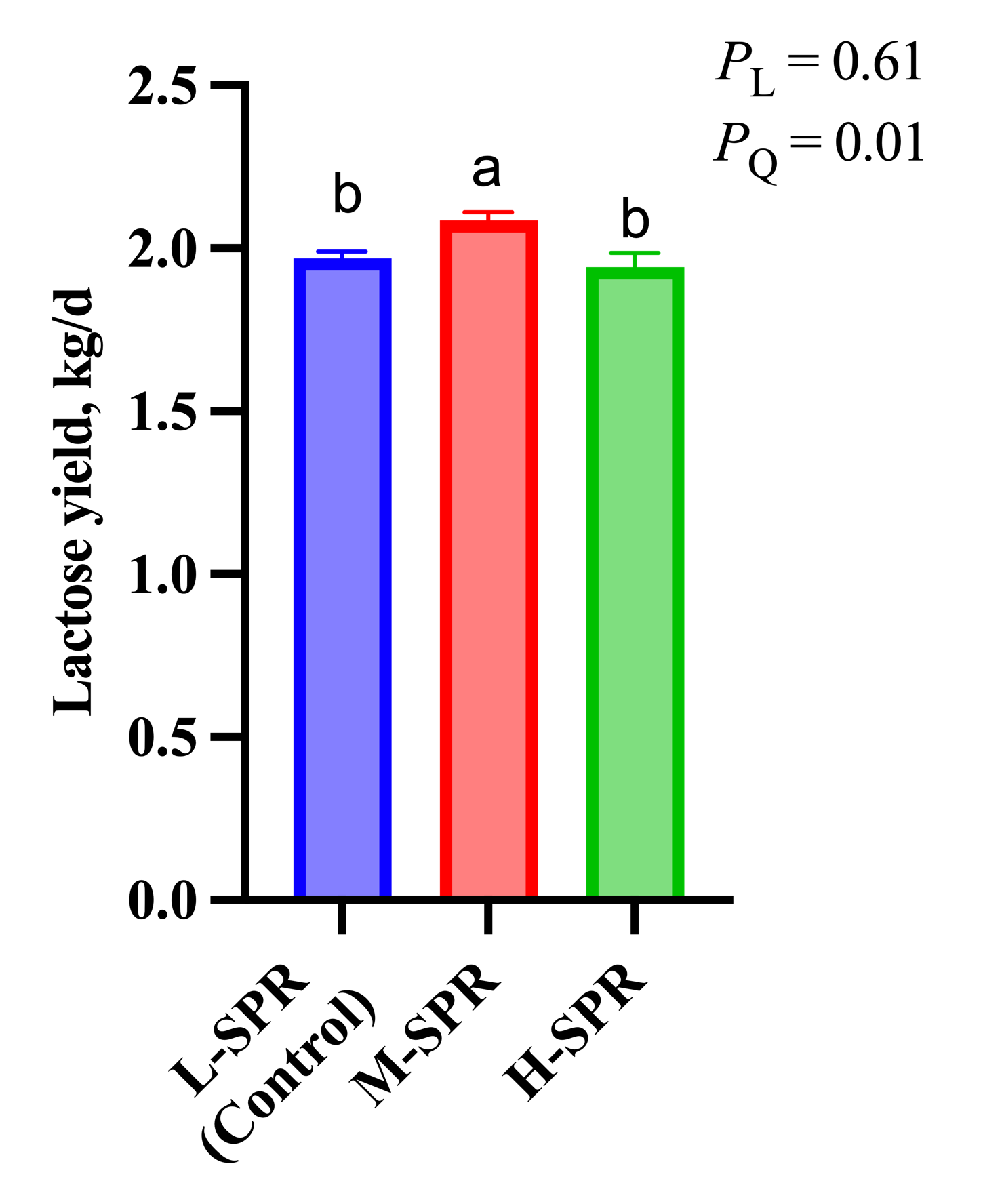


**Figure 1.** Effect of dietary rumen-degradable starch to rumen-degradable protein ratio (SPR) on dry matter intake and lactation performance in mid-lactating Holstein cows. Error bars indicate measure of variation within the dietary SPRs. Different letters (a–b) indicate statistically significant difference (*p* < 0.05). L is linear, and Q is quadratic effects for diet SPR; DMI = dry matter intake; MUN = milk urea nitrogen; FPCM = Fat- and protein-corrected milk; Feed efficiency = FPCM/DMI.


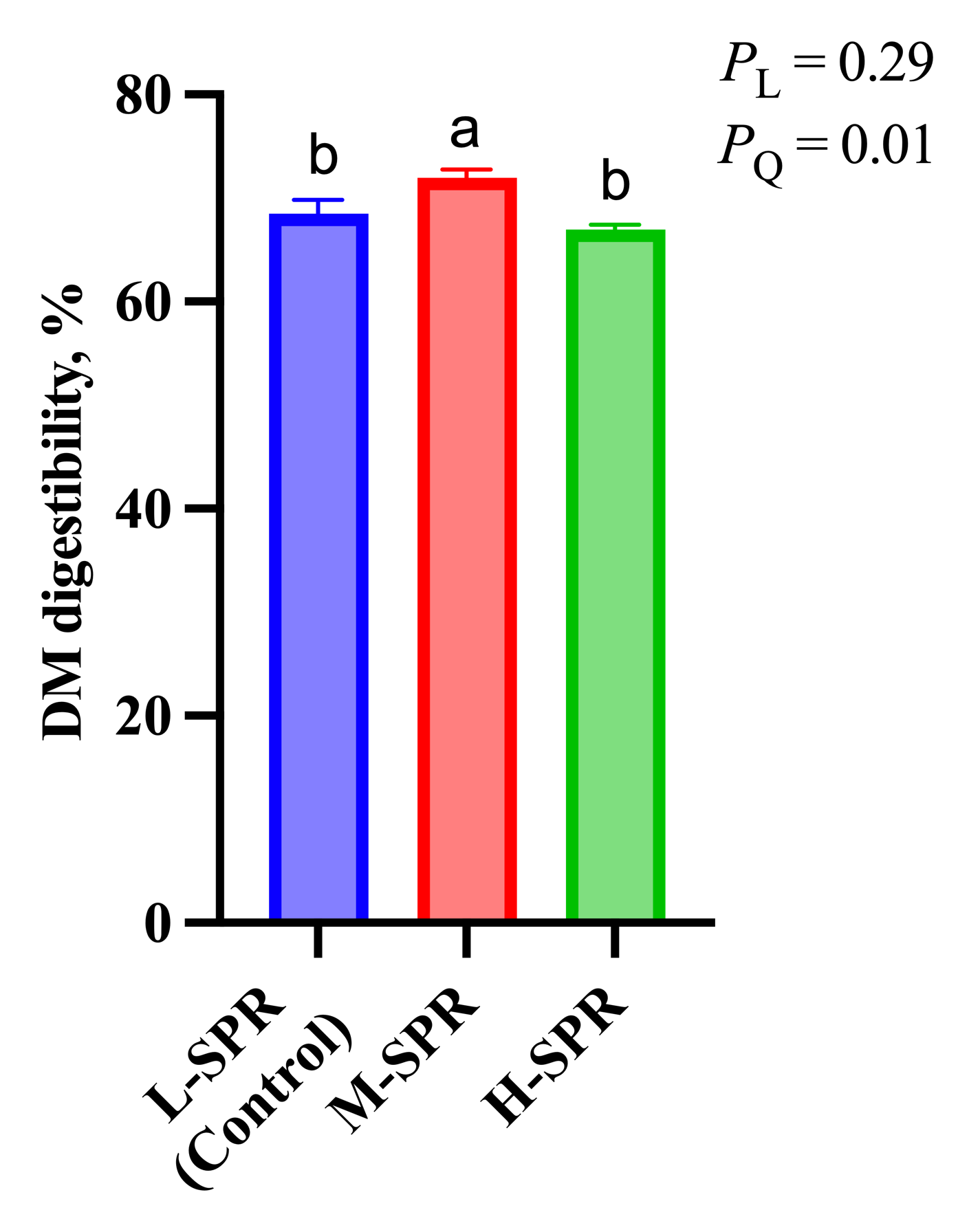

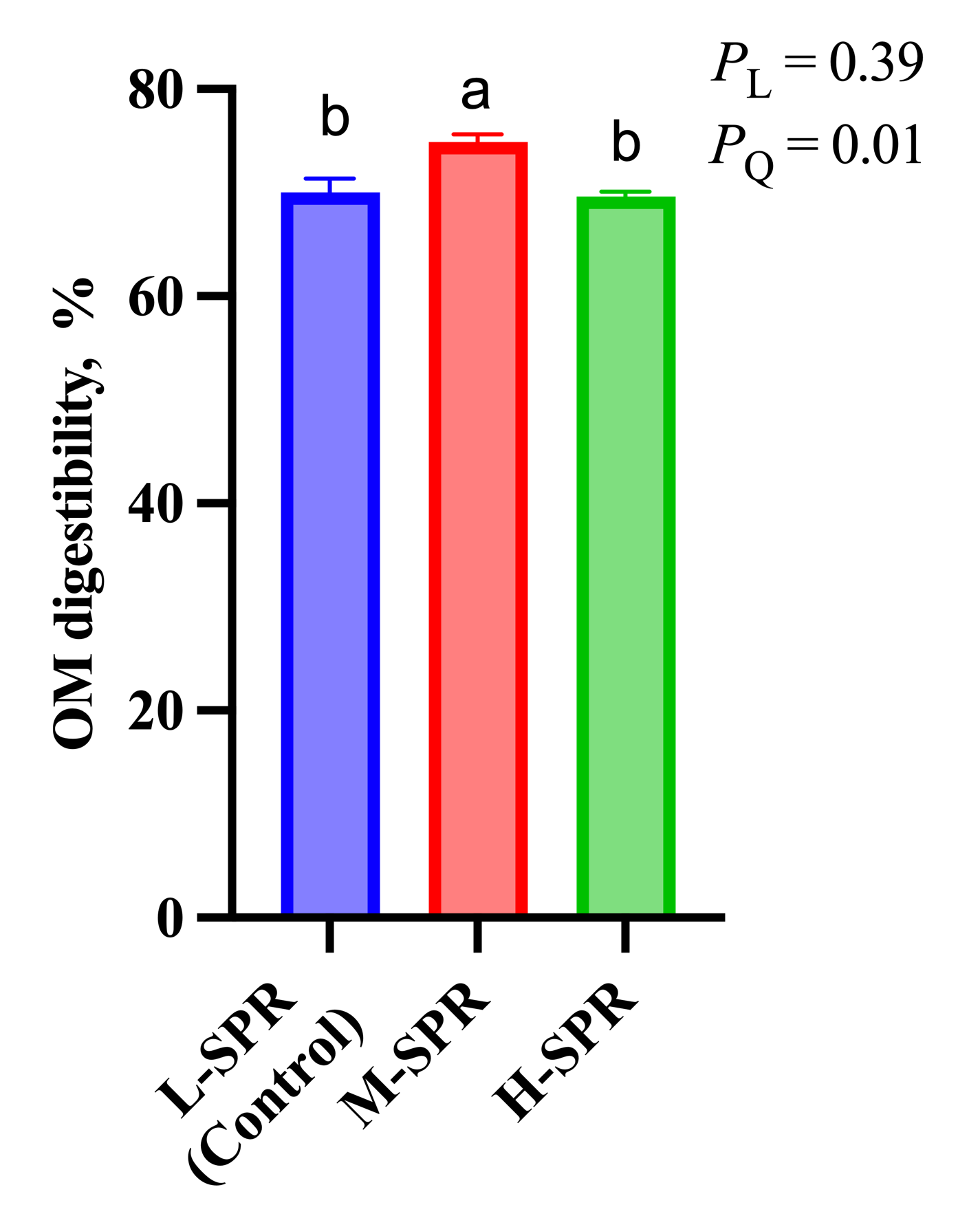

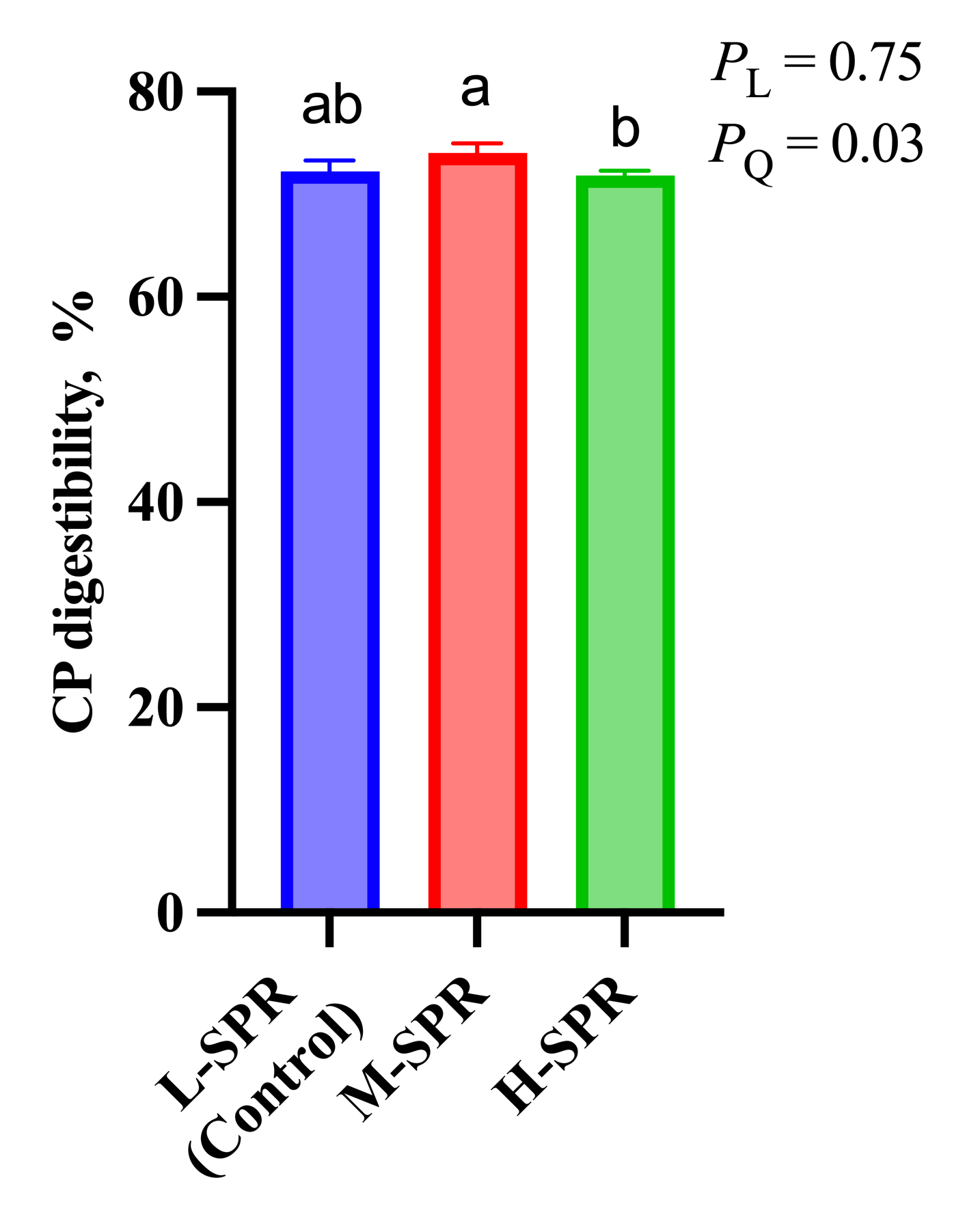

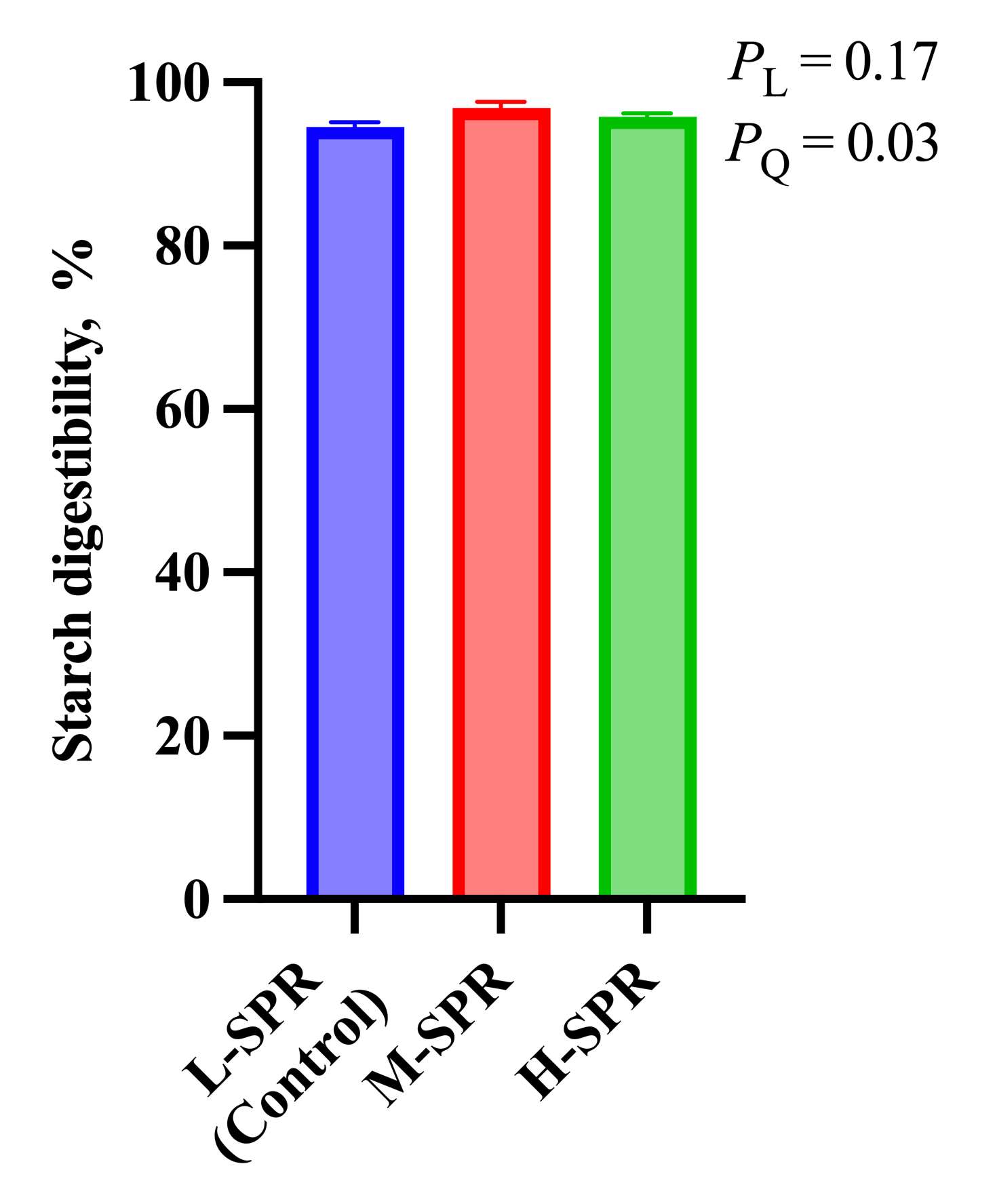


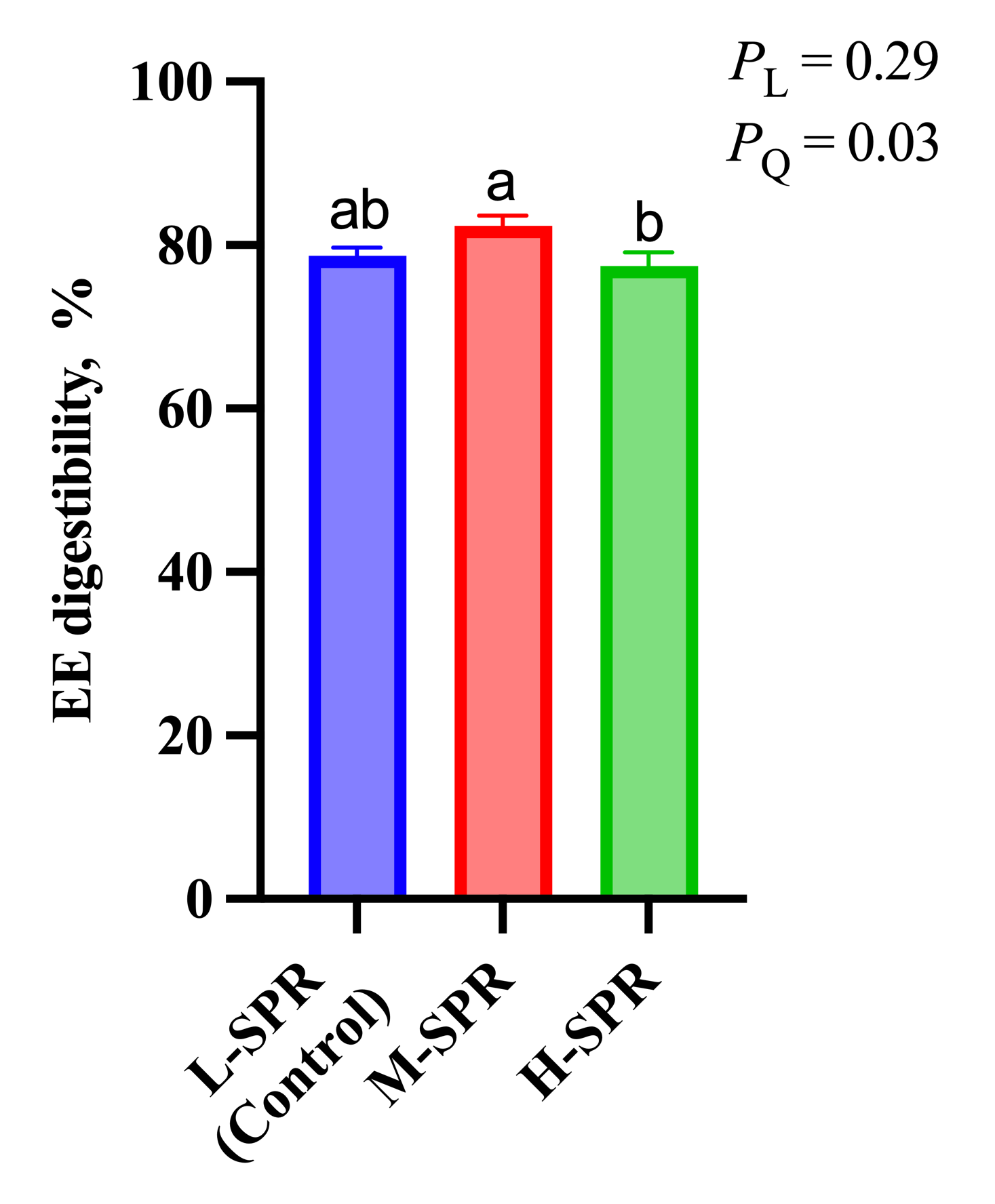

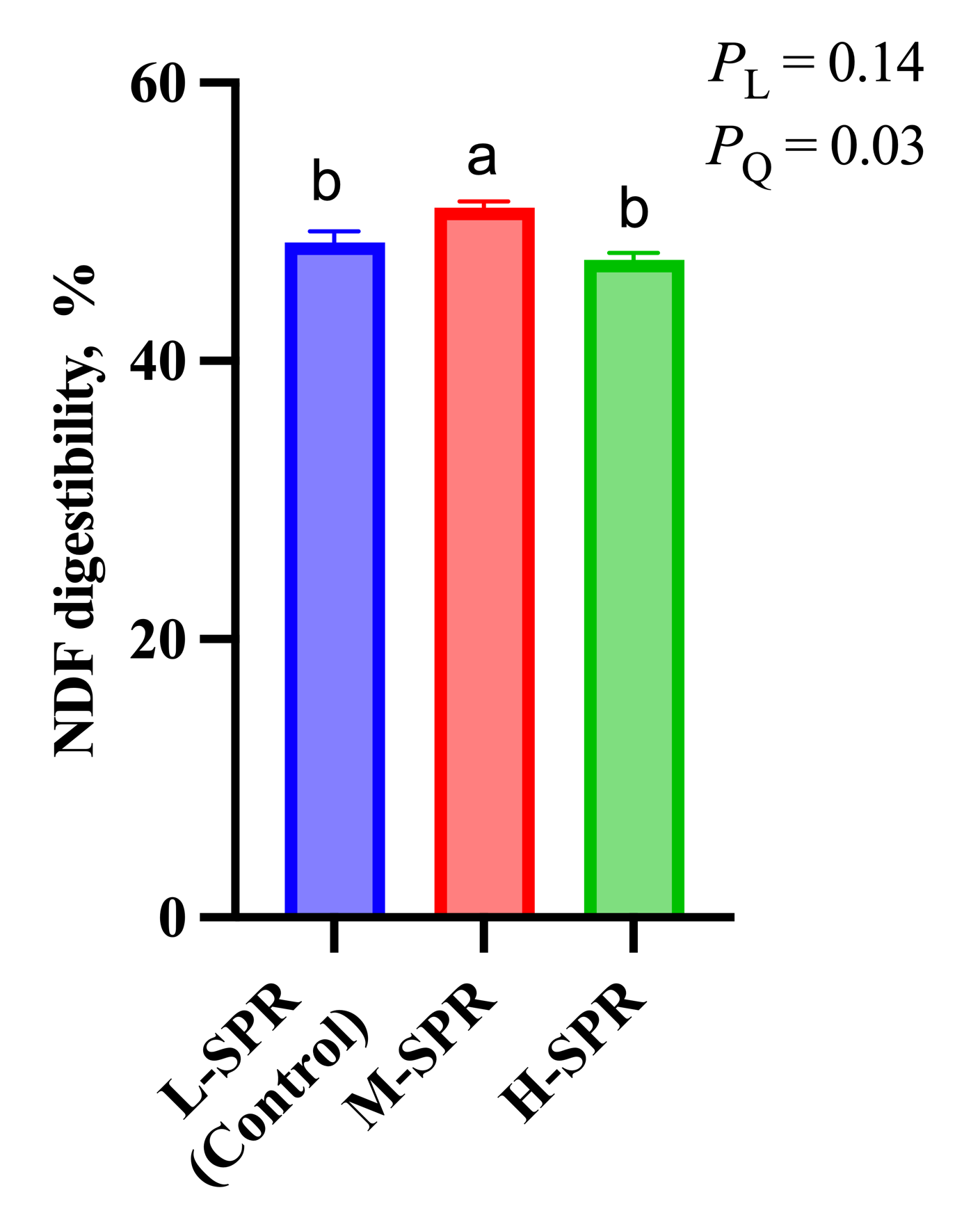

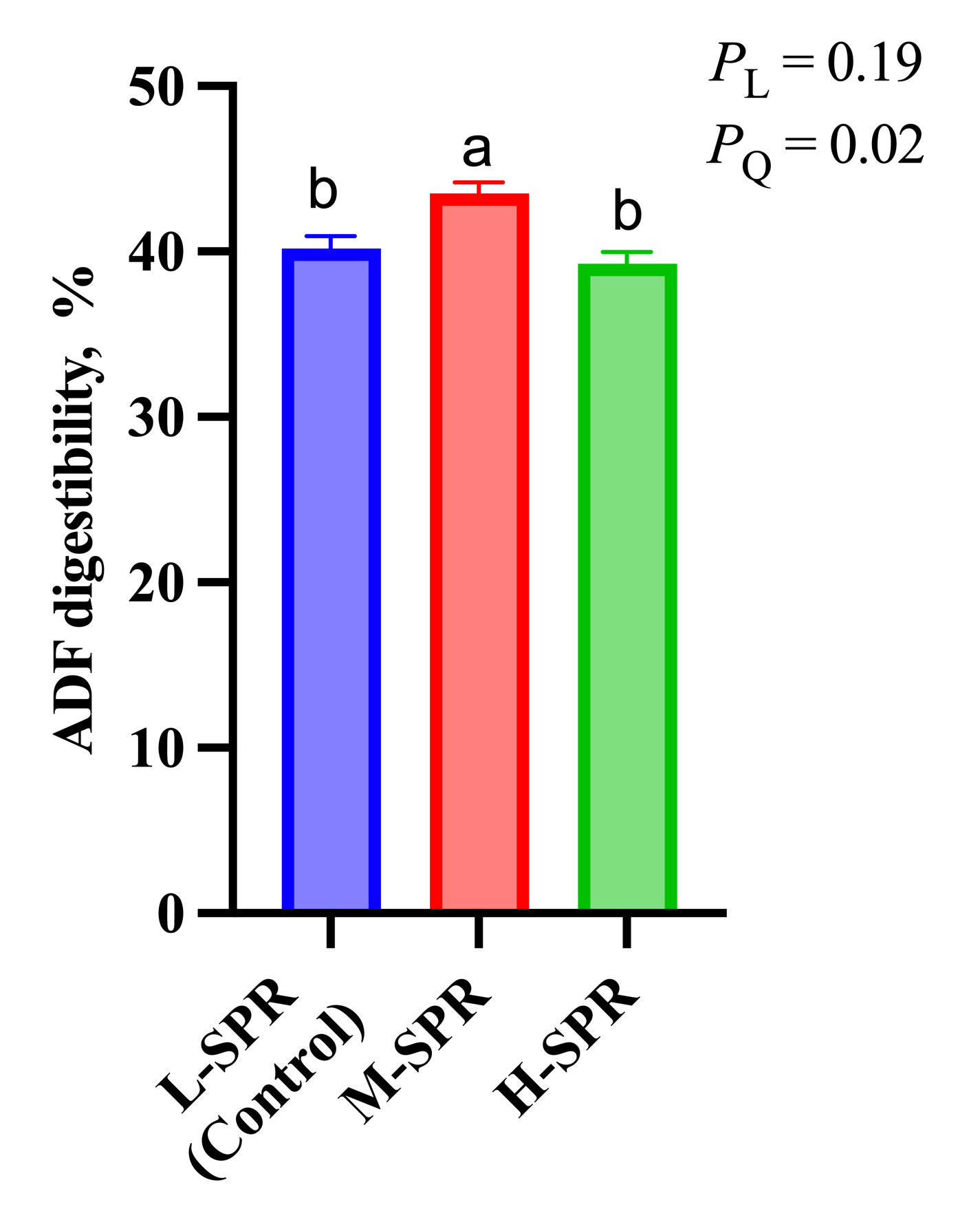


**Figure 2.** Effect of dietary rumen-degradable starch to rumen-degradable protein ratio (SPR) on nutrient apparent digestibility in mid-lactating Holstein cows. Error bars indicate measure of variation within the dietary SPRs. Different letters (a–b) indicate statistically significant difference (*p* < 0.05). L is linear, and Q is quadratic effects for diet SPR; DM = dry matter; OM = organic matter; CP = crude protein; EE = ether extract; NDF = neutral detergent fiber; ADF = acid detergent fiber.

^
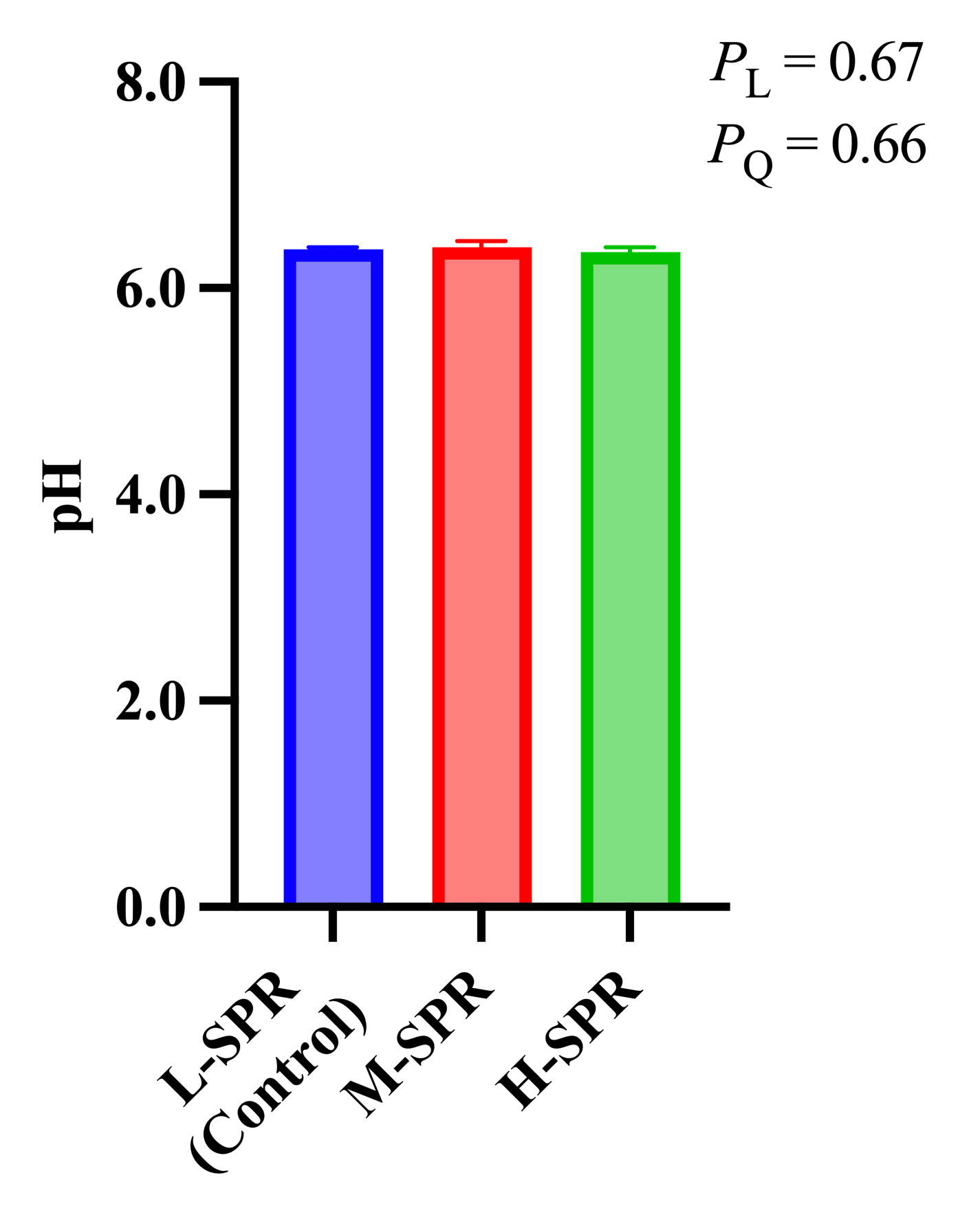

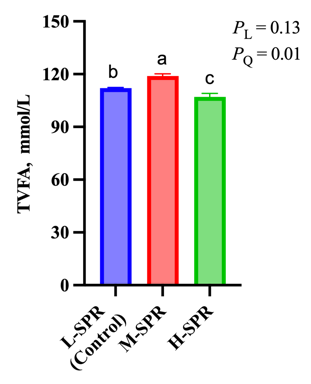

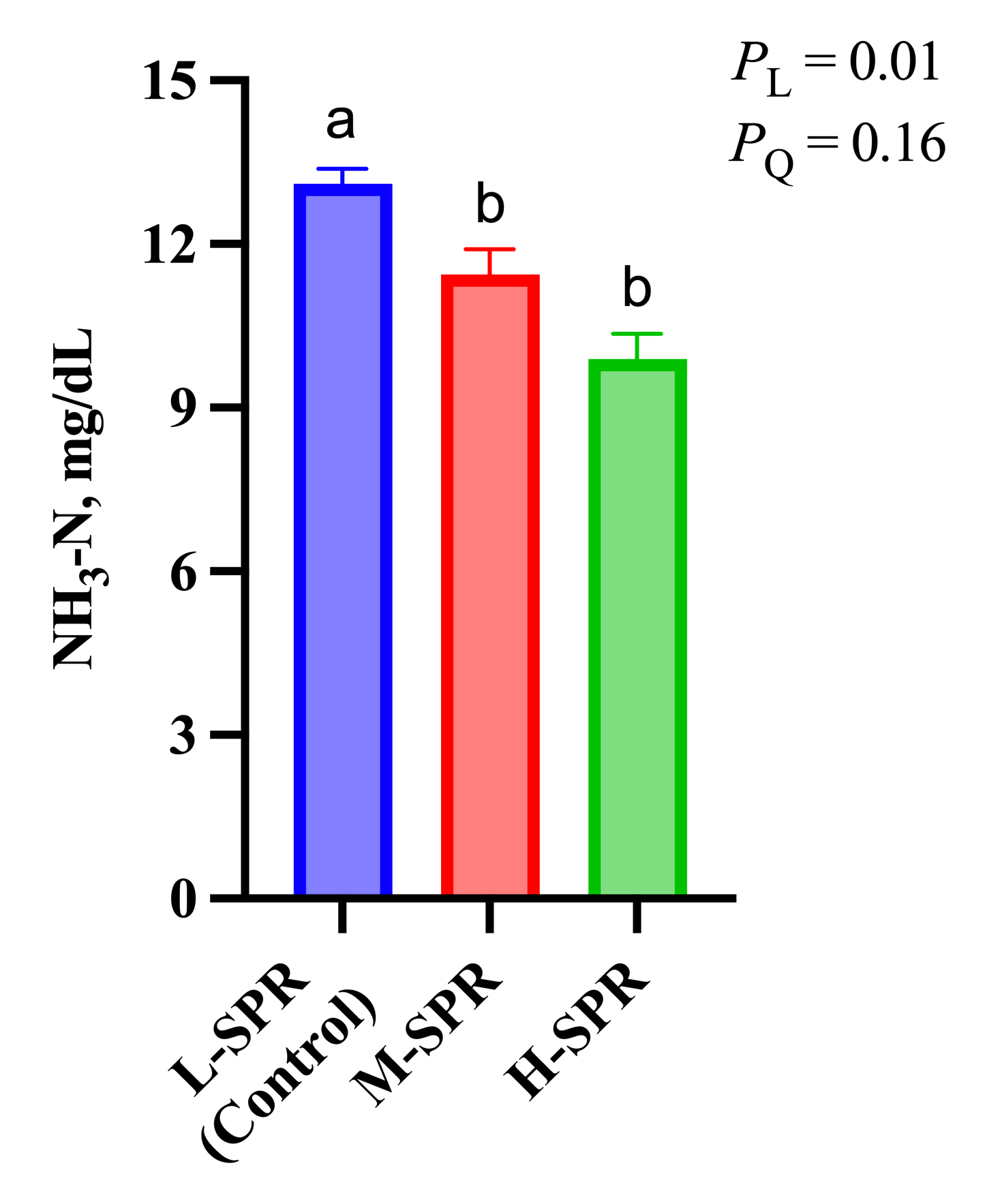

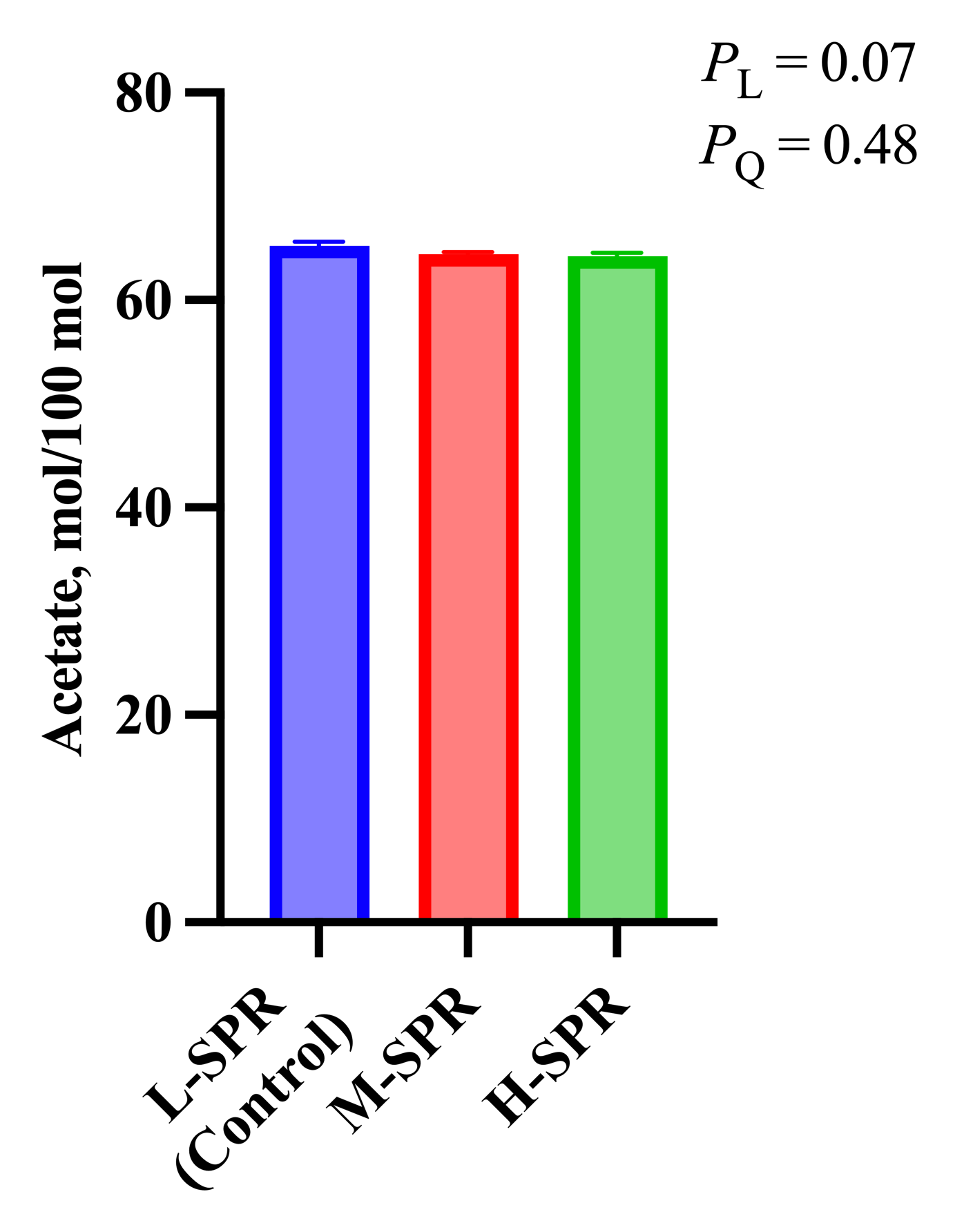

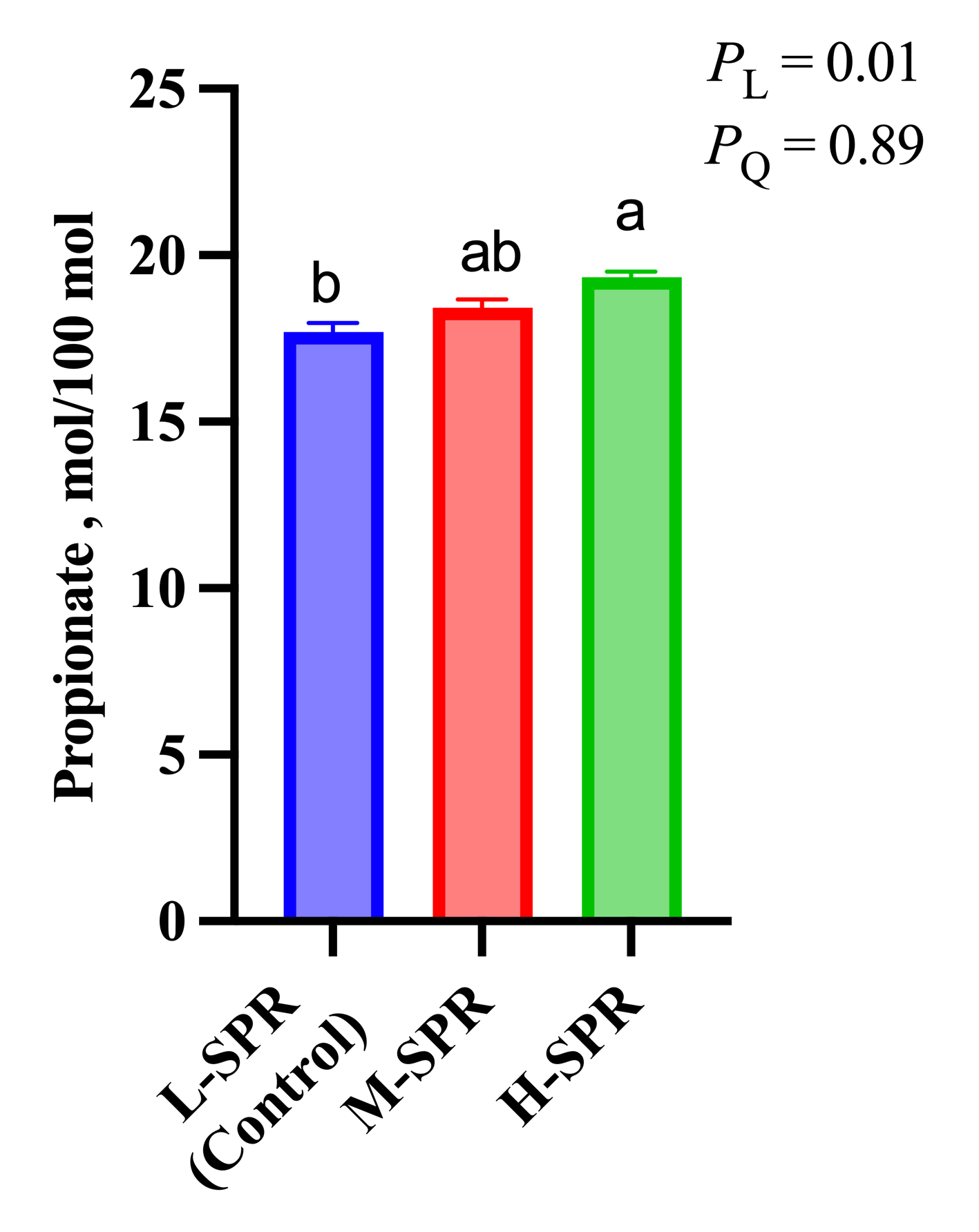

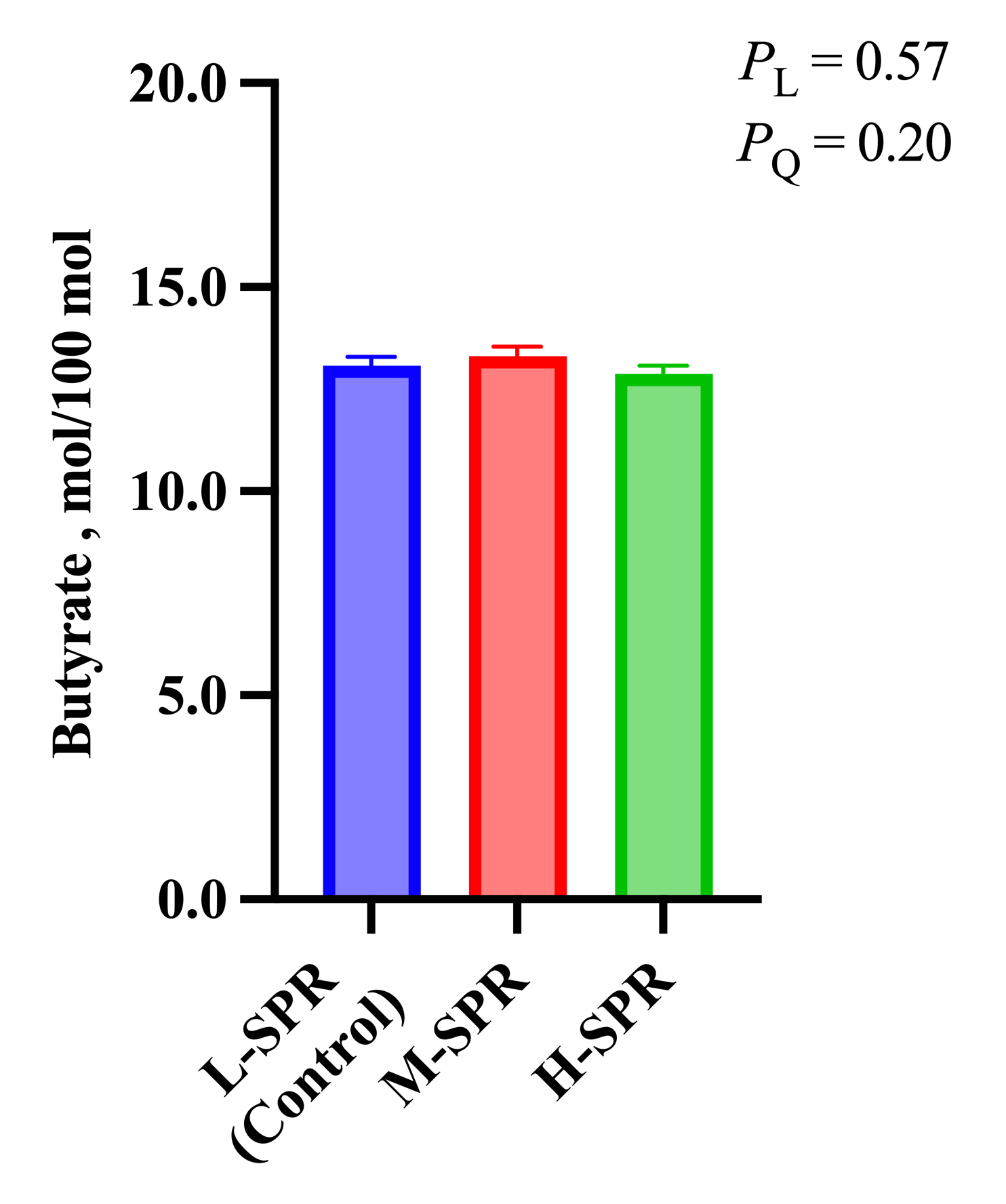

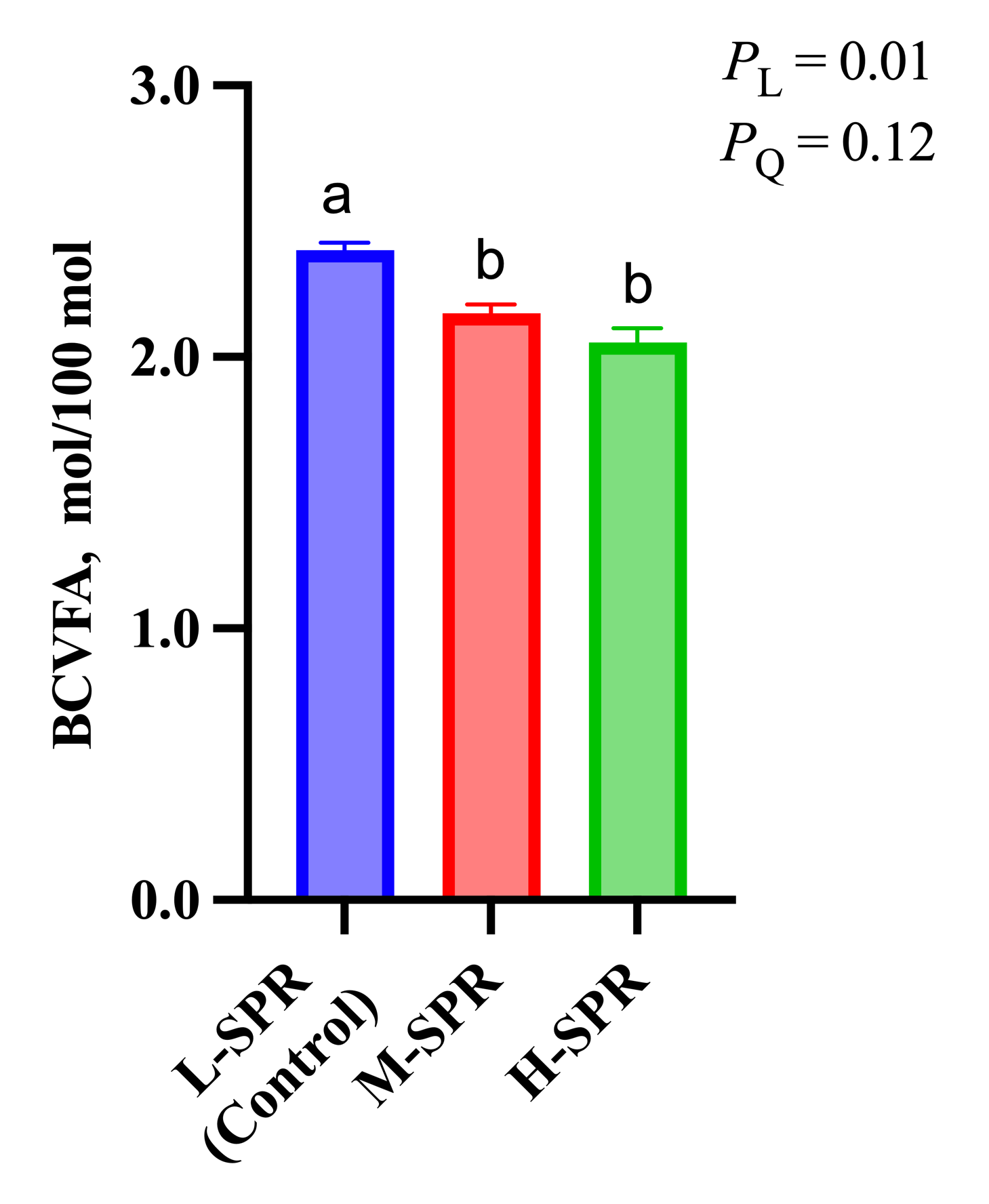

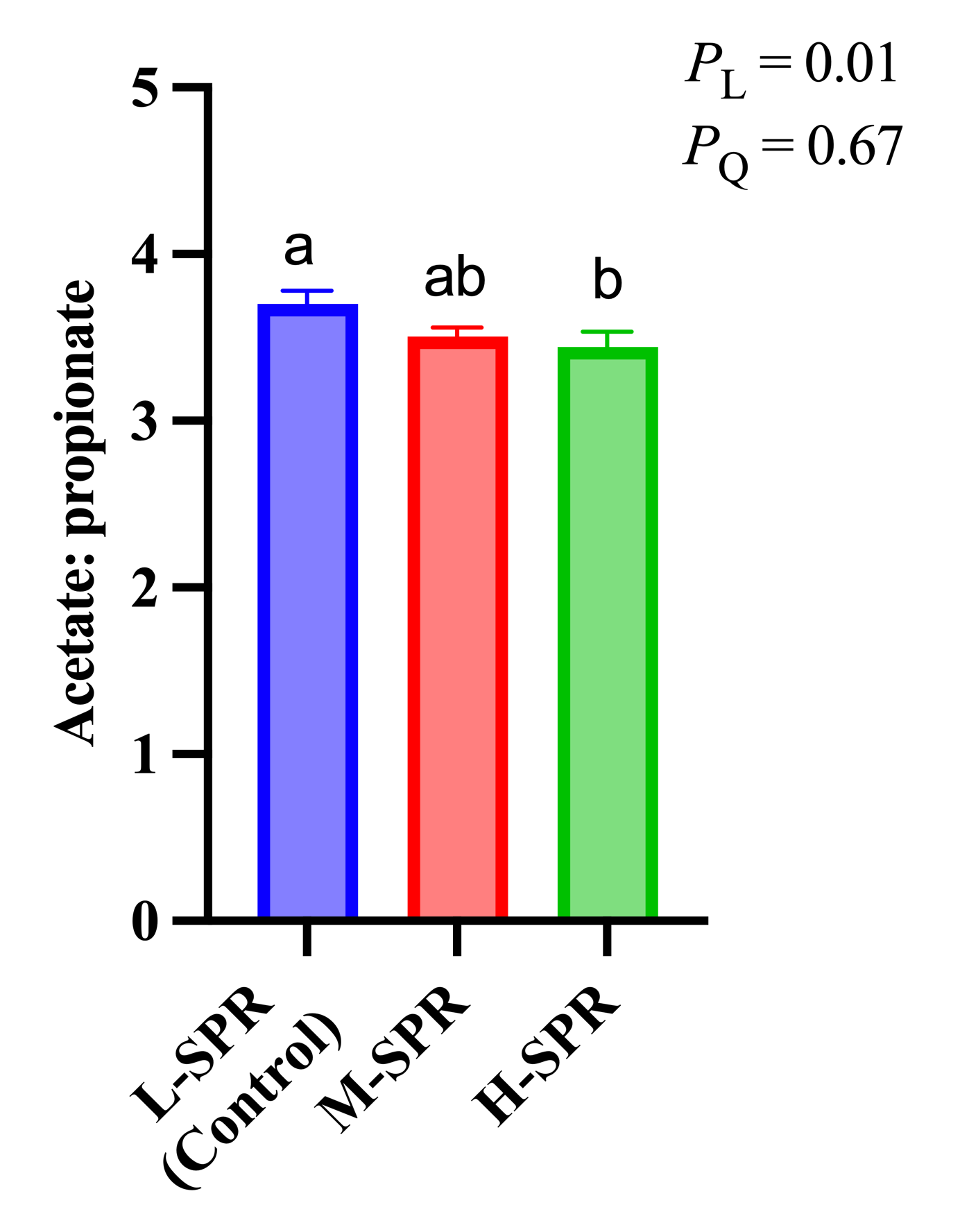
^

**Figure 3.** Effect of dietary SPR on rumen fermentation patterns in mid-lactating Holstein cows. Error bars indicate measure of variation within the dietary SPRs. Different letters (a–c) indicate statistically significant difference (*p* < 0.05). L is linear, and Q is quadratic effects for diet SPR. TVFA = total volatile fatty acid; Branched-chain volatile fatty acid = isobutyrate + isovalerate; NH_3_-N = ammonia nitrogen.


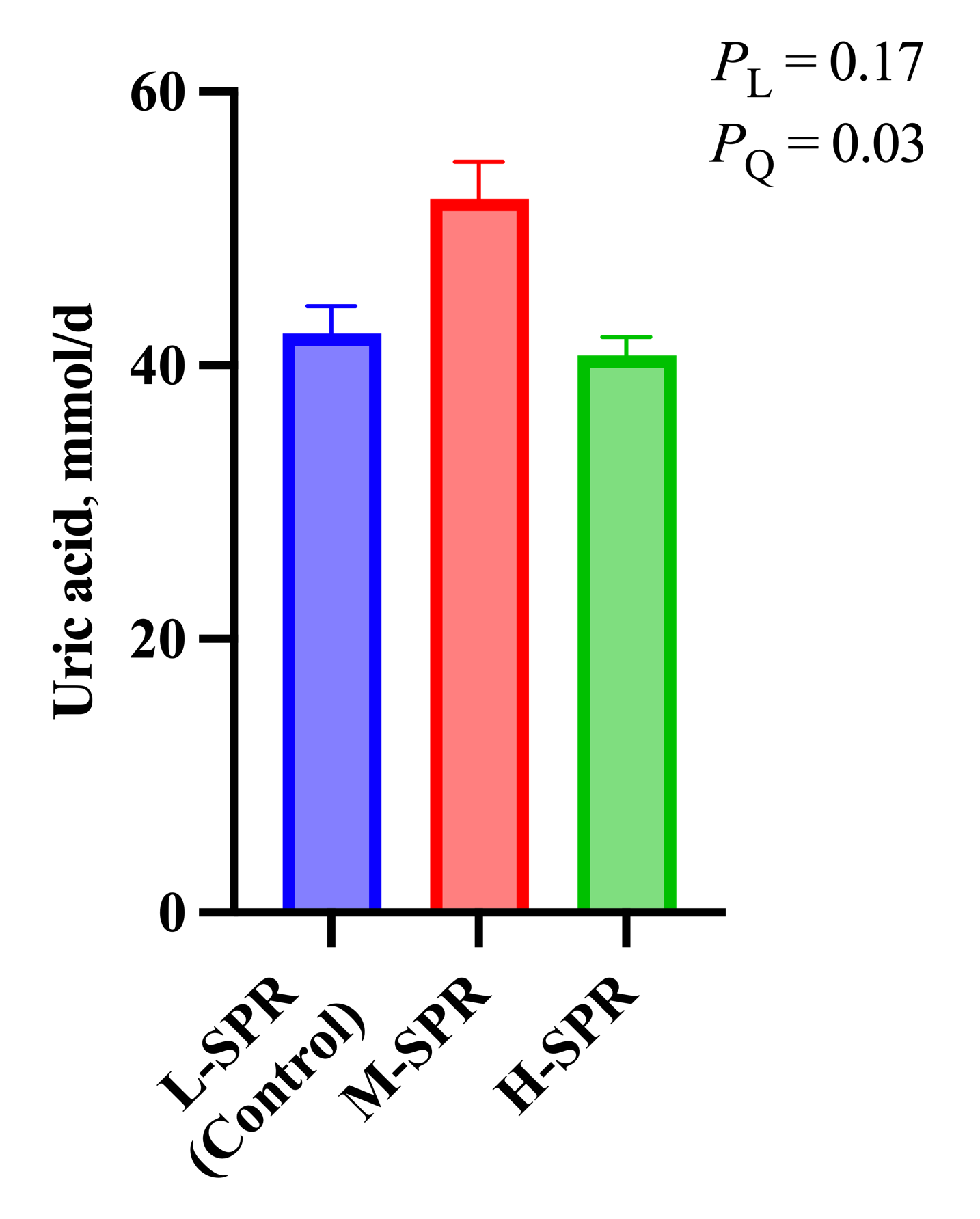

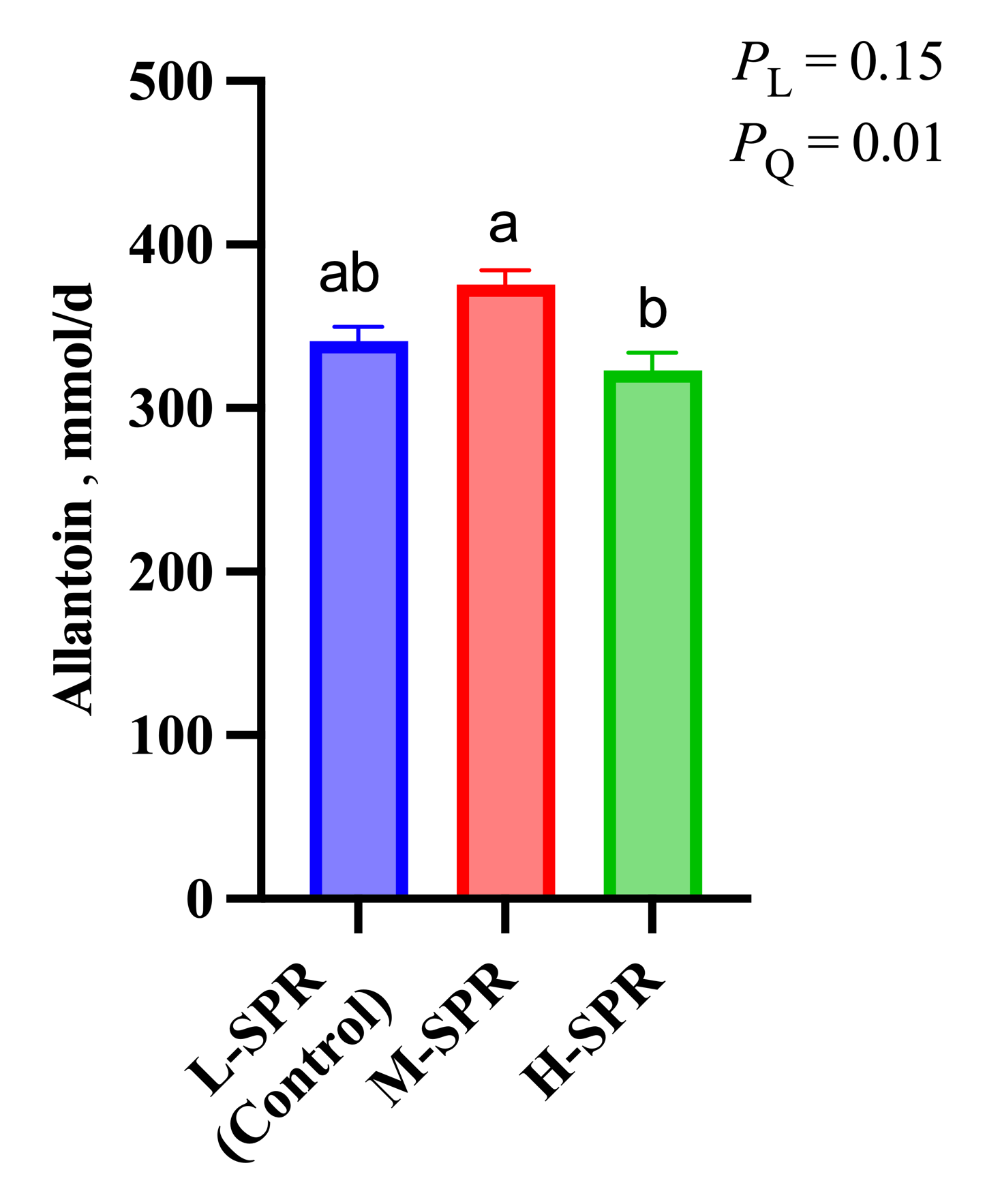

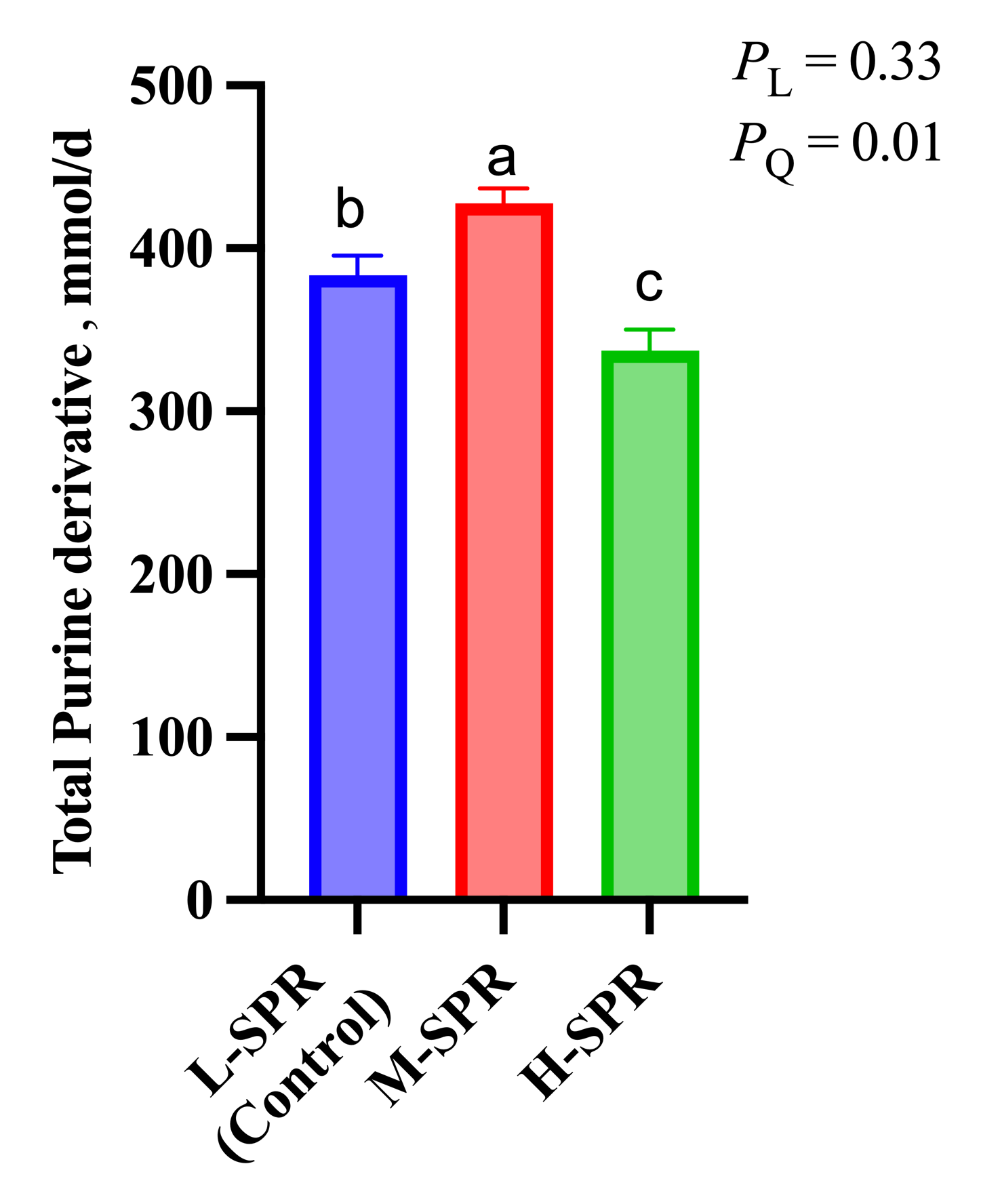

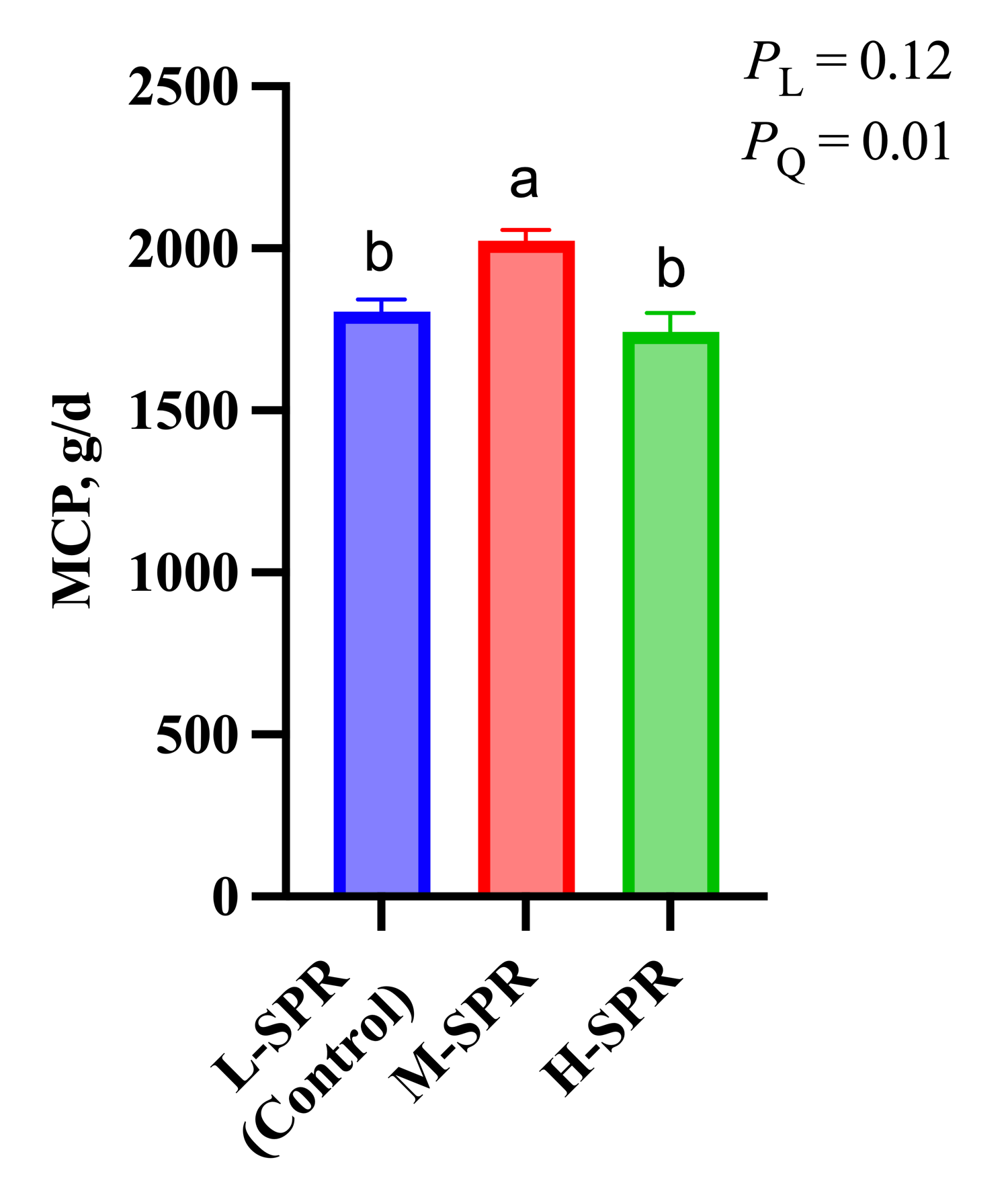

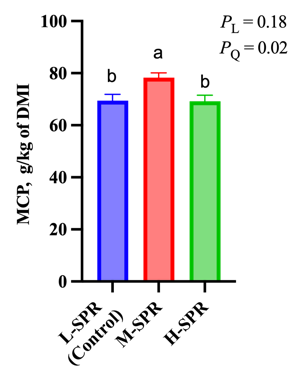

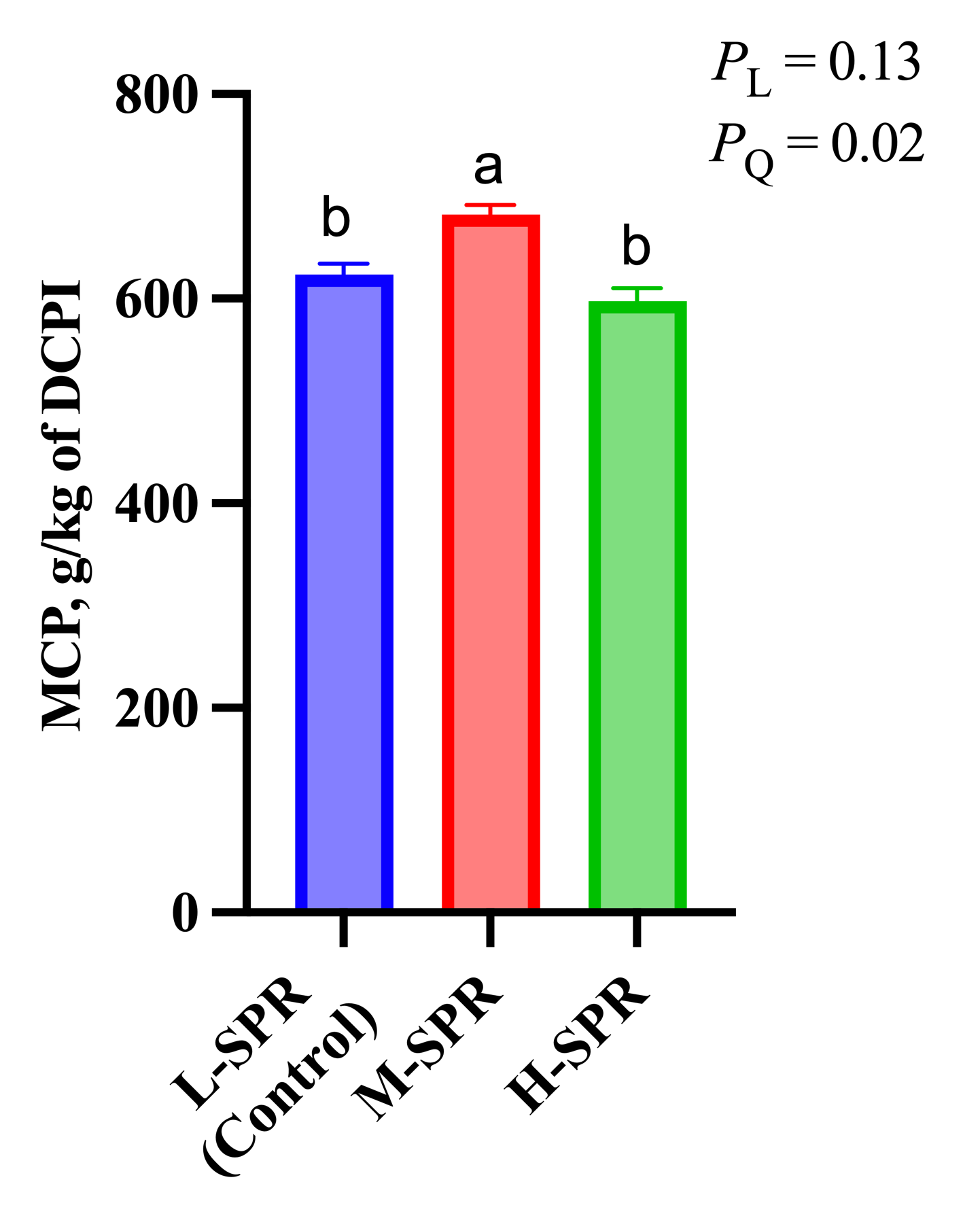


**Figure 4.** Effect of dietary rumen-degradable starch to rumen-degradable protein ratio (SPR) on urinary purine derivatives excretion in mid-lactating Holstein cows. Error bars indicate measure of variation within the dietary SPRs. Different letters (a–c) indicate statistically significant difference (*p* < 0.05). L is linear, and Q is quadratic effects for diet SPR. MCP = microbial crude protein; DMI = dry matter intake; DCPI = digestible CP intake.


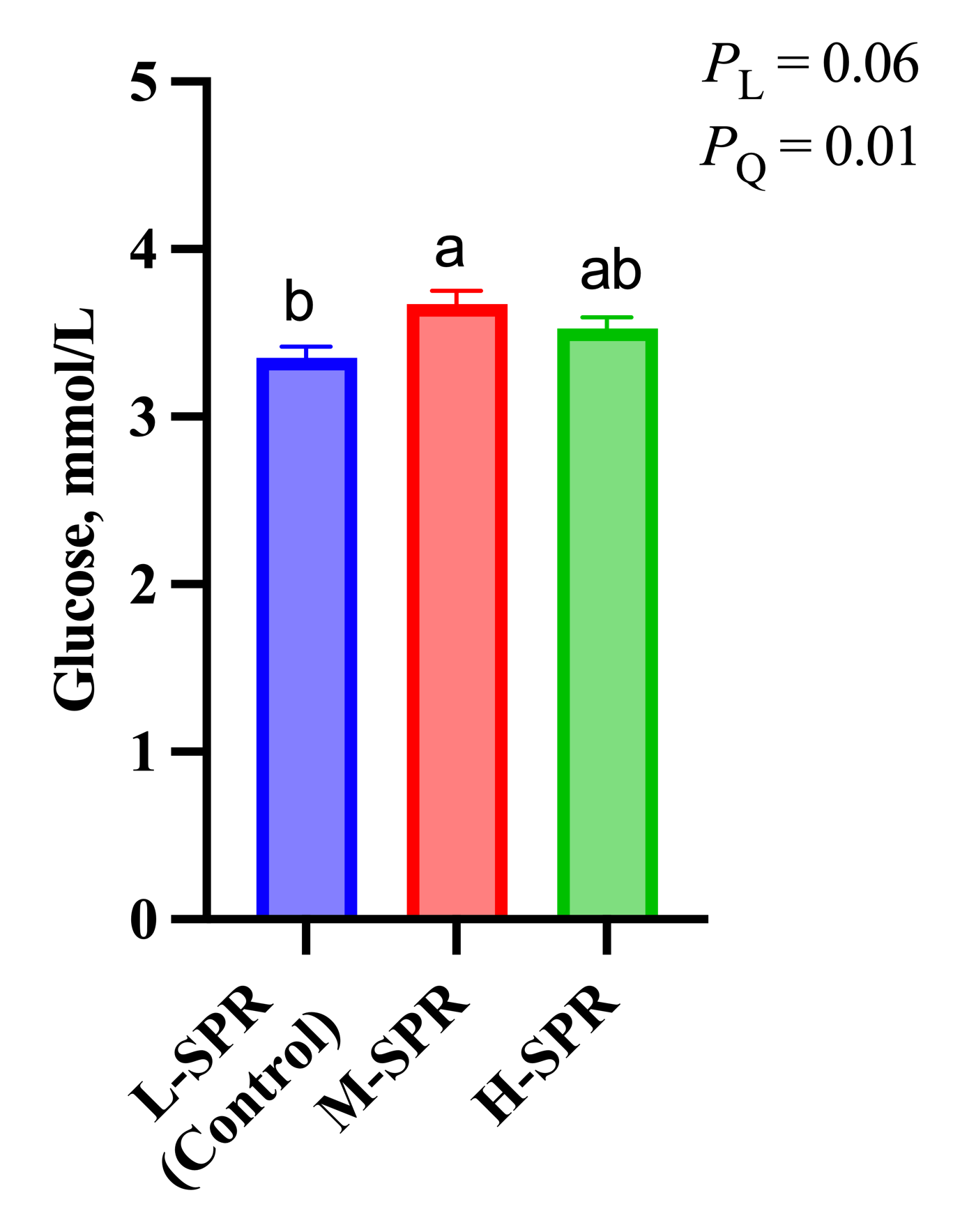

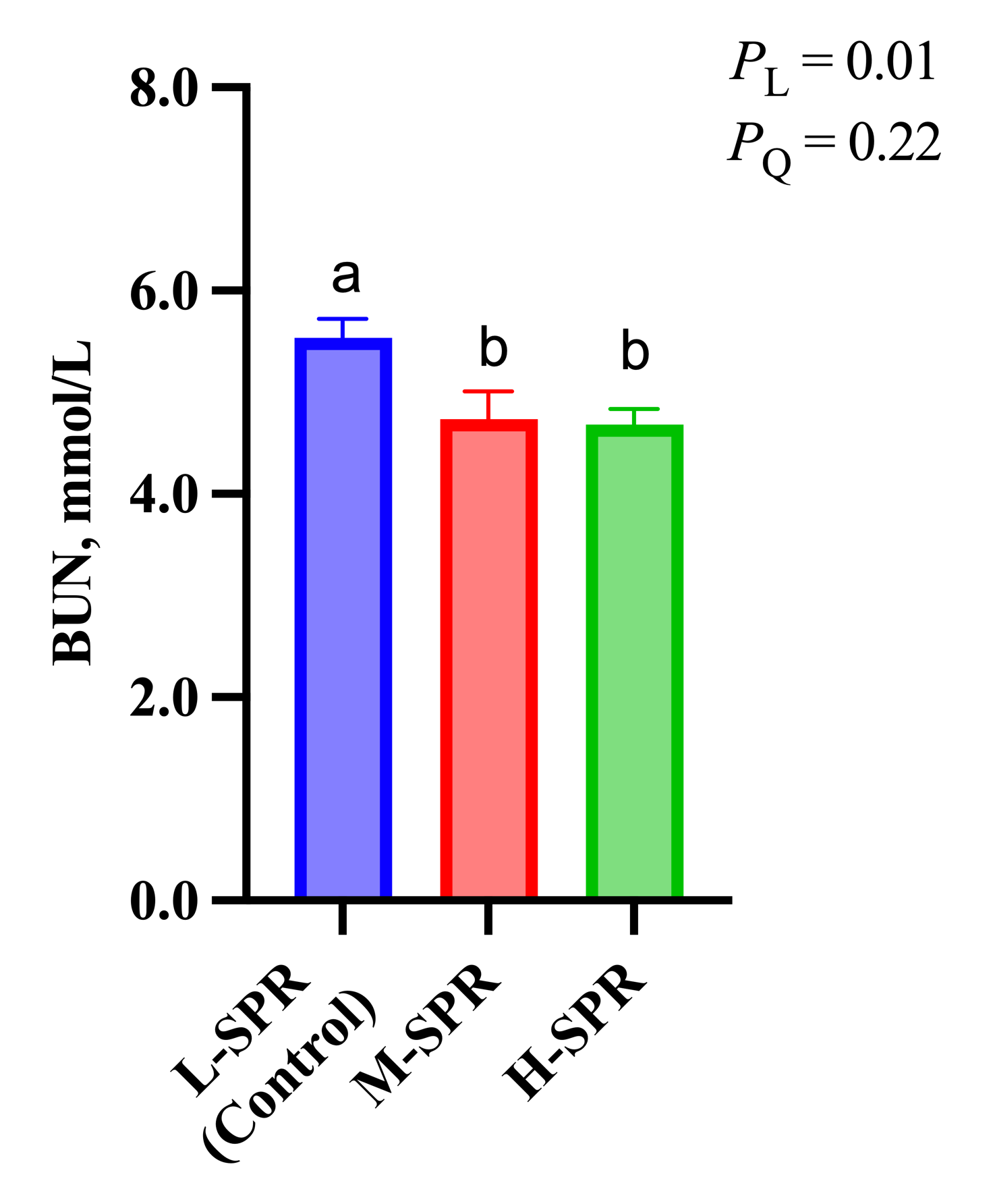

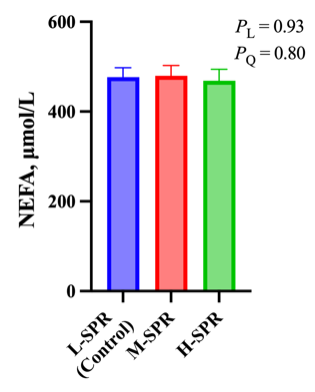

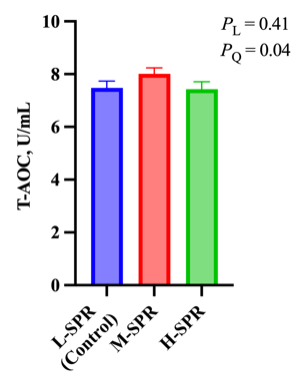

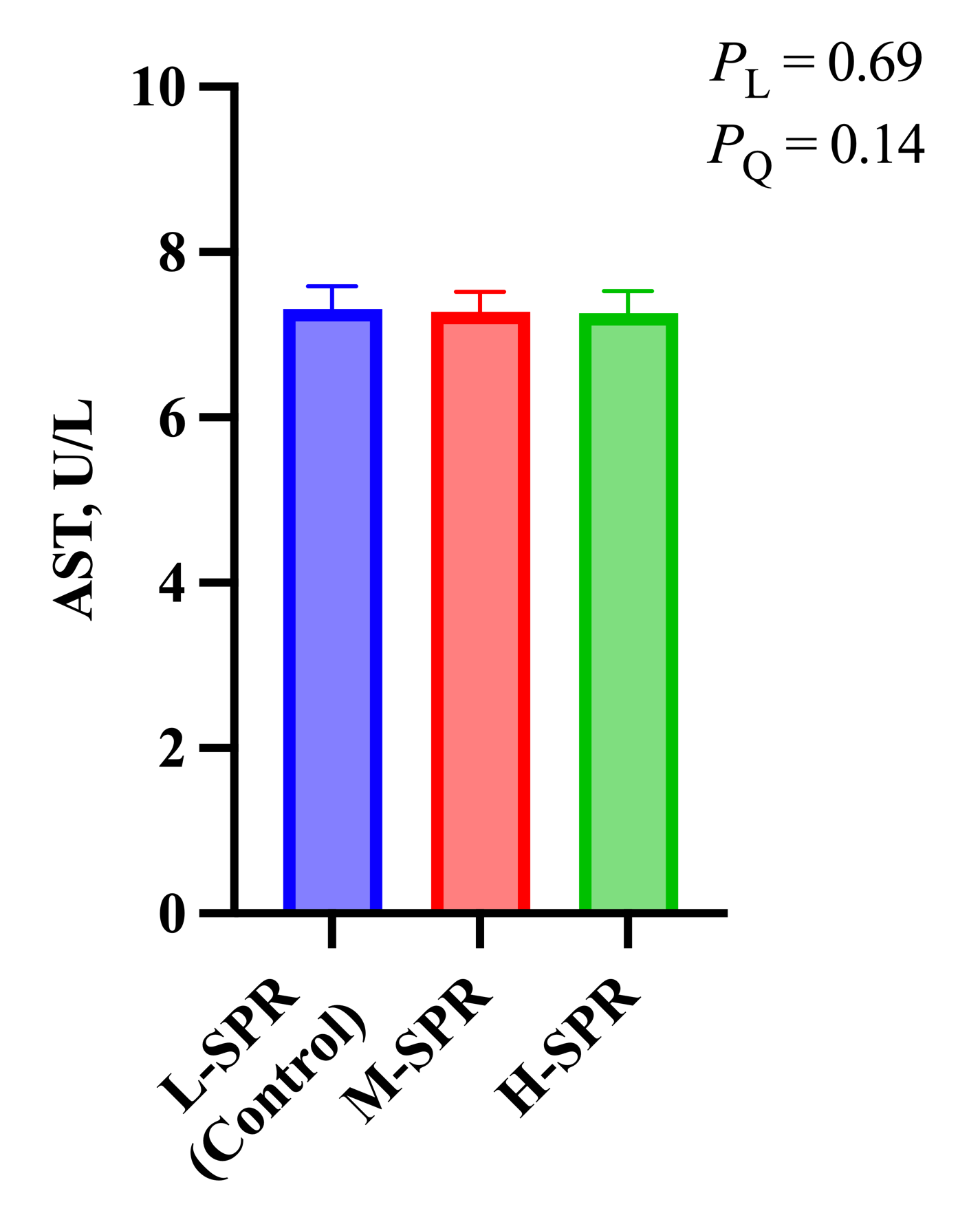

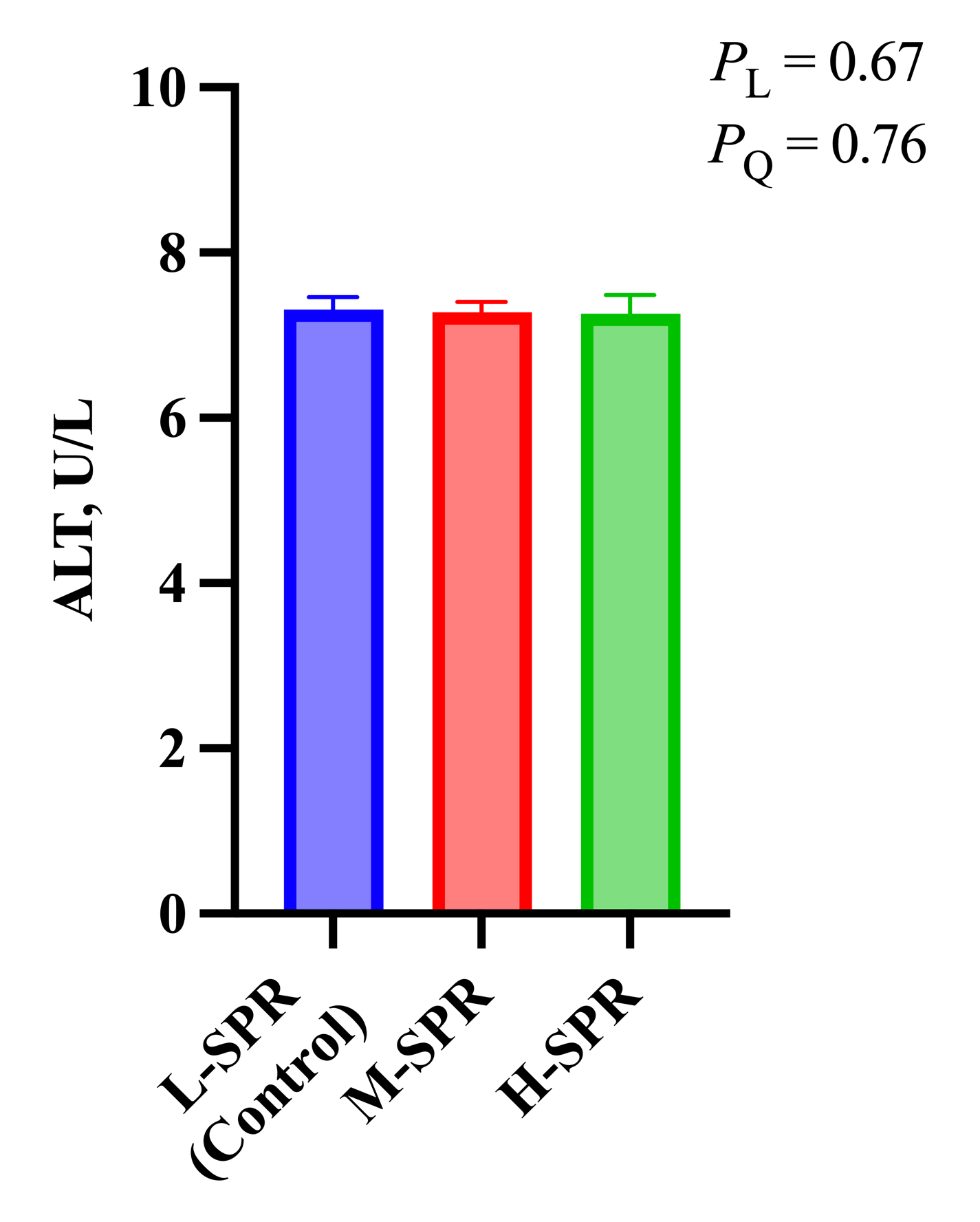

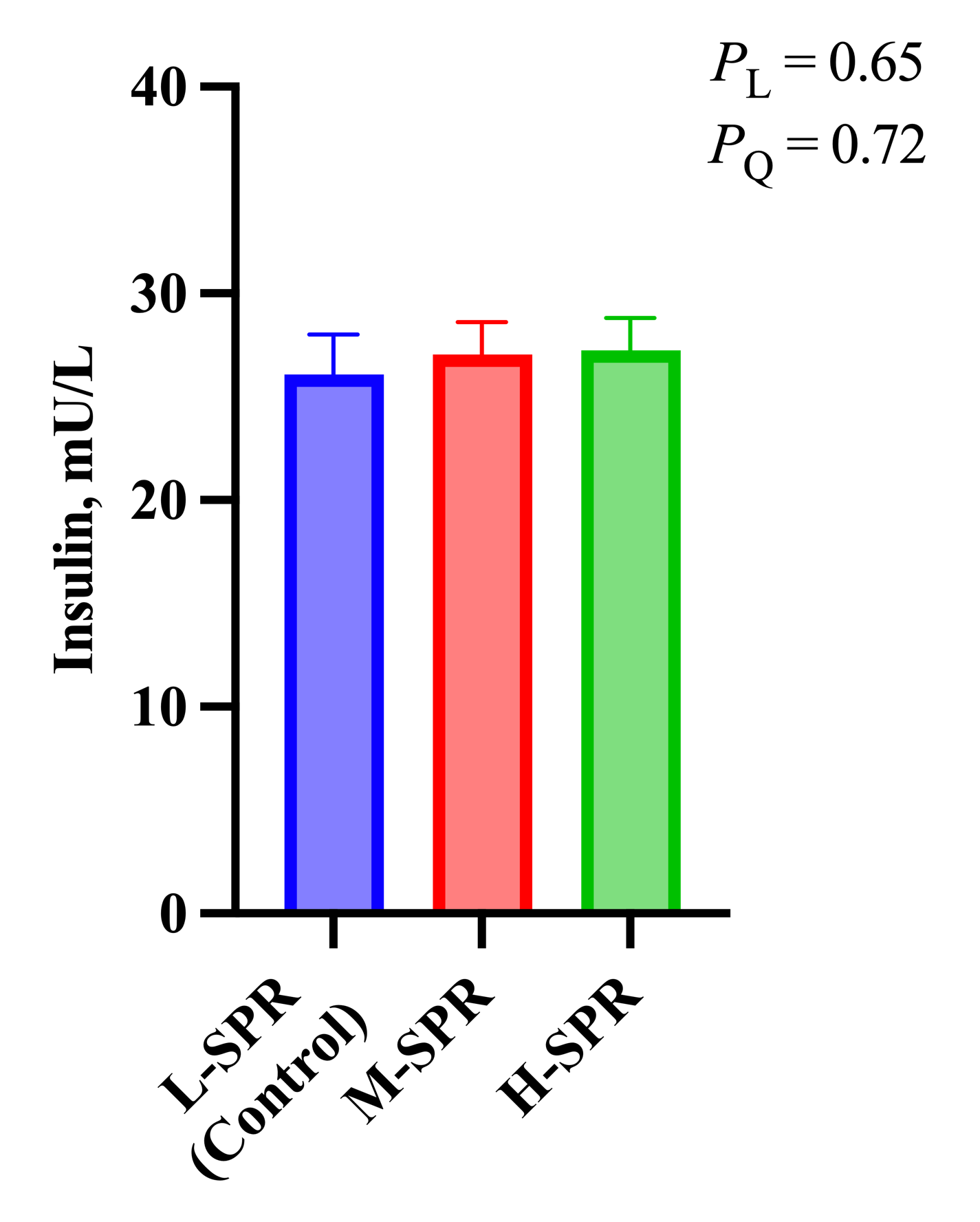

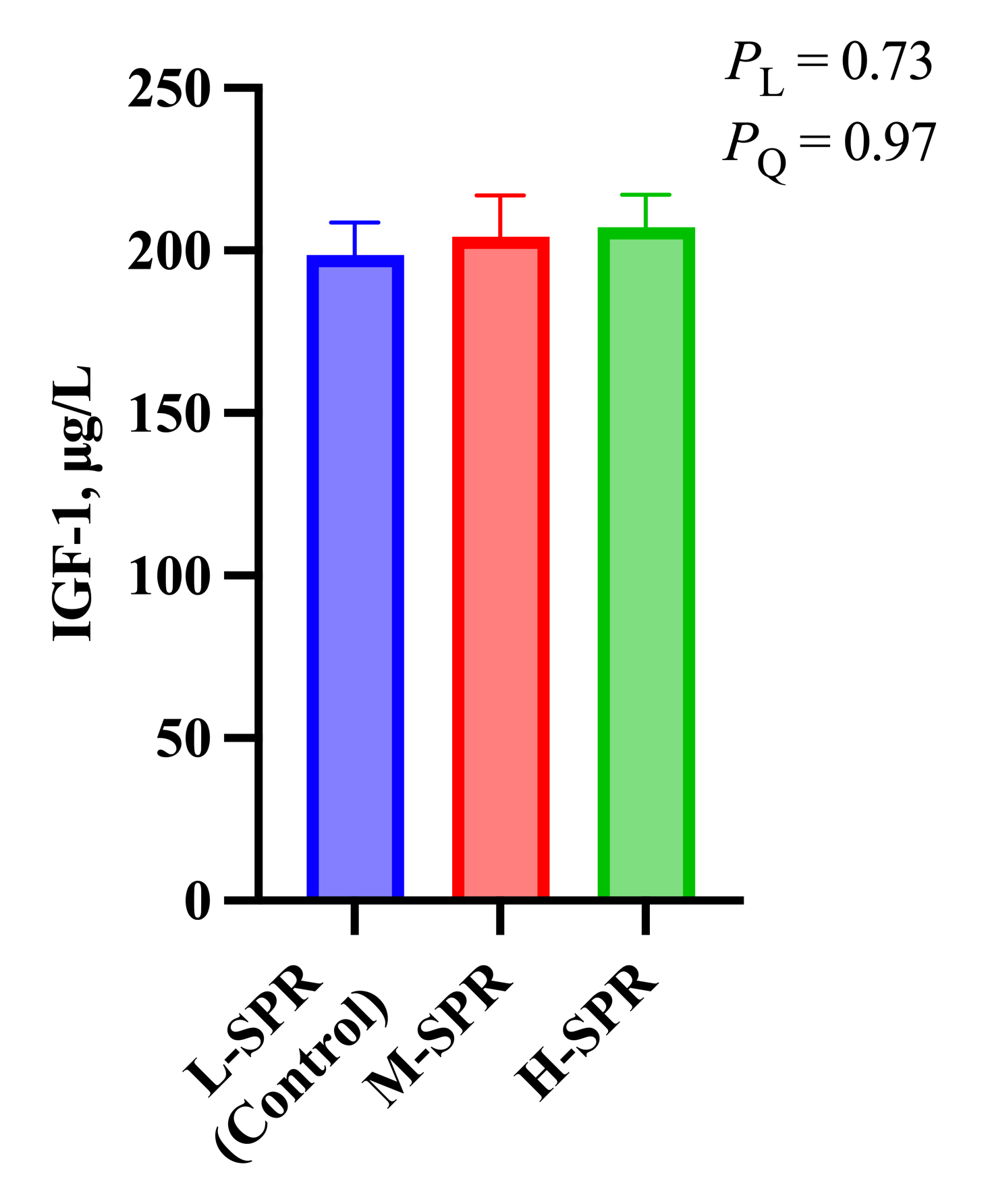


**Figure 5.** Effect of dietary rumen-degradable starch to rumen-degradable protein ratio (SPR) on blood indicators in mid-lactating Holstein cows. Error bars indicate measure of variation within the dietary SPRs. Different letters (a–b) indicate statistically significant difference (*p* < 0.05). L is linear, and Q is quadratic effects for diet SPR; BUN, blood urea nitrogen; NEFA, nonesterified fatty acid; T-AOC, total antioxidant capacity; AST, aspartate aminotransferase; ALT, alanine aminotransferase; IGF-1, insulin-like growth factor-1.


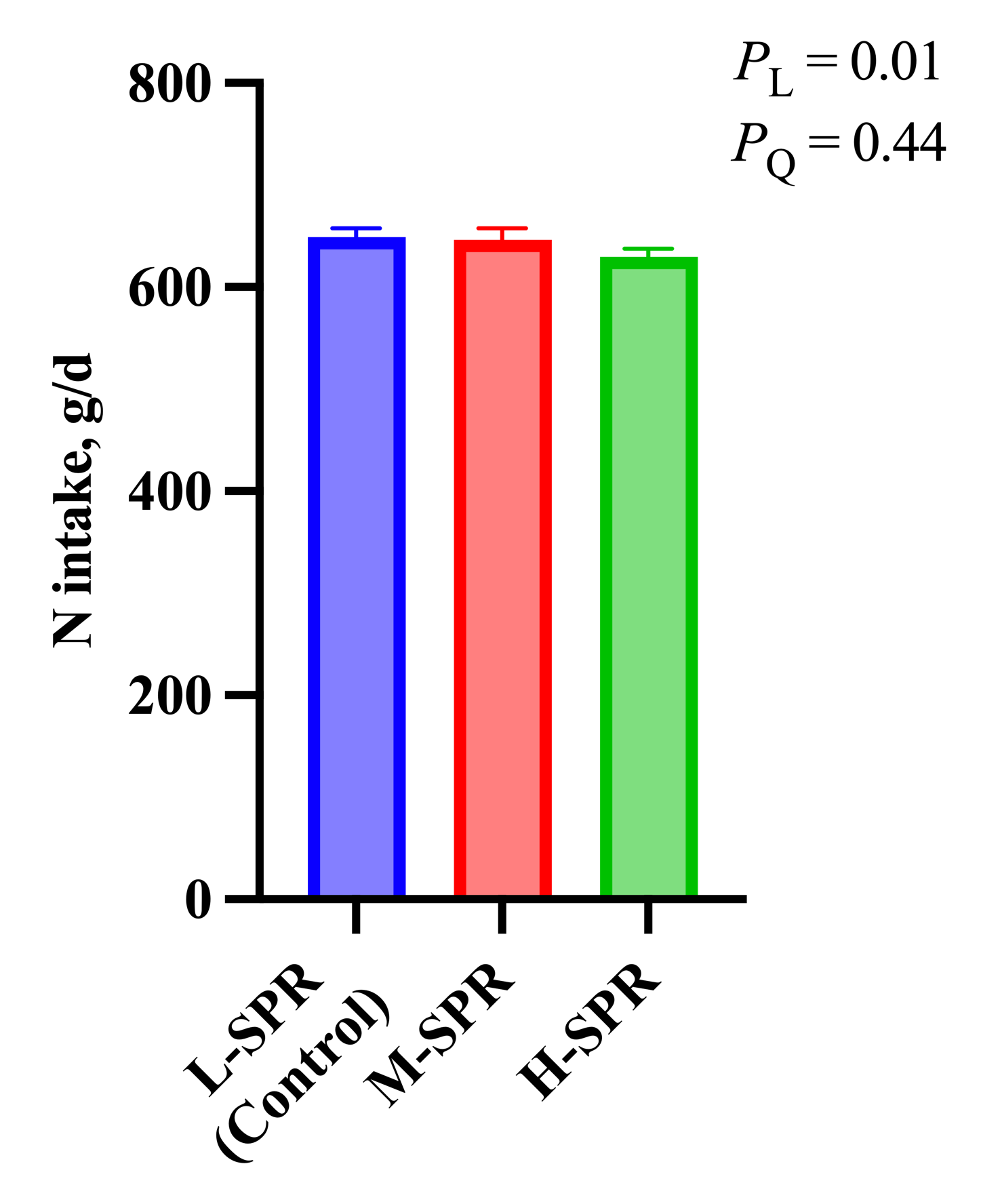

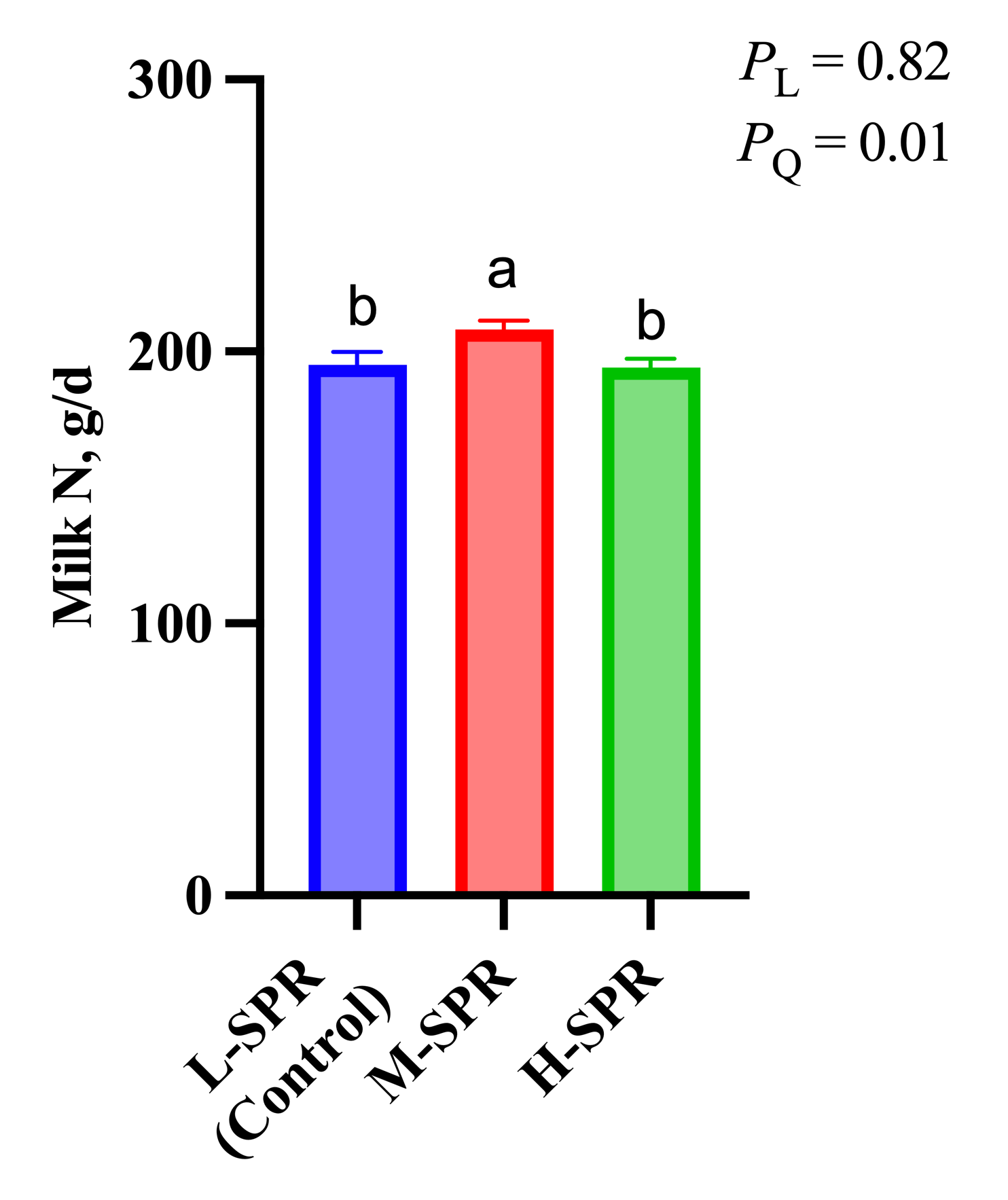

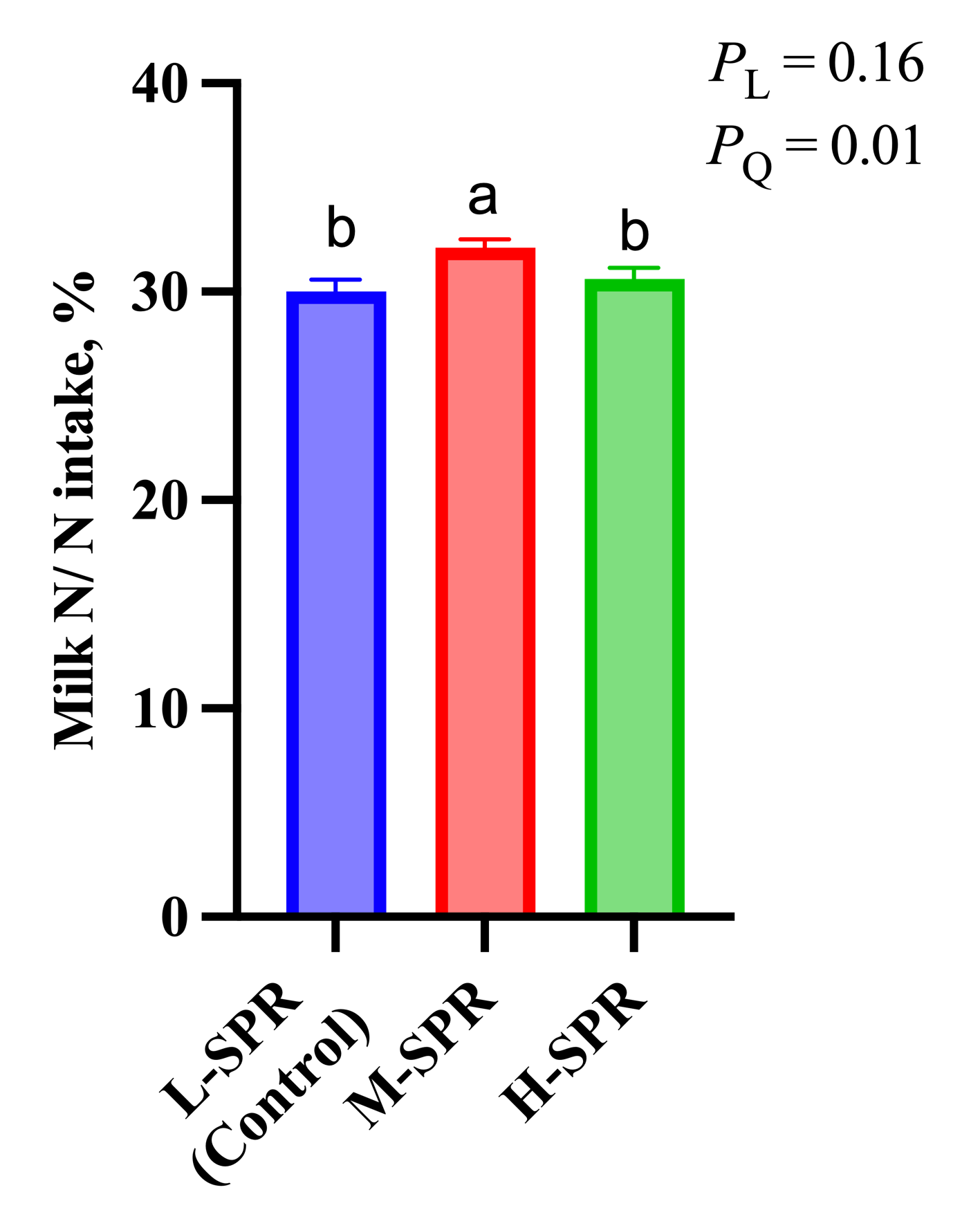

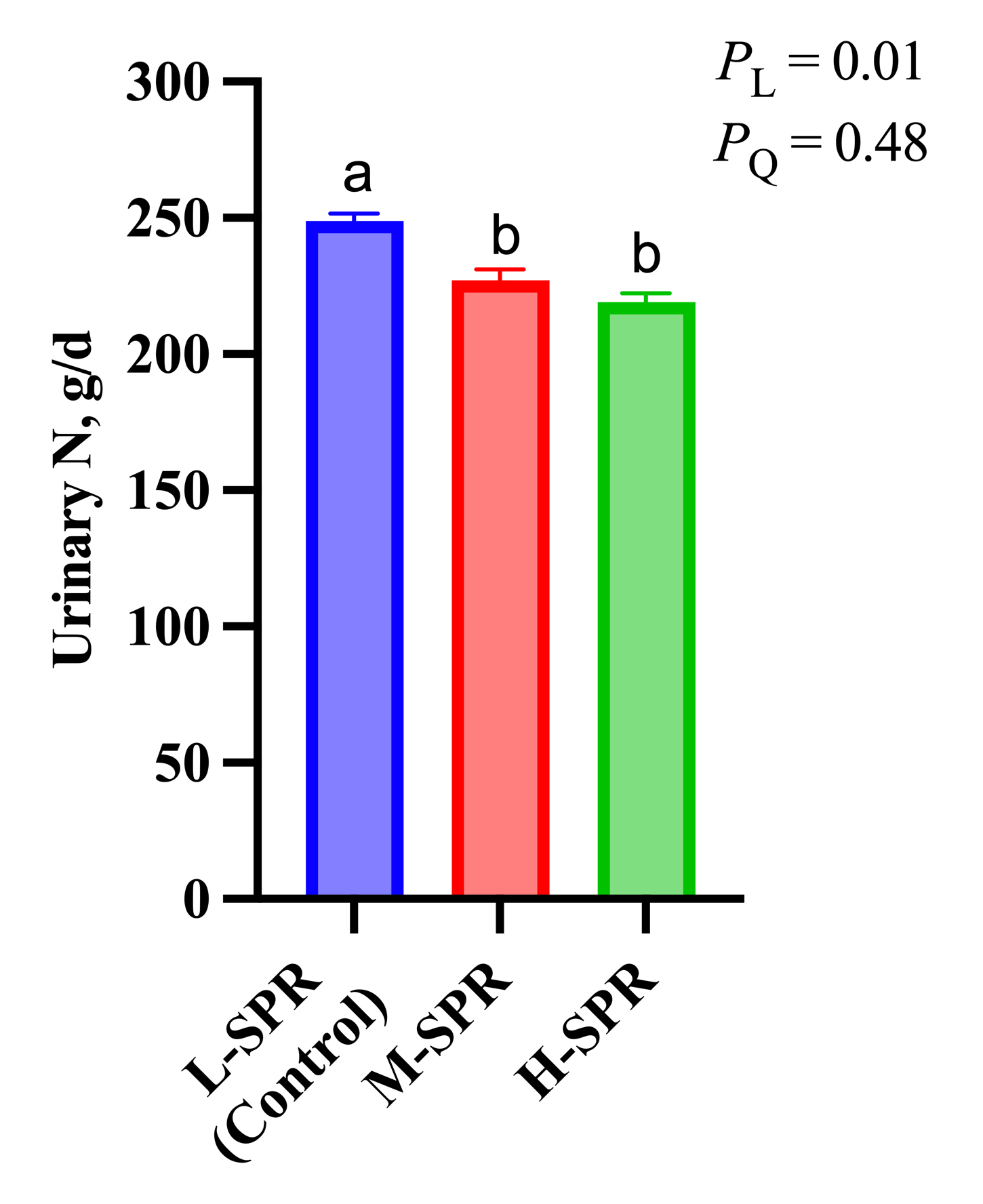

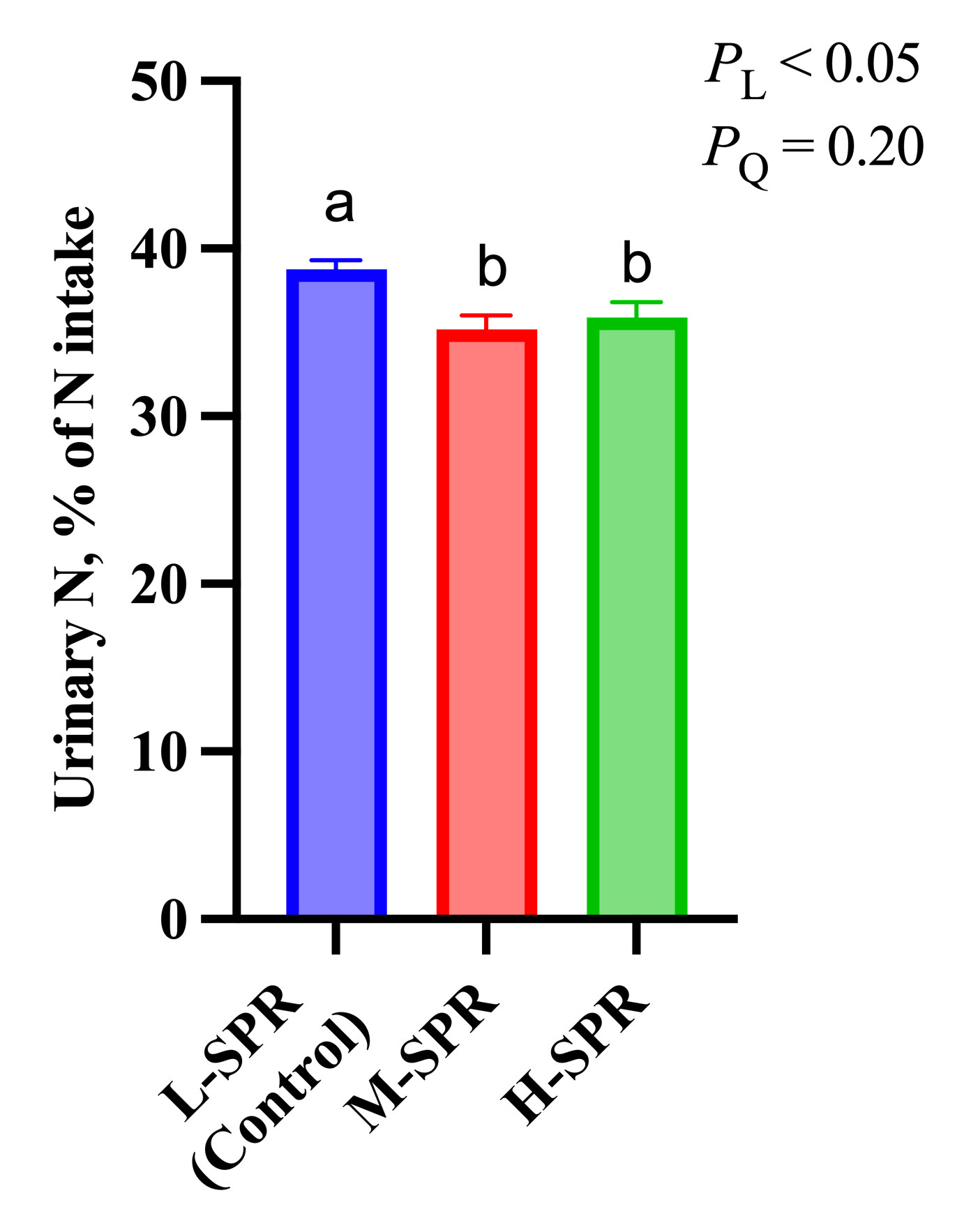

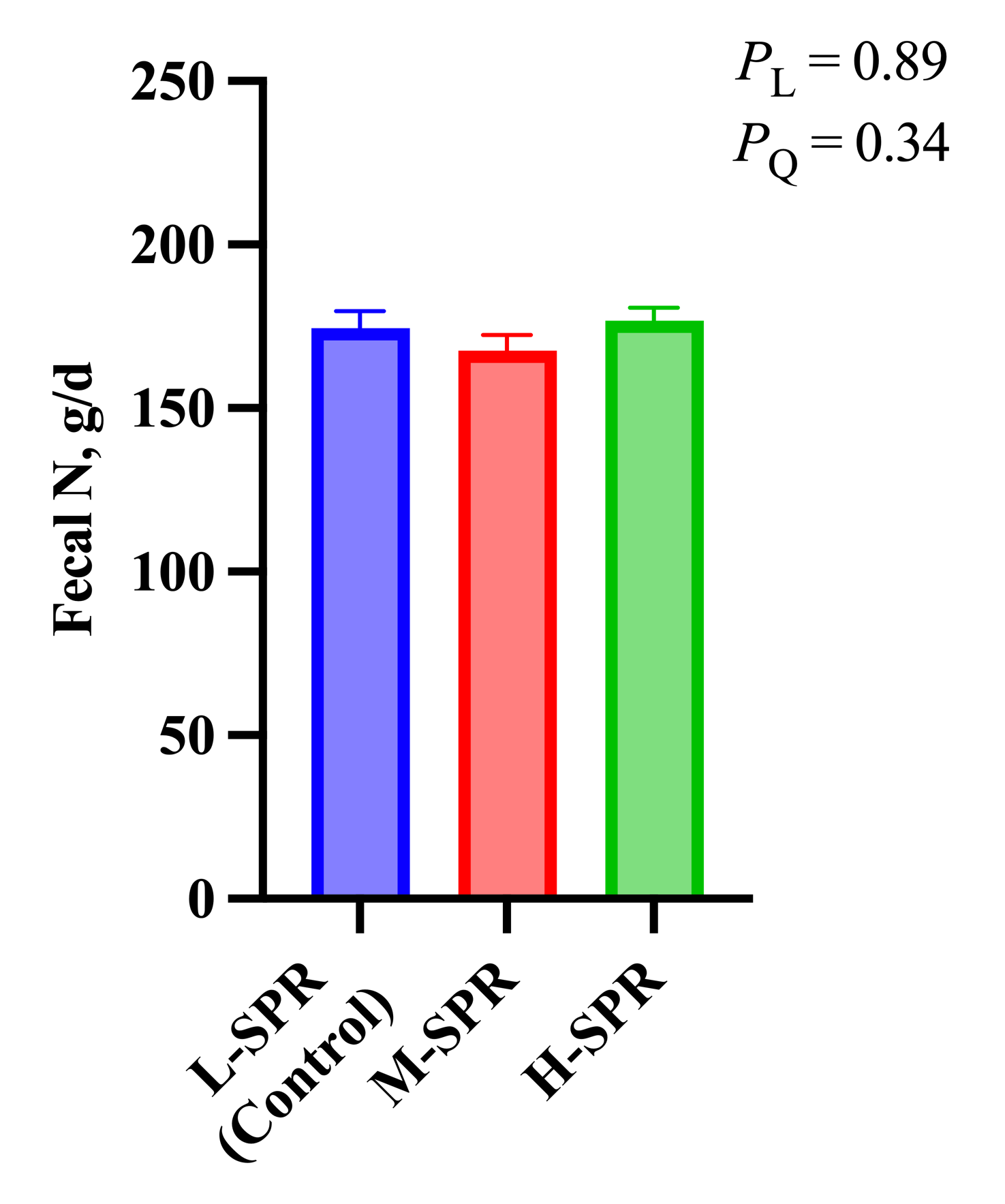

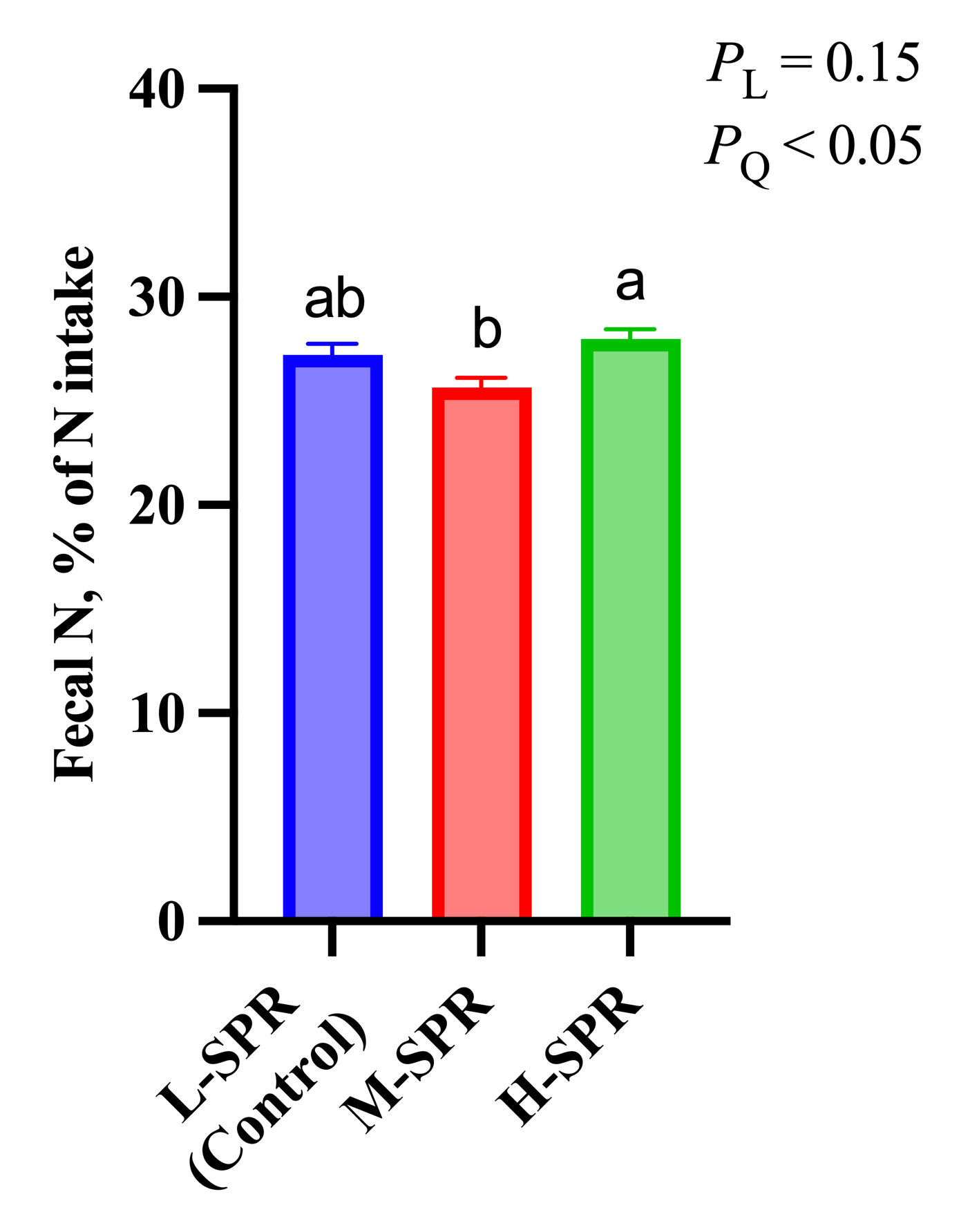

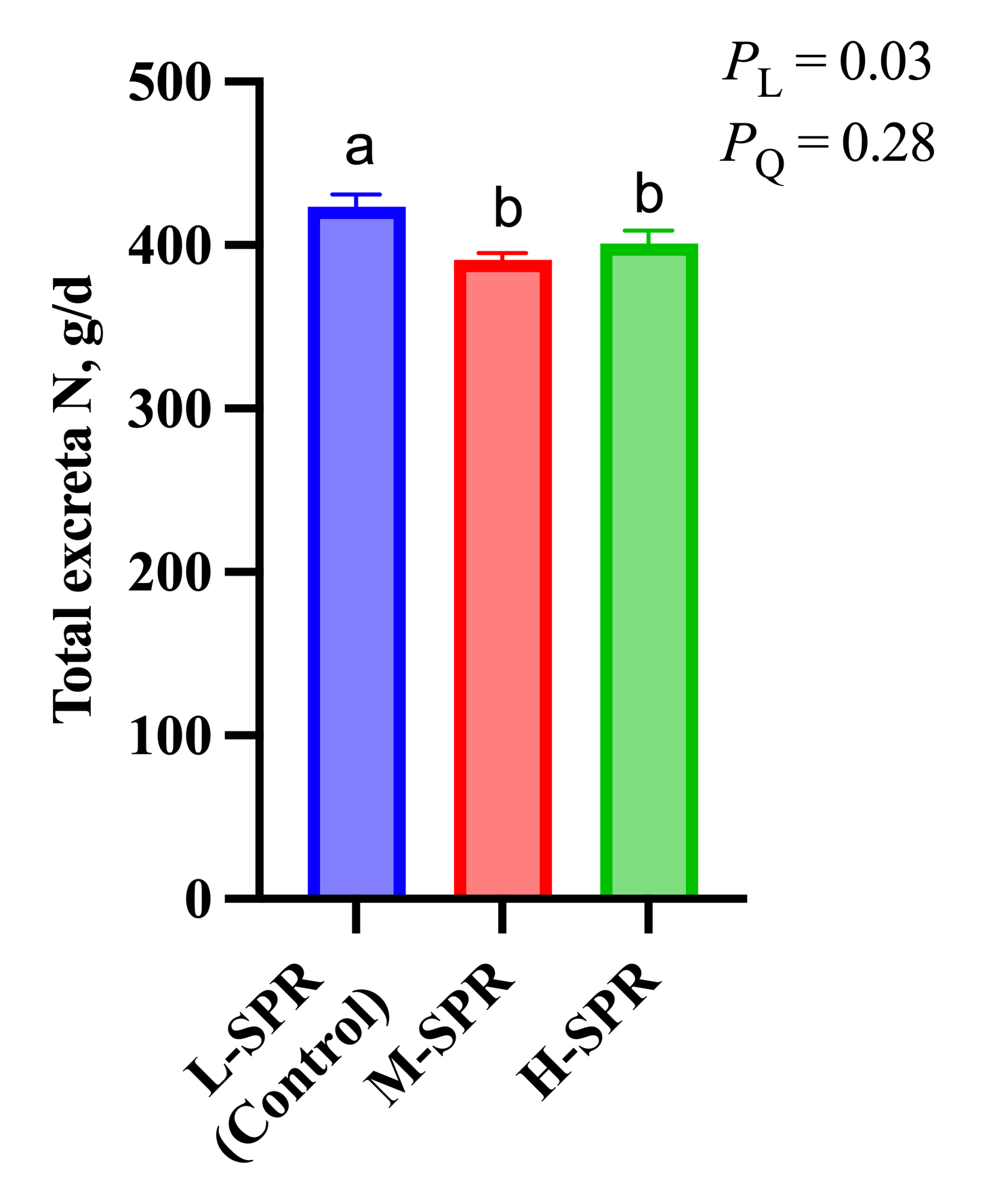


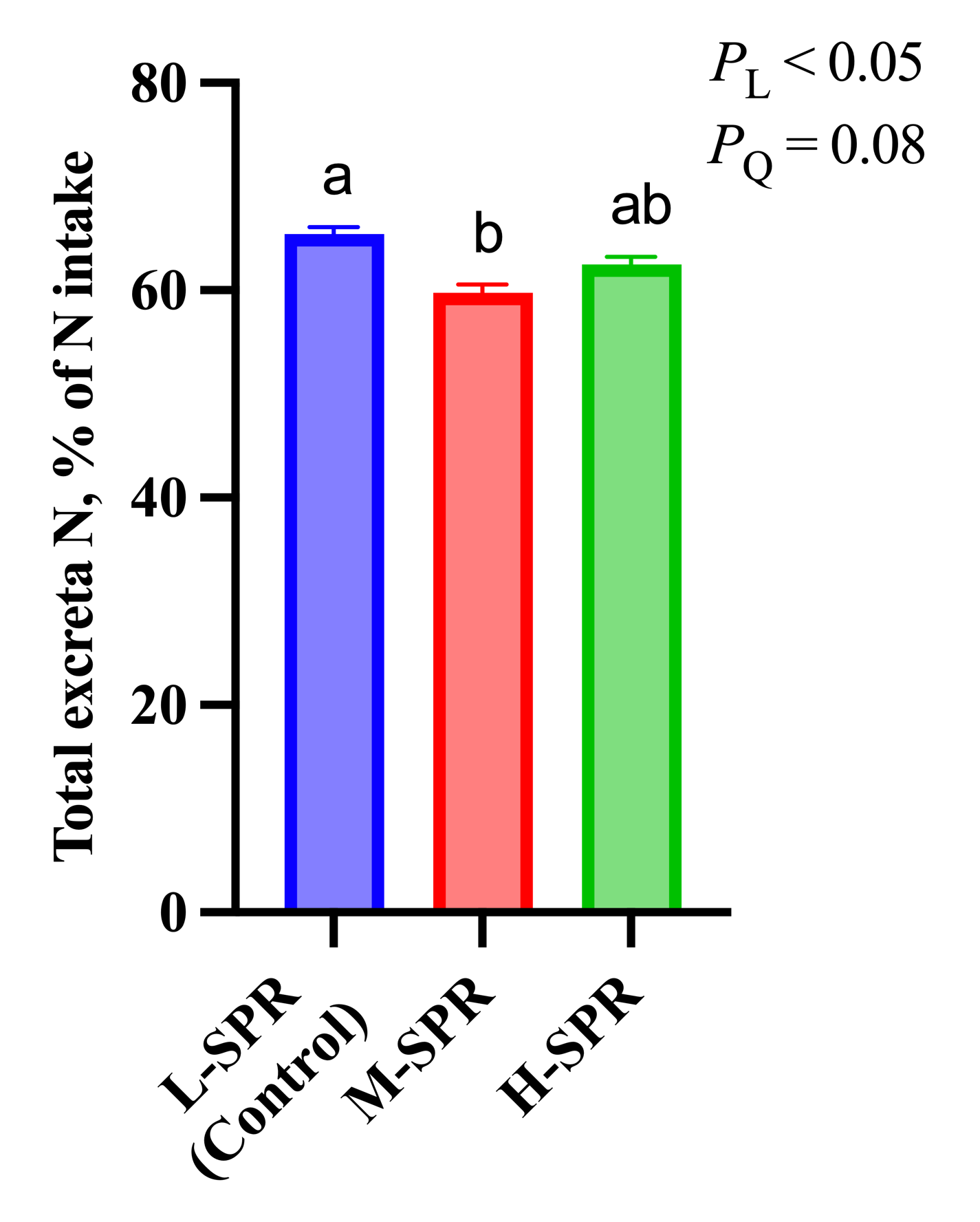

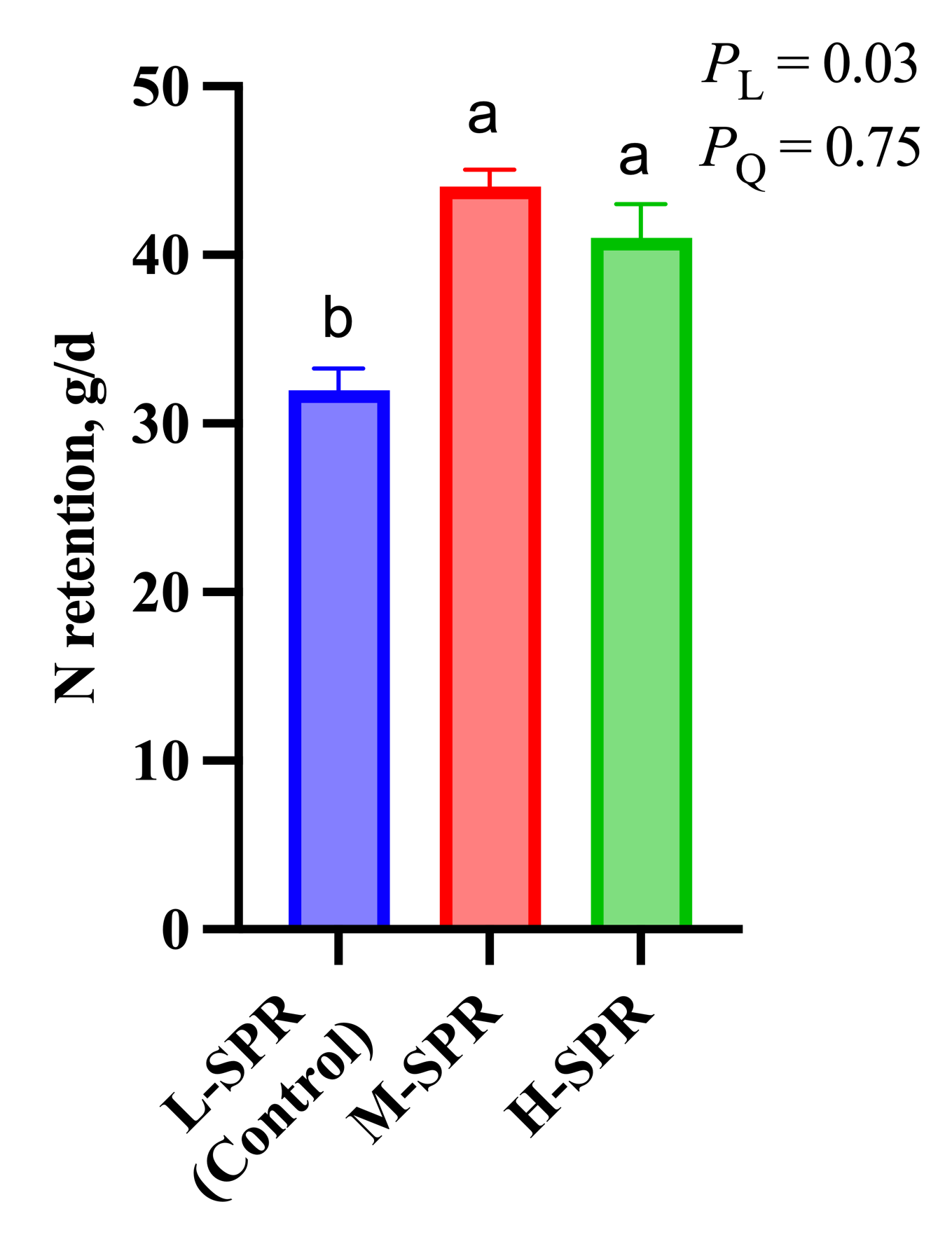

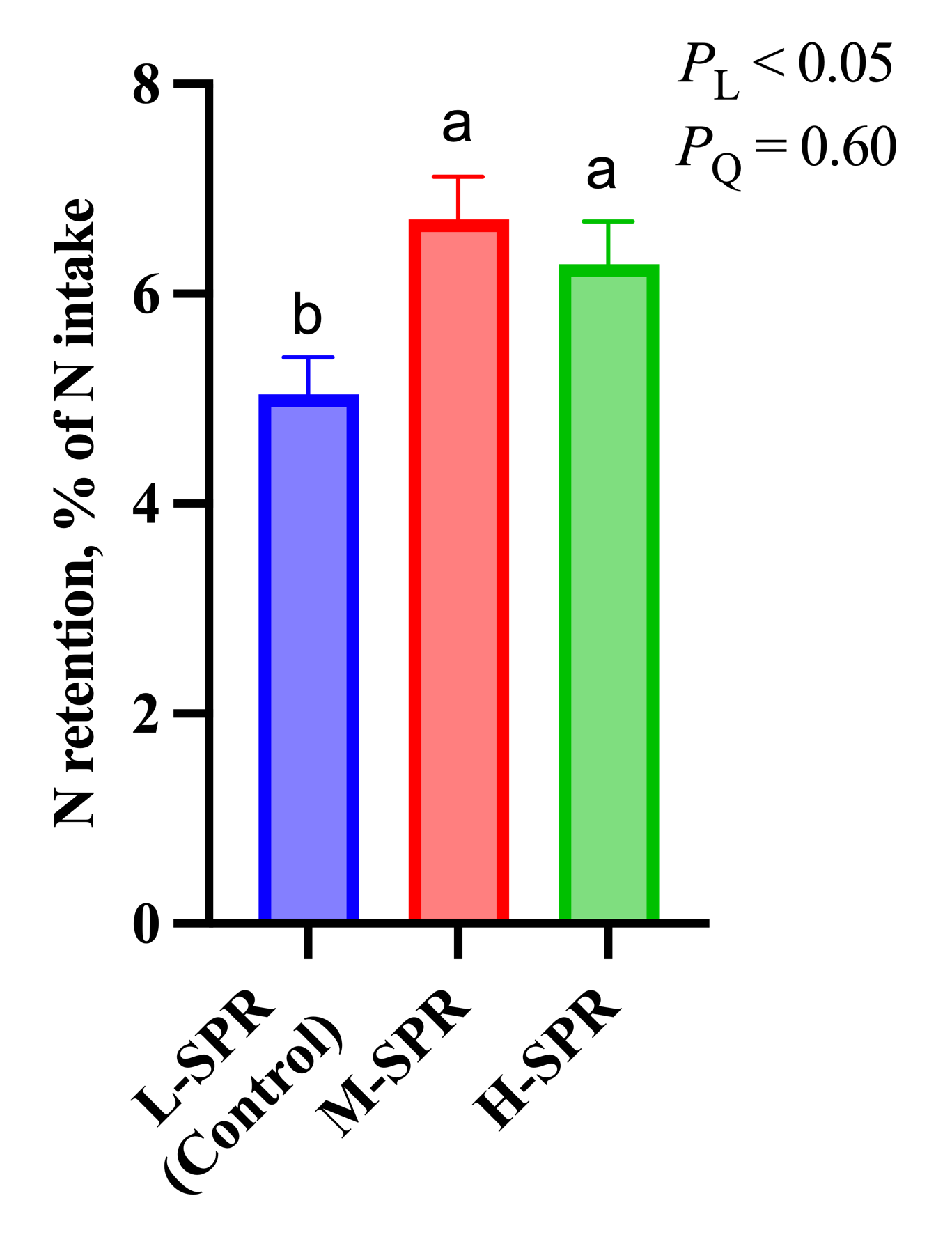


**Figure 6.** Effect of dietary rumen-degradable starch to rumen-degradable protein ratio (SPR) on nitrogen partitioning in mid-lactating Holstein cows. Error bars indicate measure of variation within the dietary SPRs. Different letters (a–b) indicate statistically significant difference (*p* < 0.05). L is linear, and Q is quadratic effects for diet SPR; Milk N = milk crude protein ÷ 6.25; Total N excretion = fecal N + urinary N; N retention = N intake – milk N – urinary N – fecal N.
