# Supplementary material for: Optimizing dietary rumen-degradable starch to rumen-degradable protein ratio improves lactation performance and nitrogen utilization efficiency in mid-lactating Holstein dairy cows
Source: Front Vet Sci. 2024 Feb 29;11:1330876. doi: 10.3389/fvets.2024.1330876 (PMC10938912; doi:10.3389/fvets.2024.1330876)
Supplement: Supplementary file 2 [file Table_1.docx]

**Table S1.** Nutrient composition of ingredients（DM basis）

| Item | Corn  silage | Oat  hay | Alfalfa  hay | Ground  corn | SSBM | HSBM | Wheat  bran | Beet  pellets |
| --- | --- | --- | --- | --- | --- | --- | --- | --- |
| OM, % | 96.6 | 90.1 | 91.6 | 99.1 | 93.0 | 93.0 | 93.7 | 92.8 |
| CP, % | 8.50 | 7.30 | 19.4 | 9.2 | 49.2 | 49.5 | 19.5 | 11.2 |
| Starch, % | 34.1 | 6.67 | 3.76 | 65.6 | 3.50 | 3.52 | 22.6 | 9.62 |
| ERDCP, % of CP | 67.9 | 49.6 | 62.2 | 58.1 | 54.4 | 31.3 | 76.2 | 81.7 |
| ERDST, % of Starch | 81.7 | 64.8 | 60.0 | 60.3 | 65.1 | 46.9 | 65.7 | 84.6 |
| RDP, % | 5.77 | 3.62 | 12.04 | 6.38 | 25.77 | 15.50 | 14.86 | 9.14 |
| RDS, % | 27.8 | 4.32 | 2.26 | 39.6 | 2.28 | 1.65 | 14.9 | 8.14 |

^1^ ERDST, effective ruminal degradability for starch; ERDCP, effective ruminal degradability for crude protein; SSBM, solvent-extract soybean meal.

^2^ RDP (rumen-degradable protein, %) = CP (%) × ERDCP (%of CP); RDS (rumen-degradable starch, %) = Starch (%) × ERDST (% of starch).

^3^ HSBM (heat-treated soybean meal, Xingpu Feed Co. LTD, Harbin, China), the intestinal digestibility of RUP was 75.2%.
